# Association between hyperglycaemia, diabetes complications and development of fibrotic conditions among people living with type 1 and type 2 diabetes in England: a retrospective cohort study using UK Clinical Resource Datalink Aurum and Hospital Episode Statistics

**DOI:** 10.1136/bmjopen-2025-103426

**Published:** 2025-10-27

**Authors:** Georgie May Massen, Sarah Cook, Samuel T Moss, Rachel Chambers, Gisli Jenkins, Richard J Allen, Louise V Wain, Iain Stewart, Nick Oliver, Daniel L Morganstein, Jennifer K Quint, on behalf of the DEMISTIFI Consortium

**Affiliations:** 1 School of Public Health, Imperial College London, London, UK; 2 Department of Health Services Research and Policy, London School of Hygiene & Tropical Medicine, London, UK; 3 Division of Public Health and Epidemiology, University of Leicester, Leicester, UK; 4 NIHR Leicester Biomedical Research Centre, Leicester, UK; 5 Department of Respiratory Medicine, University College London, London, UK; 6 National Heart and Lung Institute, Imperial College London, London, UK; 7 Department of Metabolism, Digestion and Reproduction, Imperial College London, London, UK; 8 Skin Unit, Royal Marsden Hospital NHS Trust, London, UK; 9 Department of Endocrinology, Chelsea and Westminster Healthcare NHS Trust, London, UK

**Keywords:** EPIDEMIOLOGY, Fibrosis, DIABETES & ENDOCRINOLOGY

## Abstract

**Abstract:**

**Objective:**

Fibrosis is a pathological feature that can occur in a wide range of diseases including diabetes mellitus. We investigated whether in people with type 1 (T1DM) or type 2 diabetes mellitus (T2DM), glycaemia or diabetes-related complications are associated with fibrotic diseases.

**Design and setting:**

Retrospective cohort study using UK Clinical Resource Datalink (CPRD) Aurum and Hospital Episode Statistics.

**Participants:**

We included people with prevalent T1DM or T2DM as of 31 December 2015 (recorded in CPRD Aurum), eligible for linkage with Hospital Episode Statistics and followed up for 3 years.

**Outcome measures:**

We defined diabetes status using blood/urine biomarkers and complications. In the T2DM cohort, we also investigated exposures of hyperglycaemia, insulin resistance and metformin prescription. Fibrotic condition diagnoses were determined from both primary and secondary care records. Logistic regression analyses were undertaken to understand the strength of association between diabetes status/diabetic complications and fibrotic conditions, respectively.

**Results:**

The T1DM cohort consisted of 9669 people while the T2DM cohort included 504 066 people. In T1DM, we found that albuminuria was associated with lung fibrosis (ORadj: 2.07, 99% CI 1.35 to 2.17), and microvascular complications were associated with atherosclerosis (ORadj: 1.81, 99% CI 1.18 to 2.77) and cardiomyopathy (ORadj 1.53, 99% CI:1.15 to 2.04). In the T2DM cohort, both glycaemia above target and diabetes complications were associated with most fibrotic conditions.

**Conclusions:**

Within the T1DM population, no consistent association between diabetes status and all fibrotic diseases was observed. More research is required to understand whether the association between diabetes complications and fibrotic diseases is due to shared risk factors or whether glycaemia in T2DM may be influenced by fibrotic pathology.

**STRENGTHS AND LIMITATIONS OF THIS STUDY**

- Large cohort of everyone with type 1 or type 2 diabetes recorded in a large representative electronic healthcare database.
- Multiple exposures were explored, defined using both clinical diagnoses and test results.
- We were unable to define drug adherence and instead used prescription data as a proxy.

## Introduction

 Multiple long-term conditions (MLTCs) are associated with decreased quality of life, increased risk of mortality and people with MLTCs are more likely to die prematurely.^1^,^5^ MLTCs are a research priority, to minimise risks to patients by understanding potential effects of polypharmacy, and inform changes to healthcare structures and practices.^6^,^9^

Fibrotic conditions are characterised by excessive, uncontrolled deposition of extracellular matrix, leading to impaired organ function and failure.^10^,^12^ Fibrosis is associated with ageing and it is thought that the prevalence of fibrotic conditions will continue to increase.^13 14^ Fibrotic multimorbidity is defined as the presence of two or more fibrotic conditions in the same individual.^15^ A recent Delphi survey established consensus for 265 diseases as being fibrotic; diabetes mellitus reached consensus as ‘sometimes but not always’ being a fibrotic condition.^16^ As a result, we have further investigated whether more severe disease (using complications and treatments as a proxy) was associated with fibrotic conditions. We hypothesised that more significant hyperglycaemia, attenuated response to hypoglycaemic therapies and greater propensity to complications may be associated with fibrosis. By conducting this retrospective cohort study, we aimed to explore whether diabetes complications and severity are associated with fibrotic conditions.

Therefore, we looked to further understand if type 1 diabetes mellitus (T1DM) or type 2 diabetes mellitus (T2DM) was associated with fibrotic conditions with respect to diabetes status and complications. We investigated whether microvascular complications of retinopathy, neuropathy and nephropathy were associated with fibrotic conditions. As such, retinopathy and neuropathy were defined using codes present in primary care records (as this cannot be confirmed through testing), while nephropathy was defined as persistently increased albumin to creatinine ratio (ACR) and persistently decreased estimated glomerular filtration rate (eGFR). We further looked to explore whether evidence of kidney damage in people with diabetes was associated with fibrotic conditions. We did this by using single and multiple test results of ACR and eGFR. We looked to understand in people with T2DM, whether glucose above target was associated with fibrotic conditions. Glucose above target was defined as glycated haemoglobin (HbA_1c_) ≥7.5%/ 58 mmol/mol. It was also hypothesised that insulin-dependent diabetes may be associated with fibrotic conditions.^17 18^ Insulin-dependent diabetes was defined in the population with T2DM who were prescribed insulin. We were also interested in exploring whether metformin was protective of fibrotic conditions in people with T2DM.^19^,^21^

## Methods

### Data source and study population

To conduct this retrospective cohort study, we used the December 2023 build of the Clinical Practice Research Datalink Aurum database. Aurum contains pseudonymised primary care data covering 24% general practitioner (GP)-registered patients in England^22^ and is representative in terms of age, sex, ethnicity, geographical region and social deprivation.^23^ Primary care data were linked to secondary care data from NHS England’s Hospital Episode Statistics (HES) Admitted Patient Care (APC) database and Index of Multiple Deprivation data (IMD).

Diabetes diagnoses were identified in primary care records, codelists used to identify T1DM and T2DM are available on GitHub. We conducted two prospective cohort studies, using the data of people with either T1DM or T2DM, as of the 31 December 2015.

Broad inclusion criteria required that people had to be at least 18 years of age, have a recorded sex (the number of indeterminate cases was low and therefore excluded) and people had to have at least one record of T1DM or T2DM in their primary care records between the 1 January 2015 and 31 December 2015 (baseline period). More study-specific criteria required that people had to be enrolled at their GP before the 1 January 2015 and remain enrolled until at least 31 December 2018, the patient’s data had to be eligible for linkage to HES APC and IMD. Two discrete populations (T1DM and T2DM, respectively) were used throughout this study and at no point combined (online supplemental figure 1). The index date was the 31 December 2015 (online supplemental figure 2).

### Exposures

#### Microvascular complications

A binary composite exposure of microvascular complications was defined using codes for neuropathy, retinopathy and persistently increased ACR and persistently decreased eGFR, see below.

#### Biomarkers of microvascular complications

In both cohorts, we explored whether elevated albumin creatinine ratio (ACR) and decreased eGFR were associated with fibrotic conditions. To investigate this, we used data recorded in the 2 years prior to the index date to identify at least one measurement of ACR and eGFR. Raised ACR was defined as >3 mg/mmol and decreased eGFR was defined as <60 mL/min (online supplemental figure 2).

We then explored how the strength of associations changed when using multiple readings of ACR and eGFR instead of a single instance. Persistently raised/decreased measurements were defined as a minimum of two measurements of ACR/eGFR, which were raised or decreased (respectively) in the 3-year period prior to index date (online supplemental figure 2).

#### Glucose above target

In the T2DM cohort, we explored the association between glycaemia and fibrotic conditions. We used records of the most recent measure of HbA_1c_ prior to the index date. A binary exposure was created, indicating if the most recent value was below 7.5%/58 mmol/mol, or if HbA _1c_≥ 7.5%/58 mmol/mol indicating glucose above target.

#### Prescribed medications

To understand whether insulin-treated T2DM was associated with fibrotic conditions compared with T2DM not requiring insulin, we defined a binary exposure of insulin prescription; one indicated evidence of at least one prescription for insulin in the year prior to index date (online supplemental figure 2).

As metformin is commonly prescribed to people with T2DM, to help with control of blood glucose levels, we investigated whether prescription of metformin was protective of development of fibrotic conditions by creating a binary metformin exposure, defined as at least one prescription in the baseline period (online supplemental figure 2).

### Outcomes

Fibrotic conditions previously defined using a Delphi methodology were identified in both Clinical Resource Datalink Aurum and HES APC, respectively.^16^ All codelists used in the analysis of this paper are available from https://github.com/NHLI-Respiratory-Epi/Diabetes-FMM. The following groups of fibrotic conditions were included in the analysis: atherosclerosis, biliary fibrosis, blood vessel fibrosis, cardiomyopathy, integumentary fibrosis, intestinal/pancreatic fibrosis, liver fibrosis, reproductive fibrosis, lung fibrosis, skeletal fibrosis, systemic fibrosis, urinary fibrosis (inclusive of kidney fibrosis) and cardiac valve fibrosis.

### Statistical analysis

Baseline characteristics of age (continuous), sex (male/ female), smoking status (never smoker/ex-smoker/current smoker), deprivation (1, least deprived—5, most deprived, defined from IMD data) and prevalence of single and multiple fibrotic conditions were defined as of the index date. We report the point prevalence of fibrotic conditions as of the index date, we compared prevalences of fibrotic conditions in exposed (with diabetic complications or insulin-treated or glucose above target) and non-exposed (without diabetic complications, not insulin-treated and HbA_1c_ below target) groups, detailing when the percentage of people with the condition was more than double in the exposed group compared with the non-exposed group.We have suppressed small numbers to preserve anonymity and therefore do not report prevalences lower than 1%.

Multinomial logistic regression was applied to analyse the risk (referring to the relative risk ratio, RRR) of being diagnosed with one or more than one fibrotic condition (in the 3 years post index date), in the exposed groups compared with those in the non-exposed groups (reference group). Fully adjusted models included the following ‘core’ covariates: age, sex, smoking status, deprivation, hypertension severity and number of fibrotic conditions as of the index date.

We analysed associations between diabetic complications/status and each fibrotic condition, respectively. Multivariable logistic regression was applied to investigate the association between diabetic complications/status and diagnosis of fibrotic diseases in the 3 years after the index date. To account for multiple testing, the CI was increased to 99%. Fully adjusted models included adjustment for ‘core’ covariates. People with a fibrotic condition at baseline were excluded from the analysis where the outcome was the fibrotic condition that they had.

We further investigated whether the identified associations were due in part to other unmeasured covariates. To understand whether associations were explained by other chronic conditions, we additionally adjusted for Charlson Comorbidity Index (CCI). We looked to understand whether associations were due to oral corticosteroids (OCS); as such, we adjusted for OCS use in the baseline period (defining use as at least three prescriptions of OCS). To try to disentangle the effect of diabetes duration, we adjusted for duration of diabetes (defined as the amount of time between the first entry of a diabetes code and the index date) within sensitivity analysis. Body mass index (BMI) was not included in the primary analysis as it was missing in 7.1% and 11.5% of T1DM and T2DM cohorts, respectively. However, within a sensitivity analysis, we included BMI (defined as underweight, BMI<18.4, 18.5≤normal BMI≤24.9, 25.0≤overweight≤29.9, obese≥30.0) in logistic regression model.

### Patient and public involvement

This work is part of a larger grant investigating multiple organ fibrosis: DEfining MechanIsms Shared across mulTI-organ Fibrosis to prevent the development of long-term multimorbidity (DEMISTIFI). Those working on DEMISTIFI are committed to patient and public involvement and have engaged with patients and families with fibrosis across the course of this study.

## Results

We defined a cohort of 9669 people with T1DM and 504 066 people with T2DM as of the index date.

**Figure 1:**
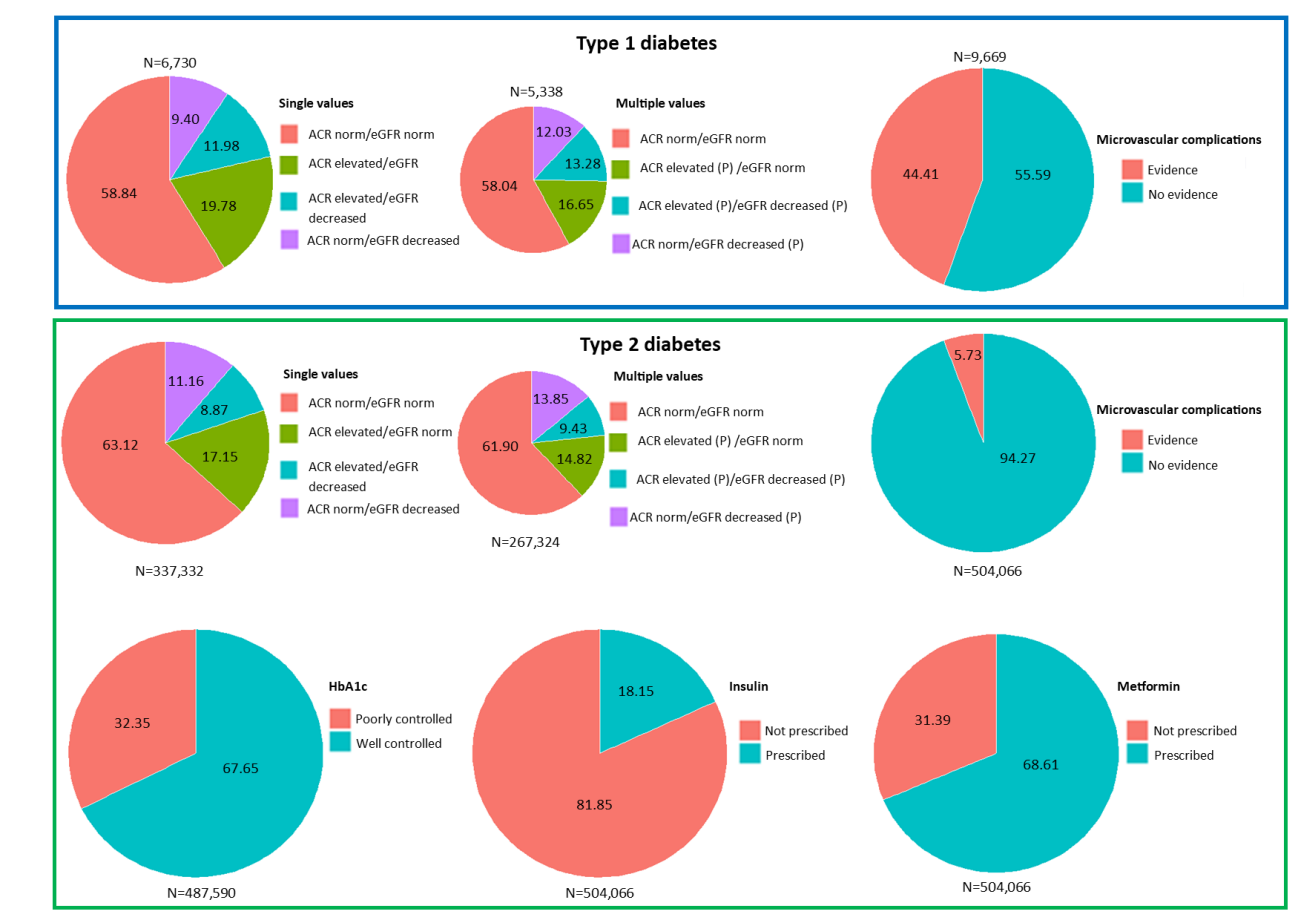
Percentage of people with and without diabetic complications in type 1 and type 2 cohorts. The size of the pie chart is proportional to the overall cohort size. Numbers within the pie chart indicate the percentage. ACR, albumin to creatinine ratio; eGFR, estimated glomerular filtration rate; HbA1c, glycated haemoglobin.

In the cohort of people with T1DM, microvascular complications were common. People with microvascular complications were slightly older than those without (figure 1). Of the 6730 people with measures of ACR and eGFR in the baseline period, 19.78% had elevated ACR with normal eGFR, 11.98% had elevated ACR and decreased eGFR and 9.40% had normal levels of ACR with decreased eGFR. A total of 5338 people with T1DM had at least two tests for both ACR and eGFR, 58.04% had normal levels of both ACR and eGFR. Individual baseline characteristics, relative to evidence of diabetes complications and status for the T1DM cohort, can be found in online supplemental tables 1–3. Overall, 17.11% (95% CI 16.36% to 17.86%) were diagnosed with intestinal/pancreatic fibrosis; skeletal fibrosis was the second most common fibrotic condition, affecting 17.03% (95% CI 16.28% to 17.78%) of the cohort (table 1). The crude and fully adjusted RRR of developing fibrotic multimorbidity was significantly higher in people with T1DM with the explored exposures, compared with the people without evidence (table 2, online supplemental table 4).

**Table 1:** Prevalence of fibrotic conditions (as of the index date) in type 1 and type 2 diabetes mellitus (T1DM/T2DM) cohorts

	Point prevalence of fibrotic conditions % (95% CI)	
	**T1DM cohort**	**T2DM cohort**
Atherosclerosis	3.08 (2.74 to 3.43)	4.24 (4.18 to 4.29)
Biliary	2.44 (2.13 to 2.75)	3.69 (3.64 to 3.75)
Blood vessel	1.47 (1.23 to 1.71)	2.25 (2.21 to 2.29)
Cardiomyopathy	14.39 (13.39 to 15.09)	18.13 (18.03 to 18.24)
Integumentary	5.08 (4.64 to 5.52)	5.14 (5.08 to 5.20)
Intest/panc	17.11 (16.36 to 17.86)	26.36 (26.24 to 26.48)
Liver	3.98 (3.59 to 4.37)	6.44 (6.38 to 6.51)
Reproductive	2.39 (1.93 to 2.85)	2.40 (2.34 to 2.46)
Lung	5.00 (4.56 to 5.43)	12.08 (11.99 to 12.17)
Skeletal	17.03 (16.28 to 17.78)	26.82 (26.70 to 26.94)
Systemic	1.58 (1.33 to 1.83)	1.94 (1.90 to 1.98)
Urinary	4.09 (3.69 to 4.48)	4.09 (4.03 to 4.14)
Valve	3.01 (2.67 to 3.35)	6.75 (6.68 to 6.82)

**Table 2:** Relative risk ratio (RRR) of being diagnosed with one or more fibrotic conditions comparing the exposed and non-exposed groups.

	Type 1 diabetes mellitus		Type 2 diabetes mellitus	
	**Adjusted RRR (95% CI)**	**P value**	**Adjusted RRR (95% CI)**	**P value**
Exposure: ACR elevated/eGFR norm				
One fibrotic condition	1.25 (1.07 to 1.45)	0.005	1.12 (1.10 to 1.15)	<0.001*
More than one fibrotic condition	1.51 (1.27 to 1.80)	<0.001*	1.42 (1.38 to 1.45)	<0.001*
Exposure: ACR elevated/eGFR decreased				
One fibrotic condition	1.72 (1.38 to 2.13)	<0.001*	1.43 (1.38 to 1.47)	<0.001*
More than one fibrotic condition	3.53 (2.86 to 4.34)	<0.001*	2.77 (2.68 to 2.86)	<0.001*
Exposure: ACR norm/eGFR decreased				
One fibrotic condition	1.23 (0.98 to 1.54)	0.073	1.21 (1.17 to 1.24)	<0.001*
More than one fibrotic condition	2.03 (1.62 to 2.54)	<0.001*	1.74 (1.68 to 1.79)	<0.001*
Exposure: persistent ACR elevated/eGFR norm				
One fibrotic condition	1.20 (1.00 to 1.44)	0.052	1.14 (1.11 to 1.17)	<0.001*
More than one fibrotic condition	1.47 (1.19 to 1.81)	<0.001*	1.42 (1.38 to 1.47)	<0.001*
Exposure: persistent ACR elevated/persistent eGFR decreased				
One fibrotic condition	1.79 (1.41 to 2.26)	<0.001*	1.48 (1.43 to 1.54)	<0.001*
More than one fibrotic condition	4.16 (3.31 to 5.23)	<0.001*	2.92 (2.82 to 3.02)	<0.001*
Exposure: ACR norm /persistent eGFR decreased				
One fibrotic condition	1.43 (1.14 to 1.81)	0.002*	1.25 (1.21 to 1.29)	<0.001*
More than one fibrotic condition	2.43 (1.92 to 3.07)	<0.001*	1.86 (1.80 to 1.92)	<0.001*
Exposure: Microvascular complications				
One fibrotic condition	1.14 (1.03 to 1.26)	0.011	1.40 (1.36 to 1.45)	<0.001*
More than one fibrotic condition	1.26 (1.13 to 1.41)	<0.001*	2.28 (2.21 to 2.36)	<0.001*
Exposure: Elevated HbA_1c_				
One fibrotic condition			1.07 (1.06 to 1.09)	<0.001*
More than one fibrotic condition			1.18 (1.16 to 1.20)	<0.001*
Exposure: Insulin prescription				
One fibrotic condition			1.51 (1.48 to 1.54)	<0.001*
More than one fibrotic condition			2.33 (2.28 to 2.37)	<0.001*
Exposure: Metformin prescription				
One fibrotic condition			0.95 (0.93 to 0.96)	<0.001*
More than one fibrotic condition			0.79 (0.78 to 0.81)	<0.001*

The fully adjusted model was adjusted for diabetic complications, age, sex, deprivation, smoking status and number of fibrotic conditions at baseline and hypertension severity.

* indicates a statistically significant p-value.

ACR, albumin to creatinine ratio; eGFR, estimated glomerular filtration rate; HbA_1c_, glycated haemoglobin.

Of the 337 332 people with T2DM and a measurement of both ACR and eGFR, 8.87% had elevated ACR and decreased eGFR and 11.16% had normal ACR with decreased eGFR. People with decreased eGFR (irrespective of ACR) were on average 13 years older than those with normal levels of ACR and eGFR. Of the 267 324 people with T2DM and multiple measurements for both ACR and eGFR, 14.82% had persistently elevated ACR with normal eGFR, 9.43% had persistently elevated ACR and persistently decreased eGFR and 13.85% had normal ACR with persistently decreased eGFR. People with persistently decreased eGFR (irrespective of ACR) were on average 12 years older than those with normal ACR and eGFR. Of the 487 590 people who had at least one recorded HbA_1c_ measurement in the 2 years prior to the index date, 32.35% had glucose above target. Of the 504 066 people with T2DM, 5.73% had evidence of microvascular complications. People with microvascular complications were on average 12 years older than those without. 18.15% of people with T2DM were prescribed insulin and 31.39% were prescribed metformin. Baseline characteristics, relative to evidence of diabetic complications and status in the T2DM cohort, can be found in online supplemental tables 5–10. The most prevalent fibrotic condition was skeletal fibrosis, affecting 26.82% (95% CI 26.70% to 26.94%) of the cohort, intestinal/pancreatic fibrosis was found in the records of 26.36% (95% CI 26.24% to 26.48%) of people (table 1). The fully adjusted RRR of being diagnosed with single and multiple fibrotic conditions was significantly higher in people with all explored diabetic complications and definitions of diabetes status (except metformin), compared with those without (table 2, online supplemental table 4).

### Type 1 diabetes mellitus

#### Exposure: all microvascular complications

**Figure 2:**
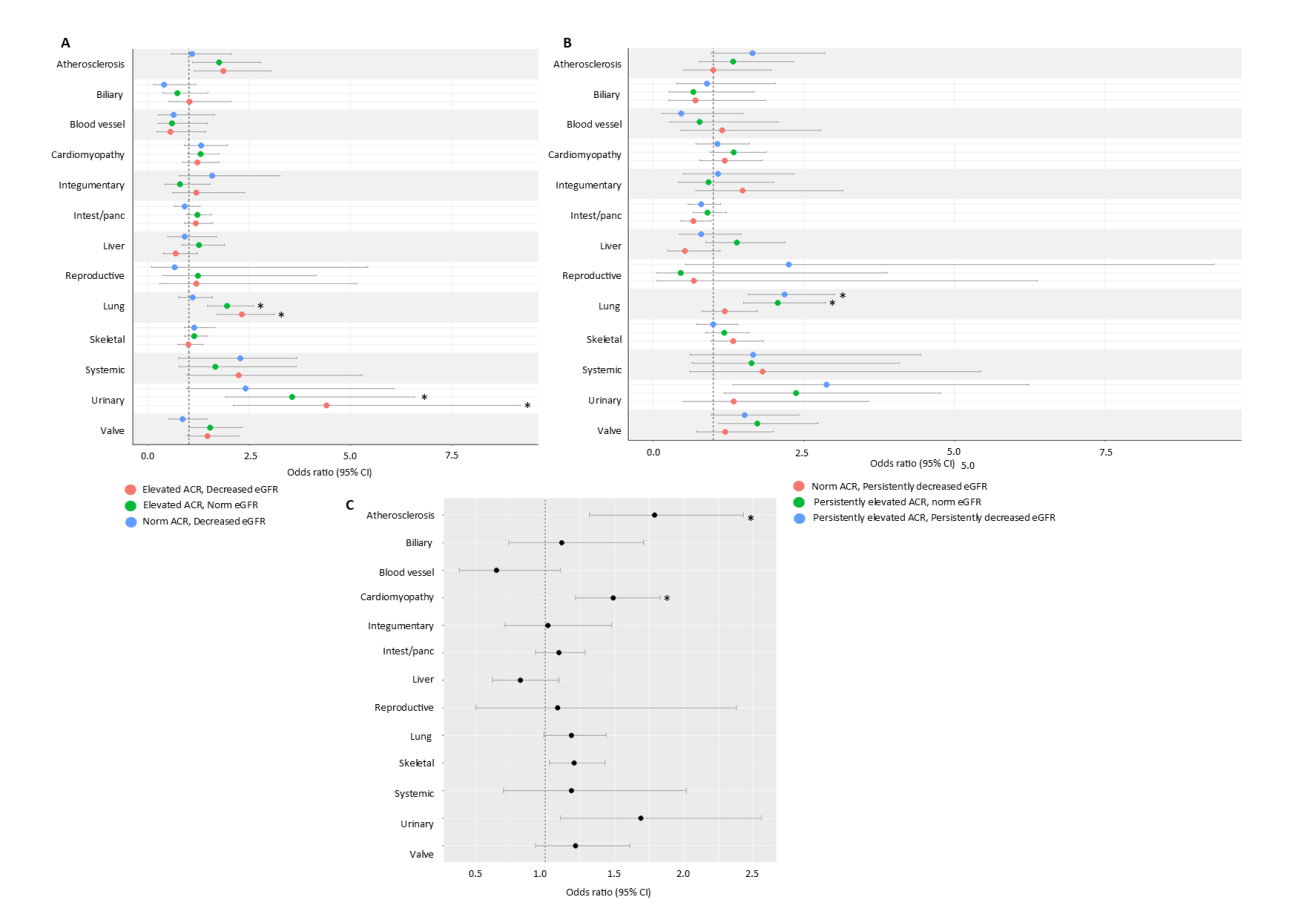
Association of diabetic complications and fibrotic conditions in a type 1 diabetic cohort. (**A**) Microvascular complications, (**B**) Single measures of ACR and eGFR, (**C**) Multiple measures of ACR and eGFR. ACR, albumin to creatinine ratio; eGFR, estimated glomerular filtration rate.

Prevalence of fibrotic conditions did not differ with respect to microvascular complications (online supplemental table 11). People with microvascular complications were 1.79 times more likely to be diagnosed with atherosclerosis and 1.49 times more likely to be diagnosed with cardiomyopathy (figure 2A, online supplemental table 12).

#### Exposure: ACR/eGFR measurements

Atherosclerosis, blood vessel fibrosis, cardiomyopathy, lung fibrosis, systemic fibrosis, urinary fibrosis and valve fibrosis were more common in those with decreased eGFR when compared with those with normal ACR and eGFR (online supplemental table 13). Increased ACR (irrespective of eGFR) was associated with lung fibrosis (figure 2B, online supplemental table 14). Compared with normal levels of ACR and eGFR, all subgroups of ACR/eGFR were associated with urinary fibrosis.

#### Exposure: persistently raised ACR/eGFR measurements

Prevalence of fibrotic conditions differed with respect to multiple measures of ACR/eGFR (online supplemental table 15). People with persistently elevated ACR were at least two times more likely to be diagnosed with lung fibrosis, compared with those with normal levels of ACR and eGFR (figure 2C, online supplemental table 16).

#### Sensitivity analysis

Findings were unchanged when additionally adjusting for CCI, OCS, diabetes duration and BMI, respectively (online supplemental tables 17–28).

### Type 2 diabetes mellitus

#### Exposure: all microvascular complications

**Figure 3:**
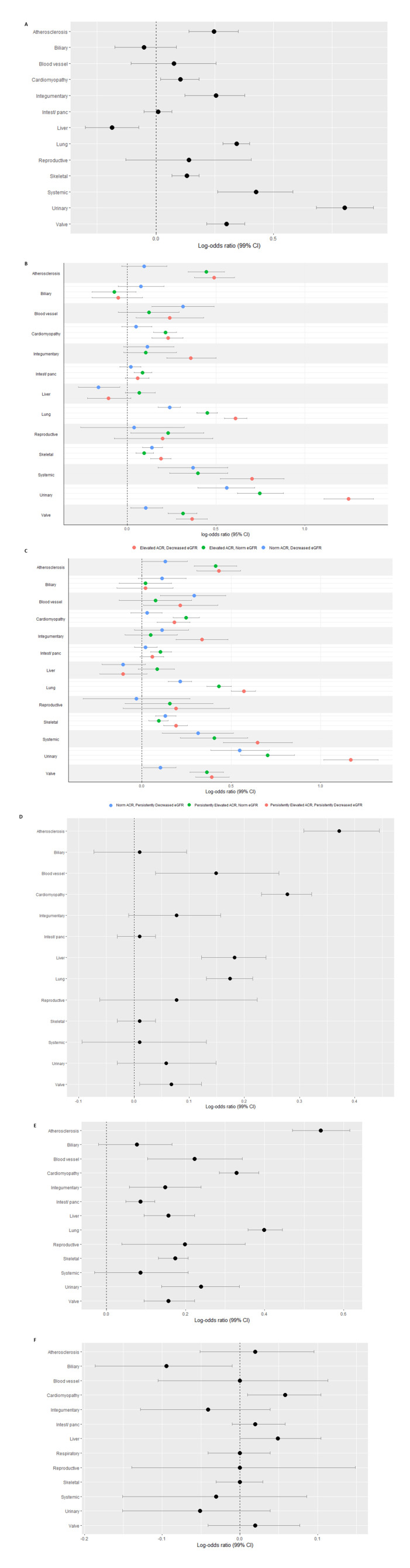
Association of diabetic complications and fibrotic conditions in a type 2 diabetic cohort. (**A**) Microvascular complications, (**B**) single measures of ACR and eGFR, (**C**) multiple measures of ACR and eGFR, (**D**) glucose above target, (**E**) insulin prescription, (**F**) metformin prescription. ACR, albumin to creatinine ratio; eGFR, estimated glomerular filtration rate.

Atherosclerosis, integumentary fibrosis, lung fibrosis, systemic fibrosis, urinary fibrosis and valve fibrosis were more common in people with microvascular complications (online supplemental table 29). An inverse association between microvascular complications and liver fibrosis was observed in the fully adjusted model (figure 3A**,** online supplemental table 30). Microvascular complications were associated with most fibrotic conditions.

#### Exposure: ACR/eGFR measurements

Atherosclerosis, blood vessel fibrosis, lung fibrosis and systemic fibrosis were more prevalent in those with decreased eGFR (online supplemental table 31). Integumentary fibrosis and valve fibrosis was more common in those with elevated ACR and decreased eGFR. All three exposures pertaining to increased ACR or decreased eGFR were associated with: lung fibrosis, skeletal fibrosis, systemic fibrosis, urinary fibrosis and valve fibrosis (figure 3B, online supplemental table 32).

#### Exposure: persistently raised ACR/eGFR measurements

Integumentary fibrosis, lung, systemic and valve fibrosis affected a greater percentage of people with persistently increased ACR and persistently decreased eGFR compared with those with normal levels (online supplemental table 33). All three exposures were associated with atherosclerosis, lung, skeletal, systemic, urinary and valve fibrosis (compared with people with normal levels of ACR and eGFR) (figure 3C, online supplemental table 34). Persistently raised ACR (irrespective of eGFR) was associated with cardiomyopathy, while persistently raised ACR with persistently decreased eGFR was associated with integumentary fibrosis.

#### Exposure: glucose above target

With respect to HBA_1c_, the proportion of people with fibrotic conditions did not differ (online supplemental table 35). The results of fully adjusted logistic regression analyses found that higher HbA_1c_ was associated with atherosclerosis, blood vessel fibrosis, cardiomyopathy, liver fibrosis, lung fibrosis and valve fibrosis (figure 3D, online supplemental table 36).

#### Exposure: insulin prescription

The prevalence of fibrotic conditions did not differ with respect to insulin prescription (online supplemental table 37). Insulin prescription was associated with all fibrotic conditions excluding biliary fibrosis and urinary fibrosis (figure 3E, online supplemental table 38).

#### Exposure: metformin prescription

Prevalence of fibrotic conditions did not differ with regards to metformin prescription (online supplemental table 39). Crude logistic regression analyses indicated a potential protective effect of metformin against the diagnosis of fibrotic conditions; however, this did not hold in fully adjusted analyses. Prescription of metformin was associated with cardiomyopathy when all covariates were accounted for (OR 1.06, 99% CI 1.01 to 1.11). No other associations (protective or otherwise) were observed when comparing the data of those prescribed and not prescribed metformin (figure 3F, online supplemental table 40).

#### Sensitivity analyses

Microvascular complications were not protective of liver fibrosis when including adjustment for CCI (online supplemental table 41) and elevated ACR with normal eGFR was no longer associated with reproductive fibrosis (online supplemental table 42), findings were otherwise unchanged (online supplemental tables 42–44). Insulin prescription was not associated with reproductive fibrosis, adjusting for CCI (online supplemental table 45). Results did not change with respect to metformin prescription (online supplemental table 46). Findings were unchanged when additionally including OCS and diabetes duration (respectively) in logistic regression models (online supplemental tables 47–59). When including BMI in models, normal levels of ACR with decreased eGFR were no longer associated with valve fibrosis (online supplemental table 60). Normal ACR with persistently decreased eGFR was not associated with atherosclerosis or valve fibrosis when including BMI (online supplemental table 61). When including BMI, glucose above target was not associated with blood vessel fibrosis (online supplemental table 62), all other findings were consistent (online supplemental tables 63–64).

## Discussion

Using observational data, we investigated whether diabetes status and diabetic complications were associated with fibrotic diseases. In T1DM, albuminuria was found to be associated with lung fibrosis, while microvascular complications were associated with atherosclerosis and cardiomyopathy. In those with T2DM, most diabetes complications were associated with future diagnosis of fibrotic conditions. This study supports the hypothesis (specifically in people with T2DM) that more significant hyperglycaemia, attenuated response to hypoglycaemic therapies and greater propensity to complications are associated with fibrosis.

Both hyperglycaemia and hypoglycaemia upregulate inflammation, lead to increased oxidative stress and disrupted DNA repair mechanisms and can lead to the activation of transforming growth factor beta signals.^24^,^28^ These mechanisms are also implicated in fibrosis^29^,^32^; therefore, it is important to understand whether diabetes mellitus is associated with fibrotic conditions and whether this is due to shared biological mechanisms.

Albuminuria is a marker of both renal impairment and endothelial dysfunction and can indicate systemic vascular damage.^33^ In a COPD population, albuminuria has been shown to be associated with COPD severity.^34^ It has also been shown that increased albuminuria is associated with a higher risk of developing lung cancer.^35^ It is believed that here we have been the first to establish an association between albuminuria (in those with T1DM) and lung fibrosis; this too could be a result of vascular damage. It has been postulated that vascular abnormalities are both causal and consequential in idiopathic pulmonary fibrosis (IPF) pathobiology.^36^ However, previous genetic studies have found no evidence of a direct causal relationship between diabetes (including exposures of HbA_1c_, fasting insulin level and BMI) and IPF.^37^ Thus, it is not currently clear whether endothelial dysfunction and shortened telomeres represent shared causal mechanisms of diabetes with more significant and treatment-resistant hyperglycaemia, and further studies are required to determine the nature of these associations.^38 39^

Furthermore, T2DM is more common in older people and is associated with shorter telomere length,^40 41^ and these mechanisms are shared in people with fibrotic disease.^42^ ^43^ The association between fibrotic diseases and both hyperglycaemia and microvascular complications in T2DM requires further research to understand how these findings interact with the well-understood micro- and macrovascular complications related to hyperglycaemia and insulin resistance.

Regular monitoring and screening of diabetes management and complication status is important for the prevention, identification and optimisation of diabetes complications. Completing the care processes for people with diabetes has been associated with reduced all-cause mortality.^44^ We have shown that diabetes complications are associated with fibrotic conditions. As such, we believe it may be important to further explore the possibility of screening patients with T2DM and diabetic complications for fibrotic conditions, to aid earlier detection of fibrotic conditions and provide more specific patient care given new knowledge. Currently, people with diabetes are screened for cardiovascular diseases, this screening process could present an opportunity for expansion to include fibrotic conditions. Many important questions must be first explored, for instance, which screening processes should be employed, the cost effectiveness as well as the feasibility of altering current screening protocols, the regularity of screening and the establishment of required baseline characteristics to qualify for screening (eg, age, presence of diabetes complications). This would be a significant undertaking requiring the expertise of multiple disciplines.

More research is required to fully understand what underpins this observed association between albuminuria (possibly due to vascular leak) and pulmonary fibrosis and potential implications for clinical practice. It is important to understand whether observed associations are due to shared pathways and risk factors, as this will contribute towards better drug targets (including drug repurposing), potentially for multiple diseases. It is important to understand which shared risk factors are associated with the development of diabetes and fibrotic conditions to better understand this comorbid relationship. Also, it would be useful to further understand whether in people with diabetes and diabetes complications, there is a greater risk of other clinical features, including cardiovascular diseases as well as mortality.

### Strengths and limitations

The main strength of this analysis is that we have used routinely collected EHRs to understand the association between diabetes status and complications with fibrotic conditions. These records are commonly used in research as they capture a broad amount of information regarding health and treatment. However, it is also important to acknowledge a limitation of these data. They are entered (in primary care) by the treating clinician; in some instances, key details and information may not be recorded using clinical codes. Another limitation of using data regarding prescribed medications (in this instance insulin and metformin) is that this data relates to the prescription of the drug and not the dispensation or adherence. The results of tests were used to identify exposures in this analysis; however, sometimes a clinician may enter that a test has been performed but the result of the test may not be recorded. This would mean that the exposure could not be defined in this instance. Therefore, our analysis of these exposures only included the data of people with a recorded result. Here, we have conducted a study primarily analysing the association between a diabetes-related exposure and a fibrotic disease. However, we acknowledge that the date of entry of a fibrotic code does not equate to when a disease first manifested and, therefore, this adds complexity when trying to disentangle the direction of effect. However, in all analyses where a person had the fibrotic condition prior to the index date, we ensured they were removed from the respective analysis. A strength of this analysis is that we have also included further investigation of covariates which may explain the observed associations in sensitivity analyses. This extra work helps to contextualise and support the main study findings. With regard to the main covariates used in analysis, we believe these were well posed as in the fully adjusted models, the effect sizes were smaller; however, residual confounding (such as physical activity, dietary patterns and medication adherence) may explain some associations. Insulin-treated T2DM is a potentially heterogeneous and confounded group and, therefore, caution should be applied when interpreting results of this subgroup. It is important to acknowledge that when conducting logistic regression analysis, only people with a minimum of 3 years of follow-up (therefore must be alive and continuously enrolled at their GP practice) were included in this analysis. As a result, immortal time bias may have led to an underestimation of associations, as high-risk patient may have died within this time and therefore would not contribute data.

## Conclusions

In people with T2DM, complications were associated with a greater relative risk of fibrotic conditions. The specific associations found were between albuminuria and lung fibrosis and microvascular complications and atherosclerosis/cardiomyopathy, respectively. Also, associations in people with T2DM, between diabetic complications, diabetes status and the development of fibrotic conditions were consistently observed. These findings may suggest that complicated T2DM may have a fibrotic component.

## Supplementary material

10.1136/bmjopen-2025-103426 online supplemental file 1

## Data Availability

Data may be obtained from a third party and are not publicly available.
