# Supplementary material for: Association between hyperglycaemia, diabetes complications and development of fibrotic conditions among people living with type 1 and type 2 diabetes in England: a retrospective cohort study using UK Clinical Resource Datalink Aurum and Hospital Episode Statistics
Source: BMJ Open. 2025 Oct 27;15(10):e103426. doi: 10.1136/bmjopen-2025-103426 (PMC12570948; doi:10.1136/bmjopen-2025-103426)
Supplement: online supplemental file 1 [file bmjopen-15-10-s001.docx]

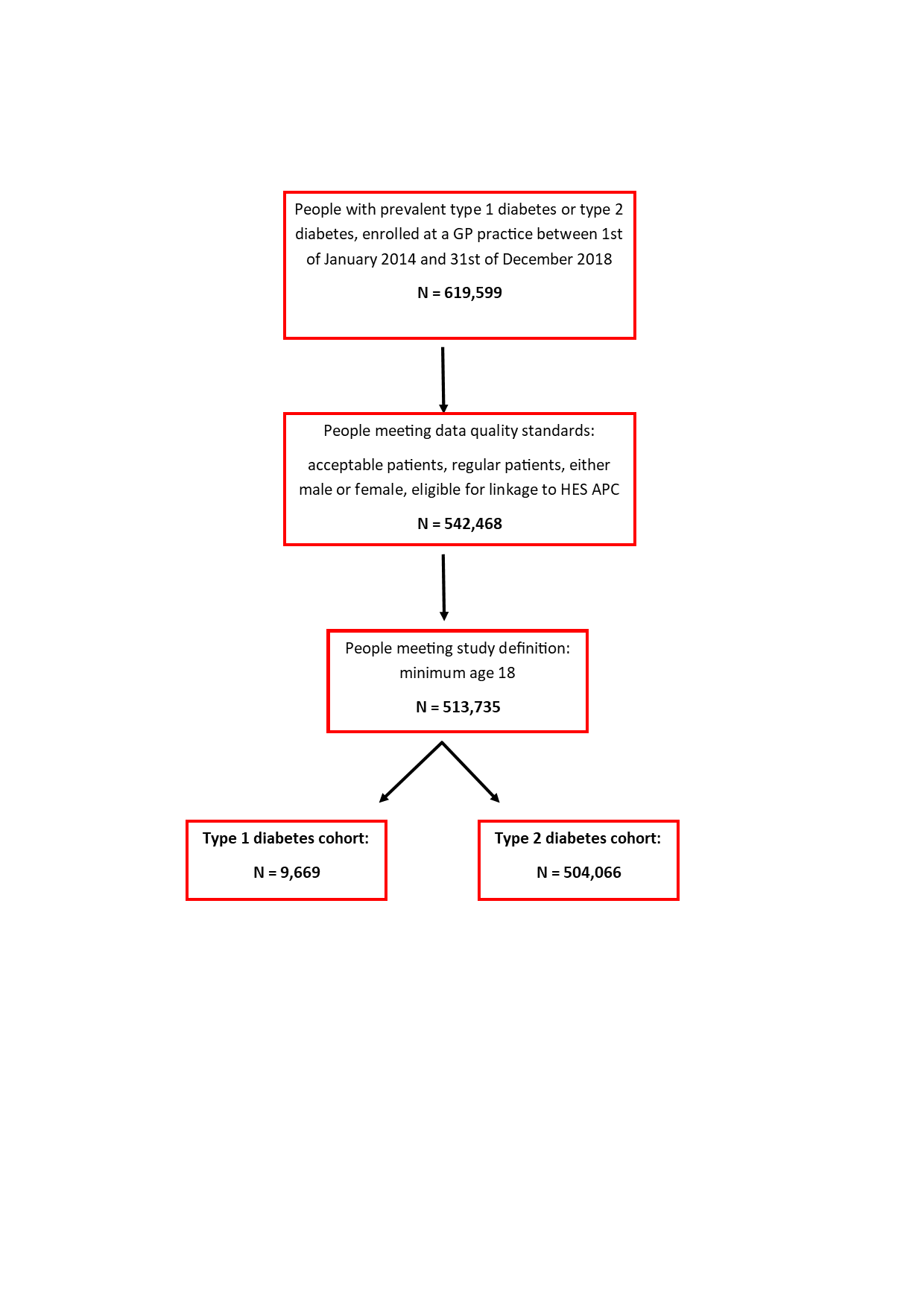


Figure 1 | Diagram demonstrating the application of study inclusion criteria

s


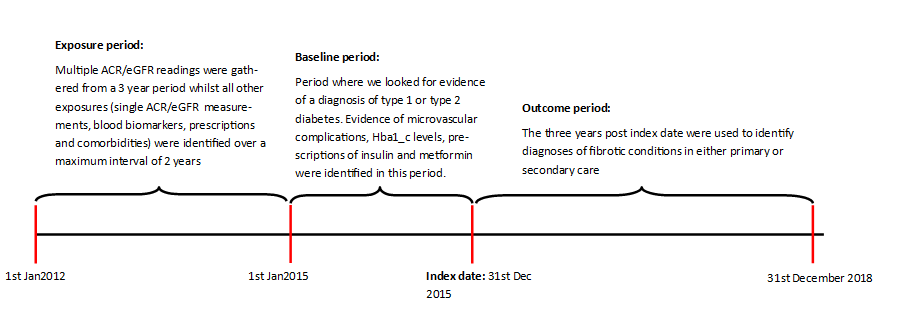


Figure 2 | Diagram demonstrating the study period that was investigate and the periods of time used to define exposures, covariates and outcomes

Table 1 | Baseline characteristics of people with type 1 diabetes, the exposure was evidence of microvascular complications. Microvascular complications were defined as binary composite exposure was defined utilising codes for neuropathy, retinopathy, and CKD (defined as persistently increased ACR and persistently decreased eGFR). IMD: Index of multiple deprivation, FMM: fibrotic multimorbidity (two or more fibrotic conditions).

|  | **Whole cohort** |  | **No evidence of microvascular complications** |  | **Evidence of microvascular complications** |
| --- | --- | --- | --- | --- | --- |
|  |  |  |  |  |  |
| Size of cohort (%) | 9,669 |  | 5,375 (55.59) |  | 4,294 (44.41) |
| Median age (IQR) | 63 (52 – 73) |  | 61 (51 – 72) |  | 64 (54 – 74) |
| Sex (% female) | 43.19 |  | 44.41 |  | 41.66 |
|  |  |  |  |  |  |
| **Smoking status (%)** |  |  |  |  |  |
| Never | 48.75 |  | 47.96 |  | 49.74 |
| Ex-smoker | 31.01 |  | 30.44 |  | 31.72 |
| Current smoker | 20.24 |  | 21.60 |  | 18.54 |
|  |  |  |  |  |  |
| **IMD (%)** |  |  |  |  |  |
| 1 (Least deprived) | 17.19 |  | 17.85 |  | 16.37 |
| 2 | 18.37 |  | 18.65 |  | 18.03 |
| 3 | 19.84 |  | 20.12 |  | 19.50 |
| 4 | 22.22 |  | 21.14 |  | 23.58 |
| 5 (Most deprived) | 22.38 |  | 22.26 |  | 22.53 |
|  |  |  |  |  |  |
| >=1 fibrotic condition, %, (95% CI) | 46.14  (45.15 – 47.13) |  | 42.87  (41.55 – 44.19) |  | 50.23  (48.73 – 51.73) |
| FMM (%), (95% CI) | 21.01  (20.20 – 21.82) |  | 18.60  (17.56 – 19.64) |  | 24.01  (22.73 – 25.29) |

Table 2 | Baseline characteristics of those with type 1 diabetes, the exposure was measures of albumin to creatinine ratio (ACR) and glomerular filtration rate (eGFR). Elevated ACR and eGFR was defined as ACR ≥3 mg/mmol, eGFR <60 mL/min/1.73 m^2^ respectively. *The whole cohort consists of people with measures of both ACR and eGFR present in the 2-year period prior to the study period. IMD: Index of multiple deprivation, FMM: fibrotic multimorbidity (two or more fibrotic conditions).

|  | **Whole cohort *** |  | **ACR norm / eGFR norm** |  | **ACR elevated/ eGFR norm** |  | **ACR elevated/ eGFR decreased** |  | **ACR norm / eGFR decreased** |
| --- | --- | --- | --- | --- | --- | --- | --- | --- | --- |
|  |  |  |  |  |  |  |  |  |  |
| Size of cohort (%) | 6,730 |  | 3,960 (58.84) |  | 1,331 (19.78) |  | 806 (11.98) |  | 633 (9.40) |
| Median age (IQR) | 64 (54 – 74) |  | 60 (51 – 70) |  | 63 (53 – 72) |  | 75 (67 – 81) |  | 76 (69 – 82) |
| Sex (% female) | 42.48 |  | 40.91 |  | 41.25 |  | 42.31 |  | 55.13 |
|  |  |  |  |  |  |  |  |  |  |
| **Smoking status (%)** |  |  |  |  |  |  |  |  |  |
| Never | 48.50 |  | 48.31 |  | 46.58 |  | 50.50 |  | 51.18 |
| Ex-smoker | 32.41 |  | 31.62 |  | 29.23 |  | 36.72 |  | 38.55 |
| Current smoker | 19.09 |  | 20.08 |  | 24.19 |  | 12.78 |  | 10.27 |
|  |  |  |  |  |  |  |  |  |  |
| **IMD (%)** |  |  |  |  |  |  |  |  |  |
| 1 (Least deprived) | 17.19 |  | 18.96 |  | 13.00 |  | 16.02 |  | 16.46 |
| 2 | 18.83 |  | 19.21 |  | 16.90 |  | 19.38 |  | 19.78 |
| 3 | 19.57 |  | 19.39 |  | 20.59 |  | 19.01 |  | 19.30 |
| 4 | 21.82 |  | 20.90 |  | 22.61 |  | 22.61 |  | 24.84 |
| 5 (Most deprived) | 22.59 |  | 21.54 |  | 26.90 |  | 22.98 |  | 19.62 |
|  |  |  |  |  |  |  |  |  |  |
| >=1 fibrotic condition, %, (95% CI) | 47.10  (45.91 – 48.29) |  | 38.48  (36.96 – 40.00) |  | 49.06  (46.37 – 51.75) |  | 73.33  (70.28 – 76.38) |  | 63.51  (59.76 – 37.26) |
| FMM (%), (95% CI) | 21.86  (20.87 – 22.85) |  | 14.72  (13.62 – 15.82) |  | 22.01  (19.78 – 24.24) |  | 45.66  (42.22 – 49.10) |  | 35.86  (32.12 – 39.60) |

Table 3 |Baseline characteristics of those with type 1 diabetes, the exposure was multiple measures of albumin to creatinine ratio (ACR) and glomerular filtration rate (eGFR). Persistently elevated ACR and persistently elevated eGFR was defined as at least two readings of ACR/eGFR in the 3 years prior to index date of ACR ≥3 mg/mmol, eGFR <60 mL/min/1.73 m^2^ respectively. *The whole cohort consists of people with multiple measures of both ACR and eGFR present in the 3-year period prior to the study period. IMD: Index of multiple deprivation, FMM: fibrotic multimorbidity (two or more fibrotic conditions).

|  | **Whole cohort *** | **ACR norm / eGFR norm** | **Persistent ACR elevated/ eGFR norm** | **Persistent ACR elevated/ Persistent eGFR decreased** | **ACR norm / Persistent eGFR decreased** |
| --- | --- | --- | --- | --- | --- |
|  |  |  |  |  |  |
| Size of cohort (%) | 5,338 | 3,098 (58.04) | 889 (16.65) | 709 (13.28) | 642 (12.03) |
| Median age (IQR) | 66 (55 – 75) | 61 (52 – 70) | 64 (54 – 73) | 75 (69 – 82) | 76 (69 – 82) |
| Sex (% female) | 41.83 | 39.77 | 40.04 | 40.48 | 55.76 |
|  |  |  |  |  |  |
| **Smoking status (%)** |  |  |  |  |  |
| Never | 47.86 | 47.58 | 45.22 | 49.37 | 51.25 |
| Ex-smoker | 33.72 | 32.34 | 31.50 | 38.79 | 37.85 |
| Current smoker | 18.42 | 20.08 | 23.28 | 11.85 | 10.90 |
|  |  |  |  |  |  |
| **IMD (%)** |  |  |  |  |  |
| 1 (Least deprived) | 17.42 | 18.58 | 12.26 | 16.81 | 19.66 |
| 2 | 19.04 | 19.42 | 18.00 | 18.93 | 18.72 |
| 3 | 19.67 | 19.68 | 19.91 | 20.62 | 18.25 |
| 4 | 21.57 | 20.91 | 21.15 | 23.59 | 23.09 |
| 5 (Most deprived) | 22.30 | 21.40 | 28.68 | 20.06 | 20.28 |
|  |  |  |  |  |  |
| >=1 fibrotic condition, %, (95% CI) | 48.16  (46.82 – 49.50) | 48.22  (46.46 – 49.98) | 48.82  (45.53 – 52.11) | 74.47  (71.26 – 77.68) | 66.20  (62.54 - 69386) |
| FMM (%), (95% CI) | 22.71  (21.59 – 23.83) | 14.40  (13.16 – 15.64) | 21.82  (19.10 – 24.54) | 47.39  (43.71 – 51.07) | 36.76  (33.03 – 40.49) |

Table 4 | Crude Relative risk ratio (RRR) of being diagnosed with one or more fibrotic conditions comparing the exposed and non-exposed groups.

|  | **Type 1 DM** | **Type 2 DM** | |
| --- | --- | --- | --- |
|  | **Crude RRR (95% CI)** |  | **Crude RRR**  **(95% CI)** |
|  | Exposure: Microvascular complications | | |
| **One fibrotic condition** | 1.24  (1.13 – 1.37) |  | *2.08*  *(2.01 – 2.15)* |
| **More than one fibrotic condition** | 1.48  (1.34 – 1.64) |  | 4.34  (4.21 – 4.48) |
|  | Exposure: ACR elevated / eGFR norm | | |
| **One fibrotic condition** | 1.37  (1.19 – 1.59) |  | 1.21  (1.19 – 1.24) |
| **More than one fibrotic condition** | 1.81  (1.53 – 2.13) |  | 1.60  (1.56 – 1.64) |
|  | Exposure: ACR elevated / eGFR decreased | | |
| **One fibrotic condition** | 2.69  (2.19 – 3.29) |  | 2.13  (2.06 – 2.19) |
| **More than one fibrotic condition** | 7.15  (5.91 – 8.66) |  | 5.24  (5.08 – 5.40) |
|  | Exposure: ACR norm / eGFR decreased | | |
| **One fibrotic condition** | 1.96  (1.59 – 2.42) |  | 1.76  (1.72 – 1.81) |
| **More than one fibrotic condition** | 4.11  (3.35 – 5.04) |  | 3.18  (3.10 – 3.27) |
|  | Exposure: persistent ACR elevated / eGFR norm | | |
| **One fibrotic condition** | 1.37  (1.15 – 1.63) |  | 1.23  (1.20 – 1.26) |
| **More than one fibrotic condition** | 1.83  (1.50 – 2.23) |  | 1.60  (1.56 – 1.26) |
|  | Exposure: persistent ACR elevated / persistent eGFR decreased | | |
| **One fibrotic condition** | 2.75  (2.21 – 3.43) |  | 2.15  (2.08 – 2.23) |
| **More than one fibrotic condition** | 7.97  (6.47 – 9.81) |  | 5.28  (5.11 – 5.46) |
|  | Exposure: ACR norm / persistent eGFR decreased | | |
| **One fibrotic condition** | 2.26  (1.83 – 2.79) |  | 1.77  (1.72 – 1.82) |
| **More than one fibrotic condition** | 4.67  (3.78 – 5.76) |  | 3.24  (3.15 – 3.33) |
|  | *Exposure: Glucose above target* | | |
| **One fibrotic condition** |  |  | 0.94  (0.92 – 0.95) |
| **More than one fibrotic condition** |  |  | 0.94  (0.92 – 0.96) |
|  | *Exposure: Insulin prescription* | | |
| **One fibrotic condition** |  |  | 1.45  (1.42 – 1.47) |
| **More than one fibrotic condition** |  |  | 2.16  (2.12 – 2.20) |
|  | *Exposure: Metformin prescription* | | |
| **One fibrotic condition** |  |  | 0.89  (0.88 – 0.90) |
| **More than one fibrotic condition** |  |  | 0.72  (0.70 – 0.73) |

Table 5 | Baseline characteristics of people with type 2 diabetes, the exposure was evidence of microvascular complications. Microvascular complications were defined as binary composite exposure was defined utilising codes for neuropathy, retinopathy, and CKD (defined as persistently increased ACR and persistently decreased eGFR). IMD: Index of multiple deprivation, FMM: fibrotic multimorbidity (two or more fibrotic conditions).

|  | **Whole cohort** | **No evidence of MV** | **Evidence of MV** |
| --- | --- | --- | --- |
|  |  |  |  |
| Size of cohort (%) | 504,066 | 475,851 (94.27) | 28,931 (5.73) |
| Median age (IQR) | 68 (58 – 77) | 67 (57 – 76) | 79 (72 – 85) |
| Sex (% female) | 44.30 | 44.25 | 45.07 |
| **Smoking status (%)** |  |  |  |
| Never | 47.75 | 47.82 | 46.61 |
| Ex-smoker | 33.46 | 33.10 | 39.45 |
| Current smoker | 18.78 | 19.08 | 13.95 |
| **IMD (%)** |  |  |  |
| 1 (Least deprived) | 16.97 | 16.95 | 17.31 |
| 2 | 18.88 | 18.88 | 18.93 |
| 3 | 19.40 | 19.40 | 19.44 |
| 4 | 21.77 | 21.78 | 21.61 |
| 5 (Most deprived) | 22.98 | 23.00 | 22.71 |
| **Fibrotic conditions** |  |  |  |
| >=1 fibrotic condition, %, (95% CI) | 59.31  (59.17 – 59.45) | 57.87  (57.73 – 58.01) | 84.54  (84.12 – 84.96) |
| FMM, %, (95% CI) | 33.06  (32.93 – 33.19) | 31.45  (31.32 – 31.58) | 62.45  (61.89 – 63.01) |

Table 6 | Baseline characteristics of those with type 2 diabetes, the exposure was measures of albumin to creatinine ratio (ACR) and glomerular filtration rate (eGFR). Elevated ACR and eGFR was defined as ACR ≥3 mg/mmol, eGFR <60 mL/min/1.73 m^2^ respectively. *The whole cohort consists of people with measures of both ACR and eGFR present in the 2-year period prior to the study period. IMD: Index of multiple deprivation, FMM: fibrotic multimorbidity (two or more fibrotic conditions).

|  | **Whole cohort *** | **ACR norm / eGFR norm** | **ACR elevated/ eGFR norm** | **ACR elevated/ eGFR decreased** | **ACR norm / eGFR decreased** |
| --- | --- | --- | --- | --- | --- |
|  |  |  |  |  |  |
| Size of cohort (%) | 337,332 | 212,910 (63.12) | 57,848 (17.15) | 28,931 (8.87) | 37,643 (11.16) |
| Median age (IQR) | 69 (59 - 77) | 66 (57 – 74) | 67 (58 – 76) | 79 (72 – 85) | 79 (73 – 84) |
| Sex (% female) | 43.13 | 41.27 | 41.52 | 45.07 | 54.60 |
| **Smoking status (%)** |  | | | |  |
| Never | 46.97 | 47.31 | 44.62 | 46.61 | 48.97 |
| Ex-smoker | 34.91 | 34.03 | 33.42 | 39.45 | 38.74 |
| Current smoker | 18.12 | 18.67 | 21.96 | 13.95 | 12.29 |
| **IMD (%)** |  | | | |  |
| 1 (Least deprived) | 16.78 | 16.87 | 15.56 | 17.31 | 17.73 |
| 2 | 18.82 | 18.97 | 17.51 | 18.93 | 19.85 |
| 3 | 19.14 | 19.13 | 18.70 | 19.44 | 19.60 |
| 4 | 21.74 | 21.59 | 22.83 | 21.61 | 20.95 |
| 5 (Most deprived) | 23.53 | 23.43 | 25.40 | 22.71 | 21.88 |
|  | | | | |  |
| >=1 fibrotic condition, %, (95% CI) | 61.50  (61.34 – 61.66) | 54.85  (54.64 – 55.06) | 64.48  (64.09 – 64.87) | 84.38  (83.96 – 84.80) | 76.91  (76.48 – 77.34) |
| FMM, %, (95% CI) | 34.96  (34.80 – 35.12) | 27.71  (27.52 – 27.90) | 37.78  (37.38 – 38.18) | 62.05  (61.49 – 62.61) | 50.77  (50.26 – 51.28) |

Table 7 |Baseline characteristics of those with type 2 diabetes, the exposure was multiple measures of albumin to creatinine ratio (ACR) and glomerular filtration rate (eGFR). Persistently elevated ACR and persistently elevated eGFR was defined as at least two readings of ACR/eGFR in the 3 years prior to index date of ACR ≥3 mg/mmol, eGFR <60 mL/min/1.73 m^2^ respectively. *The whole cohort consists of people with multiple measures of both ACR and eGFR present in the 3-year period prior to the study period. IMD: Index of multiple deprivation, FMM: fibrotic multimorbidity (two or more fibrotic conditions).

|  | **Whole cohort *** | **ACR norm / eGFR norm** | **Persistent ACR elevated/ eGFR norm** | **Persistent ACR elevated/ Persistent eGFR decreased** | **ACR norm / Persistent eGFR decreased** |
| --- | --- | --- | --- | --- | --- |
|  |  |  |  |  |  |
| Size of cohort (%) | 267,324 | 165,466 (61.90) | 39,618 (14.82) | 25,198 (9.43) | 37,042 (13.85) |
| Median age (IQR) | 70 (61 – 78) | 67 (58 – 74) | 68 (59 – 76) | 79 (73 – 84) | 79 (73 – 84) |
| Sex (% female) | 42.51 | 40.71 | 38.51 | 43.38 | 54.26 |
| **Smoking status (%)** |  | | | |  |
| Never | 46.66 | 47.09 | 43.30 | 45.42 | 49.17 |
| Ex-smoker | 35.78 | 34.70 | 34.69 | 40.34 | 38.67 |
| Current smoker | 17.56 | 18.21 | 22.01 | 14.24 | 12.16 |
| **IMD (%)** |  | | | |  |
| 1 (Least deprived) | 16.89 | 16.97 | 15.42 | 17.27 | 17.82 |
| 2 | 18.91 | 19.04 | 17.61 | 19.28 | 19.47 |
| 3 | 19.18 | 19.11 | 18.80 | 19.72 | 19.54 |
| 4 | 21.52 | 21.46 | 22.68 | 20.81 | 21.04 |
| 5 (Most deprived) | 23.50 | 23.42 | 25.48 | 22.92 | 22.14 |
|  | | | | |  |
| >=1 fibrotic condition, %, (95% CI) | 62.83  (62.65 – 63.01) | 55.56  (55.32 – 55.80) | 65.55  (65.08 – 66.02) | 84.77  (84.33 – 85.21) | 77.48  (62.08 – 63.06) |
| FMM, %, (95% CI) | 36.28  (36.10 – 36.46) | 28.29  (28.07 – 28.51) | 38.77  (38.29 – 39.25) | 62.57  (61.97 – 63.17) | 51.40  (50.89 – 51.91) |

Table 8 | Baseline characteristics of those with type 2 diabetes with controlled glucose and glucose above target. Poorly controlled was defined as HbA1_C >=7.5%. HbA_1c_ was defined as the most recent value recorded prior to the study index date. IMD: Index of multiple deprivation, FMM: fibrotic multimorbidity (two or more fibrotic conditions).

|  | **Whole cohort *** | **Controlled glucose** | **Glucose above target** |
| --- | --- | --- | --- |
|  |  |  |  |
| Size of cohort (%) | 487,590 | 329,850 (67.65) | 157,740 (32.35) |
| Median age (IQR) | 68 (58 – 77) | 69 (60 - 78) | 64 (55 - 73) |
| Sex (% female) | 44.34 | 45.41 | 42.12 |
| **Smoking status (%)** |  |  |  |
| Never | 47.78 | 47.80 | 47.74 |
| Ex-smoker | 33.74 | 34.41 | 32.32 |
| Current smoker | 18.48 | 17.78 | 19.94 |
| **IMD (%)** |  |  |  |
| 1 (Least deprived) | 17.11 | 17.83 | 15.59 |
| 2 | 18.98 | 19.52 | 17.85 |
| 3 | 19.44 | 19.59 | 19.12 |
| 4 | 21.68 | 21.22 | 22.64 |
| 5 (Most deprived) | 22.79 | 21.83 | 24.80 |
| **Fibrotic conditions** |  |  |  |
| >=1 fibrotic condition, %, (95% CI) | 59.77  (59.63 – 59.91) | 60.27  (60.10 – 60.44) | 58.72  (58.48 – 58.96) |
| FMM, % (95% CI) | 33.48  (33.35 – 33.61) | 33.64  (33.48 – 33.80) | 33.16  (32.93 – 33.39) |

Table 9 | Baseline characteristics of those with type 2 diabetes, the exposure was prescription of insulin in the year prior to the index date. IMD: Index of multiple deprivation, FMM: fibrotic multimorbidity (two or more fibrotic conditions).

|  | **Whole cohort** | **Not prescribed insulin** | **Prescribed insulin** |
| --- | --- | --- | --- |
|  |  |  |  |
| Size of cohort (%) | 504,066 | 412,564 (81.85) | 91,502 (18.15) |
| Median age (IQR) | 68 (58 – 77) | 68 (58 – 77) | 66 (56 – 75) |
| Sex (% female) | 44.30 | 43.82 | 46.44 |
| **Smoking status (%)** |  |  |  |
| Never | 47.75 | 47.96 | 46.84 |
| Ex-smoker | 33.46 | 33.26 | 34.36 |
| Current smoker | 18.78 | 18.78 | 18.80 |
| **IMD (%)** |  |  |  |
| 1 (Least deprived) | 16.97 | 17.28 | 15.58 |
| 2 | 18.88 | 19.17 | 17.58 |
| 3 | 19.40 | 19.54 | 18.77 |
| 4 | 21.77 | 21.61 | 22.50 |
| 5 (Most deprived) | 22.98 | 22.41 | 22.56 |
| **Fibrotic conditions** |  |  |  |
| >=1 fibrotic condition, %, (95% CI) | 59.31  (59.17 – 59.45) | 57.12  (56.97 – 57.27) | 69.69  (69.39 – 69.99) |
| FMM, %, (95% CI) | 33.06  (32.93 – 33.19) | 30.57  (30.43 – 30.71) | 44.57  (44.25 – 44.89) |

Table 10| Baseline characteristics of those with type 2 diabetes, the exposure was prescription of metformin in the year prior to the index date. IMD: Index of multiple deprivation, FMM: fibrotic multimorbidity (two or more fibrotic conditions).

|  | **Whole cohort** | **Not prescribed metformin** | **Prescribed metformin** |
| --- | --- | --- | --- |
|  |  |  |  |
| Size of cohort (%) | 504,066 | 346,322 (68.61) | 158,460 (31.39) |
| Median age (IQR) | 68 (58 – 77) | 68 (58 – 77) | 66 (56 – 74) |
| Sex (% female) | 44.30 | 45.07 | 42.61 |
| **Smoking status (%)** |  |  |  |
| Never | 47.75 | 47.08 | 49.23 |
| Ex-smoker | 33.46 | 34.33 | 31.58 |
| Current smoker | 18.78 | 18.60 | 19.19 |
| **IMD (%)** |  |  |  |
| 1 (Least deprived) | 16.97 | 18.04 | 14.63 |
| 2 | 18.88 | 19.62 | 17.27 |
| 3 | 19.40 | 19.78 | 18.57 |
| 4 | 21.77 | 20.75 | 23.99 |
| 5 (Most deprived) | 22.98 | 21.81 | 25.54 |
| **Fibrotic conditions** |  |  |  |
| >=1 fibrotic condition, %, (95% CI) | 59.31  (59.17 – 59.45) | 60.67  (60.51 – 60.83) | 56.35  (56.11 – 56.59) |
| FMM, %, (95% CI) | 33.06  (32.93 – 33.19) | 34.56  (34.40 – 34.72) | 29.78  (29.55 – 30.01) |

## **Type 1 diabetes prevalence and odds ratios**

Table 11| Prevalence of fibrotic conditions per exposure group based upon evidence of microvascular complications.

|  | **Whole cohort %, (95% CI)** |  | **No evidence of microvascular complications %, (95% CI)** |  | **Microvascular complications %, (95% CI)** |
| --- | --- | --- | --- | --- | --- |
|  |  |  |  |  |  |
| **Atherosclerosis** | 3.08  (2.74 – 3.43) |  | 2.72  (2.28 – 3.15) |  | 3.54  (2.99 – 4.09) |
| **Biliary** | 2.44  (2.13 – 2.75) |  | 2.83  (2.38 – 3.27) |  | 1.96  (1.54 – 2.37) |
| **Blood vessel** | 1.47  (1.23 – 1.71) |  | 1.27  (0.97 – 1.56) |  | 1.72  (1.33 – 2.11) |
| **Cardiomyopathy** | 14.39  (13.39 – 15.09) |  | 12.13  (11.26 – 13.00) |  | 17.21  (16.08 – 18.34) |
| **Integumentary** | 5.08  (4.64 – 5.52) |  | 4.26  (3.72 – 4.80) |  | 6.10  (5.39 - 6.82) |
| **Intest/ panc** | 17.11  (16.36 – 17.86) |  | 17.10  (16.09 – 18.10) |  | 17.12  (15.99 – 18.24) |
| **Liver** | 3.98  (3.59 – 4.37) |  | 3.98  (3.46 – 4.50) |  | 3.98  (3.40 – 4.57) |
| **Reproductive** | 2.39  (1.93 – 2.85) |  | 2.56  (1.93 – 3.19) |  | 2.18  (1.50 – 2.86) |
| **Lung** | 5.00  (4.56 – 5.43) |  | 4.45  (3.90 – 5.00) |  | 5.68  (4.99 – 6.37) |
| **Skeletal** | 17.03  (16.28 – 17.78) |  | 14.98  (14.02 – 15.93) |  | 19.61  (18.42 – 20.80) |
| **Systemic** | 1.58  (1.33 – 1.83) |  | 1.13  (0.85 –1.42) |  | 2.14  (1.71 – 2.58) |
| **Urinary** | 4.09  (3.69 – 4.48) |  | 2.88  (2.44 – 3.33) |  | 5.59  (4.90 – 6.28) |
| **Valve** | 3.01  (2.67 – 3.35) |  | 2.40  (1.99 – 2.81) |  | 3.77  (3.20 – 4.34) |

Table 12 | *Crude and fully adjusted odds ratios for the development of each fibrotic condition in the three years post index date. Fully adjusted analyses included adjustment for age, sex, smoking status, deprivation, number of fibrotic conditions at baseline and hypertension severity.*

|  | **Microvascular complications** | | |
| --- | --- | --- | --- |
| **Fibrotic condition** | **Crude OR (99% CI)** | **Fully adjusted**  **OR (99% CI)** | **p-value** |
| **Atherosclerosis** | 2.11  (1.39 - 3.20) | 1.81  (1.18 – 2.77) | <0.001 |
| **Biliary** | 1.42  (0.81 - 2.50) | 1.22  (0.69 – 2.17) | 0.365 |
| **Blood vessel** | 0.78  (0.38 – 1.58) | 0.69  (0.33 – 1.41) | 0.161 |
| **Cardiomyopathy** | 1.73  (1.31 – 2.30) | 1.53  (1.15 – 2.04) | <0.001 |
| **Integumentary** | 0.98  (0.59 – 1.63) | 0.96  (0.57 – 1.61) | 0.831 |
| **Intest/ panc** | 1.26  (1.00 – 1.58) | 1.10  (0.87 – 1.40) | 0.295 |
| **Liver** | 0.97  (0.66 – 1.43) | 0.84  (0.57 – 1.26) | 0.276 |
| **Lung** | 1.47  (1.15 – 1.89) | 1.24  (0.96– 1.61) | 0.030 |
| **Reproductive** | 0.91  (0.32 – 2.57) | 0.96  (0.33 – 2.75) | 0.915 |
| **Skeletal** | 1.33  (1.06 – 1.67) | 1.18  (0.93 – 1.49) | 0.068 |
| **Systemic** | 1.27  (0.63 – 2.56) | 1.18  (0.57 – 2.46) | 0.549 |
| **Urinary** | 1.92  (1.07 – 3.45) | 1.82  (1.00 – 3.29) | 0.010 |
| **Valve** | 1.45  (1.00 – 2.10) | 1.17  (0.80 – 1.71) | 0.299 |

Table 13| Prevalence of fibrotic conditions per exposure group defined using measures of albumin to creatinine ratio (ACR) and glomerular filtration rate (eGFR). Elevated ACR and eGFR was defined as ACR ≥3 mg/mmol, eGFR <60 mL/min/1.73 m^2^ respectively. Where the prevalence was less than 1%, this was reported as such.

|  | **Whole cohort *** | **ACR norm / eGFR norm** | **ACR elevated/ eGFR norm** | **ACR elevated/ eGFR decreased** | **ACR norm / eGFR decreased** |
| --- | --- | --- | --- | --- | --- |
| **Atherosclerosis** | 3.24  (2.82 – 3.66) | 1.94  (1.51 – 2.37) | 3.23  (2.28 – 4.18) | 8.19  (6.30 – 10.08) | 5.06  (3.35 – 6.76) |
| **Biliary** | 2.50  (2.12 – 2.87) | 2.15  (1.70 – 2.60) | 2.48  (1.64 – 3.31) | 3.47  (2.21 – 4.74) | 3.48  (2.05 – 4.90) |
| **Blood vessel** | 3.28  (2.86 – 3.71) | 2.40  (1.92 – 2.88) | 3.16  (2.22 – 4.09) | 6.33  (4.65 – 8.01) | 5.21  (3.48 – 6.94) |
| **Cardiomyopathy** | 15.53  (14.66 – 6.39) | 11.14  (10.16 – 12.12) | 16.54  (14.46 – 18.45) | 28.54  (25.42 – 31.65) | 24.49  (21.14 – 27.84) |
| **Integumentary** | 10.52  (9.79 – 11.25) | 9.39  (8.49 – 10.30) | 10.74  (9.08 – 12.41) | 14.39  (11.97 – 16.82) | 12.16  (9.62 – 14.71) |
| **Intest/ panc** | 18.01  (17.09 – 8.93) | 14.60  (13.50 – 15.70) | 18.93  (16.83 – 21.04) | 26.43  (23.38 – 29.47) | 26.69  (23.35 – 30.14) |
| **Liver** | 3.92  (3.46 – 4.39) | 3.41  (2.84 – 3.97) | 4.58  (3.46 – 5.71) | 5.33  (3.78 – 6.89) | 3.95  (2.43 – 5.47) |
| **Reproductive** | 2.69  (2.10 – 3.28) | 3.52  (2.62 – 4.42) | 2.55  (1.23 – 3.87) | <1.00 | <1.00 |
| **Lung** | 5.19  (4.66 – 5.72) | 3.41  (2.84 – 3.97) | 5.26  (4.06 – 6.46) | 11.41  (9.22 – 13.61) | 8.21  (6.08- 10.36) |
| **Skeletal** | 24.10  (23.08 – 5.12) | 20.20  (18.95 – 21.45) | 23.59  (21.31 – 25.87) | 36.35  (33.03 – 39.67) | 33.97  (30.28 – 37.65) |
| **Systemic** | 1.84  (1.52 – 2.16) | 1.01  (0.70 – 1.32) | 1.50  (0.85 – 2.16) | 5.58  (4.00 – 7.17) | 3.00  (1.67 – 4.33) |
| **Urinary** | 6.00  (5.44 – 6.57) | 2.50  (2.01 – 2.99) | 5.41  (4.19 – 6.62) | 20.47  (17.69 – 23.26) | 10.74  (8.33 – 13.15) |
| **Valve** | 3.89  (3.43 – 4.36) | 2.30  (1.83 – 2.76) | 3.38  (2.41 – 4.35) | 9.43  (7.41 – 11.45) | 7.90  (5.80 – 10.00) |

*Table 14| Crude and fully adjusted odds ratios for the development of each fibrotic condition in the three years post index date. Fully adjusted analyses included adjustment for age, sex, smoking status, deprivation, number of fibrotic conditions at baseline and hypertension severity.*

|  | 1. **ACR elevated/ eGFR norm** | | | 1. **ACR elevated/ eGFR decreased** | | | 1. **ACR norm/ eGFR decreased** | | |
| --- | --- | --- | --- | --- | --- | --- | --- | --- | --- |
| **Fibrotic condition** | **Crude OR (99% CI)** | **Fully adjusted**  **OR (99% CI)** | **p-value** | **Crude OR**  **(99% CI)** | **Fully adjusted**  **OR (99% CI)** | **p-value** | **Crude OR (99% CI)** | **Fully adjusted**  **OR (99% CI)** | **p-value** |
|  |  |  |  |  |  |  |  |  |  |
| **Atherosclerosis** | 2.43  (1.35 - 4.39) | 1.75  (0.95 - 3.21) | 0.018 | 4.34  (2.40 - 7.85) | 1.85  (0.96 - 3.54) | 0.015 | 2.08  (0.94 - 4.62) | 1.08  (0.47 - 2.52) | 0.810 |
| **Biliary** | 1.06  (0.43 - 2.63) | 0.72  (0.29 - 1.84) | 0.372 | 2.26  (0.98 - 2.63) | 1.01  (0.40 - 2.54) | 0.974 | 0.82  (0.21 - 3.23) | 0.39  (0.09 - 1.65) | 0.094 |
| **Blood vessel** | 0.72  (0.22 - 2.32) | 0.59  (0.18 - 1.94) | 0.256 | 1.20  (0.37 - 3.90) | 0.54  (0.16 - 1.90) | 0.211 | 1.52  (0.47 - 4.94) | 0.63  (0.18 - 2.21) | 0.338 |
| **Cardiomyopathy** | 1.68  (1.15 - 2.46) | 1.29  (0.87 - 1.91) | 0.095 | 2.33  (1.51 - 3.60) | 1.21  (0.74 - 1.97) | 0.310 | 2.08  (1.29 - 3.38) | 1.31  (0.78 - 2.23) | 0.182 |
| **Integumentary** | 0.92  (0.39 - 2.17) | 0.78  (0.32 - 1.89) | 0.475 | 2.02  (0.92 - 4.44) | 1.19  (0.48 - 2.95) | 0.623 | 2.17  (0.94 - 4.99) | 1.58  (0.62 - 4.07) | 0.208 |
| **Intest/ panc** | 1.49  (1.08 - 2.04) | 1.21  (0.87 - 1.68) | 0.141 | 2.45  (1.73 - 3.45) | 1.18  (0.80 - 1.68) | 0.278 | 1.67  (1.09 - 2.55) | 0.90  (0.57 - 1.43) | 0.575 |
| **Liver** | 1.75  (1.04 - 2.93) | 1.25  (0.73 - 2.14) | 0.280 | 1.15  (0.56 - 2.37) | 0.67  (0.31 - 1.46) | 0.189 | 1.26  (0.59 - 2.71) | 0.90  (0.40 – 2.04) | 0.747 |
| **Lung** | 2.44  (1.70 - 3.52) | 1.95  (1.33 - 2.85) | <0.001 | 5.84  (4.10 - 8.32) | 2.31  (1.56 - 3.42) | <0.001 | 2.80  (1.78 - 4.39) | 1.09  (0.67 - 1.79) | 0.645 |
| **Reproductive** | 1.32  (0.28 - 6.22) | 1.23  (0.25 - 6.08) | 0.743 | 1.62  (0.29 - 9.06) | 1.19  (0.17 - 8.15) | 0.814 | 0.69  (0.05 - 10.41) | 0.65  (0.04 - 11.35) | 0.743 |
| **Skeletal** | 1.42  (1.03 - 1.96) | 1.13  (0.81 - 1.58) | 0.338 | 2.19  (1.53 - 3.15) | 0.99  (0.66 - 1.48) | 0.934 | 2.47  (1.70 - 3.59) | 1.20  (0.80 - 1.82) | 0.250 |
| **Systemic** | 2.40  (0.88 - 6.54) | 1.66  (0.58 - 4.70) | 0.212 | 4.18  (1.53 - 11.39) | 2.24  (0.73 - 6.90) | 0.064 | 3.00  (0.92 - 9.80) | 2.27  (0.61 - 8.50) | 0.212 |
| **Urinary** | 3.52  (1.60 - 7.78) | 3.55  (1.57 - 7.99) | <0.001 | 4.79  (2.01 - 11.45) | 4.40  (1.67 - 11.61) | <0.001 | 2.40  (0.77 - 7.47) | 2.41  (0.71 - 8.14) | 0.063 |
| **Valve** | 2.11  (1.25 - 3.56) | 1.53  (0.89 - 2.63) | 0.044 | 4.56  (2.76 - 7.53) | 1.46  (0.84 - 2.54) | 0.078 | 2.45  (1.28 - 4.70) | 0.85  (0.43 - 1.71) | 0.555 |

Table 15 | Prevalence of fibrotic conditions per exposure group defined using multiple measures of albumin to creatinine ratio (ACR) and glomerular filtration rate (eGFR). Elevated ACR and eGFR was defined as ACR ≥3 mg/mmol, eGFR <60 mL/min/1.73 m^2^ respectively.

|  | **Whole cohort *** | **ACR norm / eGFR norm** | **Persistent ACR elevated/ eGFR norm** | **Persistent ACR elevated/ eGFR decreased** | **ACR norm / Persistent eGFR decreased** |
| --- | --- | --- | --- | --- | --- |
| **Atherosclerosis** | 3.47  (2.98 – 3.96) | 1.94  (1.45 – 2.42) | 3.60  (2.38 – 4.82) | 8.46  (6.41 – 10.51) | 5.14  (3.43 – 6.85) |
| **Biliary** | 2.42  (2.00 – 2.83) | 2.13  (1.62 – 2.64) | 2.25  (1.27 – 3.22) | 2.82  (1.60 – 4.04) | 3.58  (2.14 – 5.02) |
| **Blood vessel** | 1.33  (1.02 – 1.64) | <1.00 | 1.35  (0.59 – 2.10) | 3.39  (2.05 – 4.72) | 1.40  (0.49 – 2.31) |
| **Cardiomyopathy** | 16.28  (15.29 – 17.27) | 11.46  (10.34 – 12.58) | 15.86  (13.56 – 18.26) | 29.90  (26.53 – 33.27) | 25.08  (21.72 – 28.43) |
| **Integumentary** | 5.40  (4.79 – 6.00) | 4.20  (3.49 – 4.90) | 5.06  (3.62 – 6.50) | 9.45  (7.30 – 11.60) | 7.17  (5.17 – 9.16) |
| **Intest/ panc** | 17.67  (16.64 – 18.69) | 13.82  (12.60 – 15.03) | 17.66  (15.15 – 20.17) | 27.93  (24.62 – 31.23) | 24.92  (21.58 – 28.27) |
| **Liver** | 3.52  (3.03 – 4.02) | 3.00  (2.40 – 3.60) | 4.72  (3.33 – 6.12) | 4.94  (3.34 – 6.53) | 2.80  (1.53 – 4.08) |
| **Reproductive** | 1.84  (1.28 – 2.40) | 2.84  (1.91 – 3.77) | <1.00 | <1.00 | <1.00 |
| **Lung** | 5.06  (4.47 – 5.65) | 2.94  (2.34 – 3.53) | 5.06  (3.62 – 6.50) | 11.71  (9.34 – 14.07) | 7.94  (5.85 – 10.04) |
| **Skeletal** | 18.45  (17.41 – 19.49) | 13.20  (2.34 – 3.53) | 18.11  (15.58 – 20.64) | 32.86  (29.41 – 36.32) | 28.35  (24.86 – 31.84) |
| **Systemic** | 1.72  (1.37 – 2.07) | <1.00 | 1.24  (0.51 – 1.96) | 5.36  (3.70 – 7.02) | 3.12  (1.77 – 4.46) |
| **Urinary** | 6.31  (5.66 – 6.97) | 2.52  (1.97 – 3.07) | 5.51  (4.01 – 7.01) | 18.62  (15.75 – 21.48) | 12.15  (9.62 – 14.68) |
| **Valve** | 3.65  (3.15 – 4.16) | 1.97  (1.48 – 2.46) | 2.59  (1.54 – 3.63) | 9.03  (6.92 – 11.14) | 7.32  (5.31 – 9.34) |

Table 16| *Crude and fully adjusted odds ratios for the development of each fibrotic condition in the three years post index date. Fully adjusted analyses included adjustment for age, sex, smoking status, deprivation, number of fibrotic conditions at baseline and hypertension severity.*

|  | 1. **Persistent ACR elevated/ eGFR norm** | | | 1. **Persistent ACR elevated/ Persistent eGFR decreased** | | | 1. **ACR norm / Persistent eGFR decreased** | | |
| --- | --- | --- | --- | --- | --- | --- | --- | --- | --- |
| **Fibrotic condition** | **Crude OR (99% CI)** | **Fully adjusted**  **OR (99% CI)** | **p-value** | **Crude OR**  **(99% CI)** | **Fully adjusted**  **OR (99% CI)** | **p-value** | **Crude OR (99% CI)** | **Fully adjusted**  **OR (99% CI)** | **p-value** |
|  |  |  |  |  |  |  |  |  |  |
| **Atherosclerosis** | 1.89  (0.92 - 3.87) | 1.33  (0.63 - 2.78) | 0.323 | 3.69  (1.94 - 7.04) | 1.65  (0.81 - 3.38) | 0.072 | 1.86  (0.82 - 4.19) | 1.00  (0.42 - 2.41) | 0.992 |
| **Biliary** | 0.95  (0.29 - 3.13) | 0.67  (0.20 - 2.64) | 0.394 | 2.01  (0.75 - 5.41) | 0.89  (0.30 - 2.64) | 0.778 | 1.34  (0.41 - 4.41) | 0.70  (0.19 - 2.52) | 0.473 |
| **Blood vessel** | 0.92  (0.25 - 3.38) | 0.77  (0.21 - 2.86) | 0.602 | 0.94  (0.23 - 3.91) | 0.47  (0.10 - 2.13) | 0.199 | 2.32  (0.81 - 6.61) | 1.15  (0.36 - 3.68) | 0.758 |
| **Cardiomyopathy** | 1.77  (1.15 - 2.72) | 1.34  (0.86 - 2.09) | 0.091 | 2.07  (1.29 - 3.33) | 1.07  (0.62 - 1.82) | 0.760 | 1.87  (1.14 - 3.08) | 1.19  (0.69 - 2.06) | 0.419 |
| **Integumentary** | 1.02  (0.38 - 2.72) | 0.92  (0.33 - 2.55) | 0.831 | 1.80  (0.75 - 4.36) | 1.08  (0.39 - 2.98) | 0.844 | 2.11  (0.89 - 4.99) | 1.49  (0.39 - 3.99) | 0.293 |
| **Intest/ panc** | 1.18  (0.81 - 1.72) | 0.90  (0.61 - 1.34) | 0.512 | 1.73  (1.18 - 2.55) | 0.80  (0.52 - 1.25) | 0.199 | 1.36  (0.88 - 2.08) | 0.67  (0.41 - 1.07) | 0.027 |
| **Liver** | 1.86  (1.05 - 3.33) | 1.39  (0.76 - 2.53) | 0.160 | 1.19  (0.57 - 2.48) | 0.80  (0.36 - 1.76) | 0.464 | 0.72  (0.28 - 1.82) | 0.53  (0.20 - 2.53) | 0.093 |
| **Lung** | 2.60  (1.73 - 3.92) | 2.07  (1.35 - 3.17) | <0.001 | 5.43  (1.73 - 3.92) | 2.18  (1.43 - 3.17) | <0.001 | 2.98  (1.92 - 4.63) | 1.19  (0.73 - 1.94) | 0.352 |
| **Reproductive** | 0.49  (0.03 - 7.74) | 0.46  (0.03 - 7.59) | 0.478 | 2.48  (0.49 - 12.48) | 2.25  (0.35 - 14.55) | 0.263 | 0.68  (0.04 - 10.69) | 0.68  (0.04 - 12.88) | 0.737 |
| **Skeletal** | 1.51  (1.04 - 2.19) | 1.18  (0.80 - 1.74) | 0.266 | 2.14  (1.44 - 3.18) | 1.00  (0.65 - 1.56) | 0.985 | 2.68  (1.84 - 3.91) | 1.33  (0.87 - 2.03) | 0.083 |
| **Systemic** | 2.17  (0.68 - 6.92) | 1.63  (0.49 - 5.46) | 0.299 | 2.84  (0.89 - 9.09) | 1.66  (0.45 - 6.06) | 0.313 | 2.29  (0.64 - 8.22) | 1.82  (0.43 - 7.67) | 0.283 |
| **Urinary** | 2.39  (0.98 - 5.84) | 2.37  (0.94 - 5.95) | 0.016 | 3.49  (1.42 - 8.55) | 2.88  (1.04 - 7.95) | 0.007 | 1.53  (0.46 - 5.06) | 1.34  (0.37 - 5.95) | 0.559 |
| **Valve** | 2.42  (1.34 - 4.35) | 1.73  (0.94 - 3.17) | 0.020 | 4.36  (2.51 - 7.55) | 1.52  (0.83 - 2.80) | 0.075 | 3.12  (1.69 - 5.78) | 1.20  (0.62 - 2.35) | 0.476 |

## **Type 1 diabetes sensitivity analyses**

| **Microvascular complications** | | |
| --- | --- | --- |
| **Fibrotic condition** | **Fully adjusted**  **OR (99% CI)** | **Fully adjusted**  **OR + CCI (99% CI)** |
| **Atherosclerosis** | 1.81  (1.18 – 2.77) | 1.81  (1.18 – 2.77) |
| **Biliary** | 1.22  (0.69 – 2.17) | 1.21  (0.59 – 1.90) |
| **Blood vessel** | 0.69  (0.33 – 1.41) | 0.70  (0.34 – 1.44) |
| **Cardiomyopathy** | 1.53  (1.15 – 2.04) | 1.54  (1.16 – 1.04) |
| **Integumentary** | 0.96  (0.57 – 1.61) | 0.96  (0.57 – 1.62) |
| **Intest/ panc** | 1.10  (0.87 – 1.40) | 1.09  (0.86 – 1.71) |
| **Liver** | 0.84  (0.57 – 1.26) | 0.84  (0.56 – 1.25) |
| **Lung** | 1.24  (0.96– 1.61) | 1.23  (0.95 – 1.60) |
| **Reproductive** | 0.96  (0.33 – 2.75) | 0.93  (0.32 – 2.68) |
| **Skeletal** | 1.18  (0.93 – 1.49) | 1.16  (0.92 – 1.47) |
| **Systemic** | 1.18  (0.57 – 2.46) | 1.15  (0.55 – 2.41) |
| **Urinary** | 1.82  (1.00 – 3.29) | 1.79  (0.98 – 3.24) |
| **Valve** | 1.17  (0.80 – 1.71) | 1.17  (0.80 – 1.72) |

Table 17| Fully adjusted odds ratios for the development of each fibrotic condition in the three years post index date. Fully adjusted analyses included adjustment for age, sex, smoking status, deprivation, number of fibrotic conditions at baseline and hypertension severity. This sensitivity analysis included Charlson comorbidity index (CCI) as a covariate, where findings differ these are made apparent with red text.

|  | **ACR elevated/ eGFR norm** | |  | **ACR elevated/ eGFR decreased** | |  | **ACR norm / eGFR decreased** | |
| --- | --- | --- | --- | --- | --- | --- | --- | --- |
| **Fibrotic condition** | **Fully adjusted**  **OR (99% CI)** | **Fully adjusted**  **OR + CCI (99% CI)** |  | **Fully adjusted**  **OR (99% CI)** | **Fully adjusted**  **OR + CCI (99% CI)** |  | **Fully adjusted**  **OR (99% CI)** | **Fully adjusted**  **OR + CCI (99% CI)** |
|  |  |  |  |  |  |  |  |  |
| **Atherosclerosis** | 1.75  (0.95 - 3.21) | 1.80  (0.94 – 3.46) |  | 1.85  (0.96 - 3.54) | 2.11  (1.07 – 4.16) |  | 1.08  (0.47 - 2.52) | 1.30  (0.55 – 3.10) |
| **Biliary** | 0.72  (0.29 - 1.84) | 0.82  (0.32 – 2.12) |  | 1.01  (0.40 - 2.54) | 0.96  (0.36 – 2.59) |  | 0.39  (0.09 - 1.65) | 0.49  (0.12 – 2.11) |
| **Blood vessel** | 0.59  (0.18 - 1.94) | 0.61  (0.18 – 2.07) |  | 0.54  (0.16 - 1.90) | 0.59  (0.16 – 2.10) |  | 0.63  (0.18 - 2.21) | 0.68  (0.19 – 2.46) |
| **Cardiomyopathy** | 1.29  (0.87 - 1.91) | 1.28  (0.83 – 1.96) |  | 1.21  (0.74 - 1.97) | 1.24  (0.75 – 2.07) |  | 1.31  (0.78 - 2.23) | 1.36  (0.77 – 2.39) |
| **Integumentary** | 0.78  (0.32 - 1.89) | 0.91  (0.37 – 2.28) |  | 1.19  (0.48 - 2.95) | 1.23  (0.46 – 3.28) |  | 1.58  (0.62 - 4.07) | 1.62  (0.60 – 4.40) |
| **Intest/ panc** | 1.21  (0.87 - 1.68) | 1.15  (0.80 – 1.65) |  | 1.18  (0.80 - 1.68) | 1.11  (0.73 – 1.69) |  | 0.90  (0.57 - 1.43) | 0.97  (0.60 – 1.59) |
| **Liver** | 1.25  (0.73 - 2.14) | 1.26  (0.72 – 2.21) |  | 0.67  (0.31 - 1.46) | 0.64  (0.29 – 1.43) |  | 0.90  (0.40 - 2.05) | 0.89  (0.38 – 2.08) |
| **Lung** | 1.95  (1.33 - 2.85) | 2.03  (1.36 – 3.01) |  | 2.31  (1.56 - 3.42) | 2.18  (1.45 – 3.28) |  | 1.09  (0.67 - 1.79) | 2.18  (1.45 – 3.28) |
| **Reproductive** | 1.23  (0.25 - 6.08) | 1.26  (0.24 – 6.58) |  | 1.19  (0.17 - 8.15) | 1.32  (0.20 – 8.78) |  | 0.65  (0.04 - 11.35) | 0.75  (0.04 – 13.28) |
| **Skeletal** | 1.13  (0.81 - 1.58) | 1.09  (0.76 – 1.56) |  | 0.99  (0.66 - 1.48) | 0.90  (0.59 – 1.39) |  | 1.20  (0.80 - 1.82) | 1.04  (0.66 – 1.64) |
| **Systemic** | 1.66  (0.58 - 4.70) | 1.91  (0.63 – 5.81) |  | 2.24  (0.73 - 6.90) | 2.33  (0.67 – 8.07) |  | 2.27  (0.61 - 8.50) | 2.82  (0.70 – 11.32) |
| **Urinary** | 3.55  (1.57 - 7.99) | 3.88  (1.58 – 9.50) |  | 4.40  (1.67 - 11.61) | 4.43  (1.55 – 12.68) |  | 2.41  (0.71 - 8.14) | 2.91  (0.82 – 10.36) |
| **Valve** | 1.53  (0.89 - 2.63) | 1.44  (0.80 – 2.58) |  | 1.46  (0.84 - 2.54) | 1.40  (0.77 – 2.52) |  | 0.85  (0.43 - 1.71) | 0.79  (0.38 – 1.67) |

Table 18| *Fully adjusted odds ratios for the development of each fibrotic condition in the three years post index date. Fully adjusted analyses included adjustment for age, sex, smoking status, deprivation, number of fibrotic conditions at baseline and hypertension severity. This sensitivity analysis included Charlson comorbidity index (CCI) as a covariate, where findings differ these are made apparent with red text.*

Table 19 | Fully adjusted odds ratios for the development of each fibrotic condition in the three years post index date. Fully adjusted analyses included adjustment for age, sex, smoking status, deprivation, number of fibrotic conditions at baseline and hypertension severity. This sensitivity analysis included Charlson comorbidity index (CCI) as a covariate, where findings differ these are made apparent with red text.

|  | **Persistent ACR elevated/ eGFR norm** | |  | **Persistent ACR elevated/ Persistent eGFR decreased** | |  | **ACR norm / Persistent eGFR decreased** | |
| --- | --- | --- | --- | --- | --- | --- | --- | --- |
| **Fibrotic condition** | **Fully adjusted**  **OR (99% CI)** | **Fully adjusted**  **OR + CCI (99% CI)** |  | **Fully adjusted**  **OR (99% CI)** | **Fully adjusted**  **OR + CCI (99% CI)** |  | **Fully adjusted**  **OR (99% CI)** | **Fully adjusted**  **OR + CCI (99% CI)** |
|  |  |  |  |  |  |  |  |  |
| **Atherosclerosis** | 1.33  (0.63 - 2.78) | 1.25  (0.57 – 2.71) |  | 1.65  (0.81 - 3.38) | 1.61  (0.76 – 3.41) |  | 1.00  (0.42 - 2.41) | 1.07  (0.44 – 2.62) |
| **Biliary** | 0.67  (0.20 - 2.64) | 0.68  (0.18 – 2.56) |  | 0.89  (0.30 - 2.64) | 0.96  (0.30 – 3.10) |  | 0.70  (0.19 - 2.52) | 0.92  (0.24 – 3.47) |
| **Blood vessel** | 0.77  (0.21 - 2.86) | 0.79  (0.20 – 3.14) |  | 0.47  (0.10 - 2.13) | 0.51  (0.11 – 2.38) |  | 1.15  (0.36 - 3.68) | 1.25  (0.37 – 4.18) |
| **Cardiomyopathy** | 1.34  (0.86 - 2.09) | 1.47  (0.91 – 2.38) |  | 1.07  (0.62 - 1.82) | 1.11  (0.63 – 1.95) |  | 1.19  (0.69 - 2.06) | 1.27  (0.71 – 2.28) |
| **Integumentary** | 0.92  (0.33 - 2.55) | 1.08  (0.37 – 3.10) |  | 1.08  (0.39 - 2.98) | 1.04  (0.35 – 3.12) |  | 1.49  (0.39 - 3.99) | 1.50  (0.53 – 4.23) |
| **Intest/ panc** | 0.90  (0.61 - 1.34) | 0.85  (0.55 – 1.31) |  | 0.80  (0.52 - 1.25) | 0.77  (0.48 – 1.22) |  | 0.67  (0.41 - 1.07) | 0.65  (0.39 – 1.09) |
| **Liver** | 1.39  (0.76 - 2.53) | 1.31  (0.69 – 2.46) |  | 0.80  (0.36 - 1.76) | 0.70  (0.69 – 2.46) |  | 0.53  (0.20 - 2.53) | 0.55  (0.20 – 1.48) |
| **Lung** | 2.07  (1.35 - 3.17) | 2.32  (1.48 – 3.61 |  | 2.18  (1.43 - 3.17) | 2.21  (1.42 – 3.44) |  | 1.19  (0.73 - 1.94) | 1.25  (0.76 – 2.08) |
| **Reproductive** | 0.46  (0.03 - 7.59) | 0.46  (0.03 – 7.65) |  | 2.25  (0.35 - 14.55) | 2.26  (0.37 – 13.86) |  | 0.68  (0.04 - 12.88) | 0.79  (0.04 – 14.68) |
| **Skeletal** | 1.18  (0.80 - 1.74) | 1.17  (0.77 – 1.79) |  | 1.00  (0.65 - 1.56) | 0.94  (0.58 – 1.51) |  | 1.33  (0.87 - 2.03) | 1.31  (0.83 – 2.07) |
| **Systemic** | 1.63  (0.49 - 5.46) | 1.70  (0.48 – 6.07) |  | 1.66  (0.45 - 6.06) | 1.29  (0.30 – 5.58) |  | 1.82  (0.43 - 7.67) | 2.27  (0.51 – 10.17) |
| **Urinary** | 2.37  (0.94 - 5.95) | 2.15  (0.80 – 5.80) |  | 2.88  (1.04 - 7.95) | 2.84  (0.98 – 8.25) |  | 1.34  (0.37 - 5.95) | 1.17  (0.29 – 4.75) |
| **Valve** | 1.73  (0.94 - 3.17) | 1.88  (0.98 – 3.58) |  | 1.52  (0.83 - 2.80) | 1.38  (0.71 – 2.68) |  | 1.20  (0.62 - 2.35) | 1.22  (0.60 – 2.48) |

Table 20 | Fully adjusted odds ratios for the development of each fibrotic condition in the three years post index date. Fully adjusted analyses included adjustment for age, sex, smoking status, deprivation, number of fibrotic conditions at baseline and hypertension severity. This sensitivity analysis included oral corticosteroids (OCS) as a covariate, where findings differ these are made apparent with red text.

| **Microvascular complications** | | |
| --- | --- | --- |
| **Fibrotic condition** | **Fully adjusted**  **OR (99% CI)** | **Fully adjusted**  **OR + OCS (99% CI)** |
|  |  |  |
| **Atherosclerosis** | 1.81  (1.18 – 2.77) | 1.81  (1.18 – 2.77) |
| **Biliary** | 1.22  (0.69 – 2.17) | 1.21  (0.68 – 2.15) |
| **Blood vessel** | 0.69  (0.33 – 1.41) | 0.69  (0.33 – 1.42) |
| **Cardiomyopathy** | 1.53  (1.15 – 2.04) | 1.54  (1.15 – 2.05) |
| **Integumentary** | 0.96  (0.57 – 1.61) | 0.96  (0.57 – 1.61) |
| **Intest/ panc** | 1.10  (0.87 – 1.40) | 1.10  (0.87 – 1.40) |
| **Liver** | 0.84  (0.57 – 1.26) | 0.85  (0.57 – 1.26) |
| **Lung** | 1.24  (0.96– 1.61) | 1.25  (0.97 – 1.62) |
| **Reproductive** | 0.96  (0.33 – 2.75) | 0.95  (0.33– 2.74) |
| **Skeletal** | 1.18  (0.93 – 1.49) | 1.19  (0.94 – 1.50) |
| **Systemic** | 1.18  (0.57 – 2.46) | 1.18  (0.57 – 2.44) |
| **Urinary** | 1.82  (1.00 – 3.29) | 1.81  (1.00 – 3.28) |
| **Valve** | 1.17  (0.80 – 1.71) | 1.17  (0.79 – 1.71) |

Table 21 | Fully adjusted odds ratios for the development of each fibrotic condition in the three years post index date. Fully adjusted analyses included adjustment for age, sex, smoking status, deprivation, number of fibrotic conditions at baseline and hypertension severity. This sensitivity analysis included oral corticosteroids (OCS) as a covariate, where findings differ these are made apparent with red text.

|  | **ACR elevated/ eGFR norm** | |  | **ACR elevated/ eGFR decreased** | |  | **ACR norm / eGFR decreased** | |
| --- | --- | --- | --- | --- | --- | --- | --- | --- |
| **Fibrotic condition** | **Fully adjusted**  **OR (99% CI)** | **Fully adjusted**  **OR + OCS (99% CI)** |  | **Fully adjusted**  **OR (99% CI)** | **Fully adjusted**  **OR + OCS (99% CI)** |  | **Fully adjusted**  **OR (99% CI)** | **Fully adjusted**  **OR + OCS (99% CI)** |
|  |  |  |  |  |  |  |  |  |
| **Atherosclerosis** | 1.75  (0.95 - 3.21) | 1.75  (0.95 – 3.21) |  | 1.85  (0.96 - 3.54) | 1.85  (0.96 - 3.54) |  | 1.08  (0.47 - 2.52) | 1.08  (0.47 - 2.52) |
| **Biliary** | 0.72  (0.29 - 1.84) | 0.72  (0.28 - 1.83) |  | 1.01  (0.40 - 2.54) | 1.00  (0.40 - 2.52) |  | 0.39  (0.09 - 1.65) | 0.39  (0.09 - 1.63) |
| **Blood vessel** | 0.59  (0.18 - 1.94) | 0.59  (0.18 - 1.94) |  | 0.54  (0.16 - 1.90) | 0.55  (0.16 - 1.91) |  | 0.63  (0.18 - 2.21) | 0.63  (0.18 - 2.21) |
| **Cardiomyopathy** | 1.29  (0.87 - 1.91) | 1.29  (0.87 - 1.91) |  | 1.21  (0.74 - 1.97) | 1.21  (0.74 - 1.97) |  | 1.31  (0.78 - 2.23) | 1.31  (0.78 - 2.23) |
| **Integumentary** | 0.78  (0.32 - 1.89) | 0.78  (0.32 - 1.89) |  | 1.19  (0.48 - 2.95) | 1.20  (0.48 - 2.97) |  | 1.58  (0.62 - 4.07) | 1.59  (0.62 - 4.07) |
| **Intest/ panc** | 1.21  (0.87 - 1.68) | 1.21  (0.87 - 1.69) |  | 1.18  (0.80 - 1.68) | 1.18  (0.80 - 1.75) |  | 0.90  (0.57 - 1.43) | 0.90  (0.57 - 1.43) |
| **Liver** | 1.25  (0.73 - 2.14) | 1.25  (0.73 - 2.14) |  | 0.67  (0.31 - 1.46) | 0.67  (0.31 - 1.45) |  | 0.90  (0.40 – 2.04) | 0.90  (0.40 – 2.04) |
| **Lung** | 1.95  (1.33 - 2.85) | 1.95  (1.33 - 2.85) |  | 2.31  (1.56 - 3.42) | 2.32  (1.57 - 3.44) |  | 1.09  (0.67 - 1.79) | 1.09  (0.67 - 1.79) |
| **Reproductive** | 1.23  (0.25 - 6.08) | 1.23  (0.25 - 6.09) |  | 1.19  (0.17 - 8.15) | 1.19  (0.17 - 8.15) |  | 0.65  (0.04 - 11.35) | 0.65  (0.04 - 11.35) |
| **Skeletal** | 1.13  (0.81 - 1.58) | 1.13  (0.81 - 1.58) |  | 0.99  (0.66 - 1.48) | 0.98  (0.66 - 1.47) |  | 1.20  (0.80 - 1.82) | 1.20  (0.80 - 1.82) |
| **Systemic** | 1.66  (0.58 - 4.70) | 1.65  (0.58 - 4.68) |  | 2.24  (0.73 - 6.90) | 2.27  (0.73 – 7.05) |  | 2.27  (0.61 - 8.50) | 2.29  (0.61 - 8.61) |
| **Urinary** | 3.55  (1.57 - 7.99) | 3.55  (1.57 - 7.99) |  | 4.40  (1.67 - 11.61) | 4.39  (1.66 - 11.59) |  | 2.41  (0.71 - 8.14) | 2.41  (0.71 - 8.14) |
| **Valve** | 1.53  (0.89 - 2.63) | 1.53  (0.89 - 2.63) |  | 1.46  (0.84 - 2.54) | 1.46  (0.84 - 2.54) |  | 0.85  (0.43 - 1.71) | 0.85  (0.43 - 1.71) |

Table 22 | Fully adjusted odds ratios for the development of each fibrotic condition in the three years post index date. Fully adjusted analyses included adjustment for age, sex, smoking status, deprivation, number of fibrotic conditions at baseline and hypertension severity. This sensitivity analysis included oral corticosteroids (OCS) as a covariate, where findings differ these are made apparent with red text.

|  | **Persistent ACR elevated/ eGFR norm** | |  | **Persistent ACR elevated/ Persistent eGFR decreased** | |  | **ACR norm / Persistent eGFR decreased** | |
| --- | --- | --- | --- | --- | --- | --- | --- | --- |
| **Fibrotic condition** | **Fully adjusted**  **OR (99% CI)** | **Fully adjusted**  **OR + OCS (99% CI)** |  | **Fully adjusted**  **OR (99% CI)** | **Fully adjusted**  **OR + OCS (99% CI)** |  | **Fully adjusted**  **OR (99% CI)** | **Fully adjusted**  **OR + OCS (99% CI)** |
|  |  |  |  |  |  |  |  |  |
| **Atherosclerosis** | 1.33  (0.63 - 2.78) | 1.33  (0.63 - 2.78) |  | 1.65  (0.81 - 3.38) | 1.65  (0.81 - 3.38) |  | 1.00  (0.42 - 2.41) | 1.00  (0.42 - 2.41) |
| **Biliary** | 0.67  (0.20 - 2.64) | 0.67  (0.20 - 2.26) |  | 0.89  (0.30 - 2.64) | 0.88  (0.30 - 2.63) |  | 0.70  (0.19 - 2.52) | 0.70  (0.19 - 2.53) |
| **Blood vessel** | 0.77  (0.21 - 2.86) | 0.76  (0.20 - 2.84) |  | 0.47  (0.10 - 2.13) | 0.47  (0.10 - 2.14) |  | 1.15  (0.36 - 3.68) | 1.15  (0.36 - 3.68) |
| **Cardiomyopathy** | 1.34  (0.86 - 2.09) | 1.34  (0.86 - 2.09) |  | 1.07  (0.62 - 1.82) | 1.07  (0.62 - 1.82) |  | 1.19  (0.69 - 2.06) | 1.19  (0.68 - 2.06) |
| **Integumentary** | 0.92  (0.33 - 2.55) | 0.93  (0.34 - 2.99) |  | 1.08  (0.39 - 2.98) | 1.08  (0.39 - 2.99) |  | 1.49  (0.39 - 3.99) | 1.50  (0.56 – 4.02) |
| **Intest/ panc** | 0.90  (0.61 - 1.34) | 0.91  (0.61 - 1.35) |  | 0.80  (0.52 - 1.25) | 0.80  (0.52 - 1.24) |  | 0.67  (0.41 - 1.07) | 0.67  (0.42 - 1.07) |
| **Liver** | 1.39  (0.76 - 2.53) | 1.36  (0.75 - 2.49) |  | 0.80  (0.36 - 1.76) | 0.79  (0.36 - 1.75) |  | 0.53  (0.20 - 2.53) | 0.52  (0.19 – 1.39) |
| **Lung** | 2.07  (1.35 - 3.17) | 2.05  (1.34 - 3.14) |  | 2.18  (1.43 - 3.17) | 2.19  (1.44 – 3.35) |  | 1.19  (0.73 - 1.94) | 1.20  (0.73 - 1.95) |
| **Reproductive** | 0.46  (0.03 - 7.59) | 0.45  (0.03 - 7.42) |  | 2.25  (0.35 - 14.55) | 2.21  (0.34 - 14.47) |  | 0.68  (0.04 - 12.88) | 0.75  (0.04 – 13.81) |
| **Skeletal** | 1.18  (0.80 - 1.74) | 1.18  (0.80 - 1.75) |  | 1.00  (0.65 - 1.56) | 1.00  (0.65 - 1.56) |  | 1.33  (0.87 - 2.03) | 1.33  (0.87 - 2.03) |
| **Systemic** | 1.63  (0.49 - 5.46) | 1.72  (0.51 - 5.79) |  | 1.66  (0.45 - 6.06) | 1.66  (0.45 - 6.10) |  | 1.82  (0.43 - 7.67) | 1.89  (0.45 - 7.99) |
| **Urinary** | 2.37  (0.94 - 5.95) | 2.37  (0.94 - 5.96) |  | 2.88  (1.04 - 7.95) | 2.88  (1.04 - 7.94) |  | 1.34  (0.37 - 5.95) | 1.34  (0.37 – 4.84) |
| **Valve** | 1.73  (0.94 - 3.17) | 1.73  (0.94 - 3.17) |  | 1.52  (0.83 - 2.80) | 1.52  (0.83 - 2.80) |  | 1.20  (0.62 - 2.35) | 1.20  (0.62 - 2.35) |

| **Fibrotic condition** | **Fully adjusted**  **OR (99% CI)** | **Fully adjusted**  **OR + diabetes duration (99% CI)** |
| --- | --- | --- |
|  |  |  |
| **Atherosclerosis** | 1.81  (1.18 – 2.77) | 1.75  (1.13 – 2.69) |
| **Biliary** | 1.22  (0.69 – 2.17) | 1.15  (0.58 – 1.87) |
| **Blood vessel** | 0.69  (0.33 – 1.41) | 0.64  (0.31 – 1.33) |
| **Cardiomyopathy** | 1.53  (1.15 – 2.04) | 1.45  (1.08 – 1.94) |
| **Integumentary** | 0.96  (0.57 – 1.61) | 0.89  (0.52 – 1.52) |
| **Intest/ panc** | 1.10  (0.87 – 1.40) | 1.10  (0.86 – 1.40) |
| **Liver** | 0.84  (0.57 – 1.26) | 0.89  (0.59 – 1.33) |
| **Lung** | 1.24  (0.96– 1.61) | 1.19  (0.91 – 1.55) |
| **Reproductive** | 0.96  (0.33 – 2.75) | 0.85  (0.29 – 2.51) |
| **Skeletal** | 1.18  (0.93 – 1.49) | 1.17  (0.92 – 1.48) |
| **Systemic** | 1.18  (0.57 – 2.46) | 1.23  (0.57 – 2.61) |
| **Urinary** | 1.82  (1.00 – 3.29) | 1.71  (0.93 – 3.14) |
| **Valve** | 1.17  (0.80 – 1.71) | 1.09  (0.74 – 1.62) |

Table 23| Fully adjusted odds ratios for the development of each fibrotic condition in the three years post index date. Fully adjusted analyses included adjustment for age, sex, smoking status, deprivation, number of fibrotic conditions at baseline and hypertension severity. This sensitivity analysis included diabetes duration as a covariate, where findings differ these are made apparent with red text.

Table 24 | Fully adjusted odds ratios for the development of each fibrotic condition in the three years post index date. Fully adjusted analyses included adjustment for age, sex, smoking status, deprivation, number of fibrotic conditions at baseline and hypertension severity. This sensitivity analysis included diabetes duration as a covariate, where findings differ these are made apparent with red text.

|  | **ACR elevated/ eGFR norm** | |  | **ACR elevated/ eGFR decreased** | |  | **ACR norm / eGFR decreased** | |
| --- | --- | --- | --- | --- | --- | --- | --- | --- |
| **Fibrotic condition** | **Fully adjusted**  **OR (99% CI)** | **Fully adjusted**  **OR + diabetes duration (99% CI)** |  | **Fully adjusted**  **OR (99% CI)** | **Fully adjusted**  **OR + diabetes duration (99% CI)** |  | **Fully adjusted**  **OR (99% CI)** | **Fully adjusted OR + diabetes duration (99% CI)** |
|  |  |  |  |  |  |  |  |  |
| **Atherosclerosis** | 1.75  (0.95 - 3.21) | 1.75  (0.95 – 3.21) |  | 1.85  (0.96 - 3.54) | 1.81  (0.94 – 3.47) |  | 1.08  (0.47 - 2.52) | 1.06  (0.46 – 2.48) |
| **Biliary** | 0.72  (0.29 - 1.84) | 0.72  (0.28 – 1.83) |  | 1.01  (0.40 - 2.54) | 1.00  (0.40 – 2.51) |  | 0.39  (0.09 - 1.65) | 0.40  (0.09 – 1.66) |
| **Blood vessel** | 0.59  (0.18 - 1.94) | 0.59  (0.18 – 1.95) |  | 0.54  (0.16 - 1.90) | 0.53  (0.15 – 1.85) |  | 0.63  (0.18 - 2.21) | 0.62  (0.18 – 2.20) |
| **Cardiomyopathy** | 1.29  (0.87 - 1.91) | 1.29  (0.87 – 1.91) |  | 1.21  (0.74 - 1.97) | 1.19  (0.73 – 1.93) |  | 1.31  (0.78 - 2.23) | 1.32  (0.78 – 2.24) |
| **Integumentary** | 0.78  (0.32 - 1.89) | 0.72  (0.29 – 1.80) |  | 1.19  (0.48 - 2.95) | 1.15  (0.46 – 2.88) |  | 1.58  (0.62 - 4.07) | 1.63  (0.63 – 4.21) |
| **Intest/ panc** | 1.21  (0.87 - 1.68) | 1.20  (0.86 – 1.67) |  | 1.18  (0.80 - 1.68) | 1.18  (0.80 – 1.75) |  | 0.90  (0.57 - 1.43) | 0.90  (0.57 – 1.43) |
| **Liver** | 1.25  (0.73 - 2.14) | 1.27  (0.74 – 2.18) |  | 0.67  (0.31 - 1.46) | 0.68  (0.31 – 1.48) |  | 0.90  (0.40 – 2.04) | 0.89  (0.39 – 2.02) |
| **Lung** | 1.95  (1.33 - 2.85) | 1.93  (1.32 – 2.83) |  | 2.31  (1.56 - 3.42) | 2.27  (1.53 – 3.37) |  | 1.09  (0.67 – 1.79) | 1.09  (0.67 – 1.79) |
| **Reproductive** | 1.23  (0.25 - 6.08) | 1.25  (0.25 – 6.25) |  | 1.19  (0.17 - 8.15) | 1.20  (0.17 – 8.31) |  | 0.65  (0.04 - 11.35) | 0.62  (0.04 – 10.75) |
| **Skeletal** | 1.13  (0.81 - 1.58) | 1.13  (0.81 – 1.57) |  | 0.99  (0.66 - 1.48) | 0.98  (0.65 – 1.47) |  | 1.20  (0.80 - 1.82) | 1.22  (0.81 – 1.85) |
| **Systemic** | 1.66  (0.58 - 4.70) | 1.54  (0.53 – 4.50) |  | 2.24  (0.73 - 6.90) | 2.27  (0.73 – 7.02) |  | 2.27  (0.61 - 8.50) | 2.22  (0.89 – 8.43) |
| **Urinary** | 3.55  (1.57 - 7.99) | 3.67  (1.63 – 8.28) |  | 4.40  (1.67 - 11.61) | 4.39  (1.66 – 11.55) |  | 2.41  (0.71 - 8.14) | 2.48  (0.73 – 8.44) |
| **Valve** | 1.53  (0.89 - 2.63) | 1.52  (0.88 – 2.62) |  | 1.46  (0.84 - 2.54) | 1.42  (0.82 – 2.48) |  | 0.85  (0.43 - 1.71) | 0.85  (0.42 – 1.70) |

Table 25| Fully adjusted odds ratios for the development of each fibrotic condition in the three years post index date. Fully adjusted analyses included adjustment for age, sex, smoking status, deprivation, number of fibrotic conditions at baseline and hypertension severity. This sensitivity analysis included diabetes duration as a covariate, where findings differ these are made apparent with red text.

|  | **Persistent ACR elevated/ eGFR norm** | |  | **Persistent ACR elevated/ Persistent eGFR decreased** | |  | **ACR norm / Persistent eGFR decreased** | |
| --- | --- | --- | --- | --- | --- | --- | --- | --- |
| **Fibrotic condition** | **Fully adjusted**  **OR (99% CI)** | **Fully adjusted**  **OR + diabetes duration (99% CI)** |  | **Fully adjusted**  **OR (99% CI)** | **Fully adjusted**  **OR + diabetes duration (99% CI)** |  | **Fully adjusted**  **OR (99% CI)** | **Fully adjusted**  **OR + diabetes duration (99% CI)** |
|  |  |  |  |  |  |  |  |  |
| **Atherosclerosis** | 1.33  (0.63 - 2.78) | 1.30  (0.62 – 2.73) |  | 1.65  (0.81 - 3.38) | 1.60  (0.78 – 3.28) |  | 1.00  (0.42 - 2.41) | 0.98  (0.41 – 2.37) |
| **Biliary** | 0.67  (0.20 - 2.64) | 0.66  (0.20 – 2.23) |  | 0.89  (0.30 - 2.64) | 0.90  (0.30 – 2.67) |  | 0.70  (0.19 - 2.52) | 0.70  (0.19 – 2.52) |
| **Blood vessel** | 0.77  (0.21 - 2.86) | 0.78  (0.21 – 2.92) |  | 0.47  (0.10 - 2.13) | 0.45  (0.10 – 2.05) |  | 1.15  (0.36 - 3.68) | 1.13  (0.35 – 3.61) |
| **Cardiomyopathy** | 1.34  (0.86 - 2.09) | 1.34  (0.85 – 2.11) |  | 1.07  (0.62 - 1.82) | 1.04  (0.61 – 1.77) |  | 1.19  (0.69 - 2.06) | 1.18  (0.68 – 2.05) |
| **Integumentary** | 0.92  (0.33 - 2.55) | 0.99  (0.35 – 2.77) |  | 1.08  (0.39 - 2.98) | 1.06  (0.38 – 2.96) |  | 1.49  (0.39 - 3.99) | 1.64  (0.61 – 4.41) |
| **Intest/ panc** | 0.90  (0.61 - 1.34) | 0.89  (0.60 – 1.33) |  | 0.80  (0.52 - 1.25) | 0.81  (0.52 – 1.25) |  | 0.67  (0.41 - 1.07) | 0.66  (0.41 – 1.07) |
| **Liver** | 1.39  (0.76 - 2.53) | 1.40  (0.77 – 2.56) |  | 0.80  (0.36 - 1.76) | 0.80  (0.36 – 1.78) |  | 0.53  (0.20 - 2.53) | 0.53  (0.20 – 1.41) |
| **Lung** | 2.07  (1.35 - 3.17) | 2.05  (1.34 – 3.15) |  | 2.18  (1.43 - 3.17) | 2.14  (1.40 – 3.27) |  | 1.19  (0.73 - 1.94) | 1.20  (0.73 – 1.95) |
| **Reproductive** | 0.46  (0.03 - 7.59) | 0.45  (0.03 – 7.40) |  | 2.25  (0.35 - 14.55) | 2.22  (0.34 – 14.47) |  | 0.68  (0.04 - 12.88) | 0.62  (0.03 – 11.85) |
| **Skeletal** | 1.18  (0.80 - 1.74) | 1.18  (0.80 – 1.74) |  | 1.00  (0.65 - 1.56) | 1.01  (0.65 – 1.56) |  | 1.33  (0.87 - 2.03) | 1.36  (0.89 – 2.08) |
| **Systemic** | 1.63  (0.49 - 5.46) | 1.83  (0.53 – 6.28) |  | 1.66  (0.45 - 6.06) | 1.77  (0.47 – 6.66) |  | 1.82  (0.43 - 7.67) | 1.92  (0.45 – 8.28) |
| **Urinary** | 2.37  (0.94 - 5.95) | 2.49  (0.99 – 6.28) |  | 2.88  (1.04 - 7.95) | 2.83  (1.02 – 7.82) |  | 1.34  (0.37 - 5.95) | 1.39  (0.38 – 5.08) |
| **Valve** | 1.73  (0.94 - 3.17) | 1.75  (0.95 – 3.21) |  | 1.52  (0.83 - 2.80) | 1.49  (0.81 – 2.75) |  | 1.20  (0.62 - 2.35) | 1.19  (0.61 – 2.33) |

Table 26 | Fully adjusted odds ratios for the development of each fibrotic condition in the three years post index date. Fully adjusted analyses included adjustment for age, sex, smoking status, deprivation, number of fibrotic conditions at baseline and hypertension severity. This sensitivity analysis included body mass index (BMI) as a covariate, where findings differ these are made apparent with red text.

| **Microvascular complications** | | |
| --- | --- | --- |
| **Fibrotic condition** | **Fully adjusted**  **OR (99% CI)** | **Fully adjusted**  **OR + BMI (99% CI)** |
|  |  |  |
| **Atherosclerosis** | 1.81  (1.18 – 2.77) | 1.83  (1.17 – 2.84) |
| **Biliary** | 1.22  (0.69 – 2.17) | 1.23  (0.68 – 2.22) |
| **Blood vessel** | 0.69  (0.33 – 1.41) | 0.76  (0.35 – 1.66) |
| **Cardiomyopathy** | 1.53  (1.15 – 2.04) | 1.58  (1.17 – 2.14) |
| **Integumentary** | 0.96  (0.57 – 1.61) | 0.92  (0.53 – 1.58) |
| **Intest/ panc** | 1.10  (0.87 – 1.40) | 1.11  (0.87 – 1.43) |
| **Liver** | 0.84  (0.57 – 1.26) | 0.91  (0.59 – 1.39) |
| **Lung** | 1.24  (0.96– 1.61) | 1.24  (0.95 – 1.62) |
| **Reproductive** | 0.96  (0.33 – 2.75) | 1.20  (0.39 – 3.70) |
| **Skeletal** | 1.18  (0.93 – 1.49) | 1.20  (0.94 – 1.53) |
| **Systemic** | 1.18  (0.57 – 2.46) | 1.04  (0.49 – 2.23) |
| **Urinary** | 1.82  (1.00 – 3.29) | 1.68  (0.89 – 3.19) |
| **Valve** | 1.17  (0.80 – 1.71) | 1.15  (0.76 – 1.72) |

Table 27 | Fully adjusted odds ratios for the development of each fibrotic condition in the three years post index date. Fully adjusted analyses included adjustment for age, sex, smoking status, deprivation, number of fibrotic conditions at baseline and hypertension severity. This sensitivity analysis included body mass index (BMI) as a covariate, where findings differ these are made apparent with red text.

|  | **ACR elevated/ eGFR norm** | |  | **ACR elevated/ eGFR decreased** | |  | **ACR norm / eGFR decreased** | |
| --- | --- | --- | --- | --- | --- | --- | --- | --- |
| **Fibrotic condition** | **Fully adjusted**  **OR (99% CI)** | **Fully adjusted**  **OR + BMI (99% CI)** |  | **Fully adjusted**  **OR (99% CI)** | **Fully adjusted**  **OR + BMI (99% CI)** |  | **Fully adjusted**  **OR (99% CI)** | **Fully adjusted**  **OR + BMI (99% CI)** |
|  |  |  |  |  |  |  |  |  |
| **Atherosclerosis** | 1.75  (0.95 - 3.21) | 1.80  (0.97 – 3.35) |  | 1.85  (0.96 - 3.54) | 1.79  (0.91 – 3.54) |  | 1.08  (0.47 - 2.52) | 1.02  (0.42 – 2.52) |
| **Biliary** | 0.72  (0.29 - 1.84) | 0.75  (0.29 – 1.91) |  | 1.01  (0.40 - 2.54) | 0.93  (0.35 – 2.48) |  | 0.39  (0.09 - 1.65) | 0.45  (0.11 – 1.88) |
| **Blood vessel** | 0.59  (0.18 - 1.94) | 0.66  (0.20 – 2.19) |  | 0.54  (0.16 - 1.90) | 0.41  (0.09 – 1.81) |  | 0.63  (0.18 - 2.21) | 0.60  (0.15 – 2.37) |
| **Cardiomyopathy** | 1.29  (0.87 - 1.91) | 1.28  (0.85 – 2.00) |  | 1.21  (0.74 - 1.97) | 1.20  (0.72 – 2.00) |  | 1.31  (0.78 - 2.23) | 1.16  (0.65 – 2.06) |
| **Integumentary** | 0.78  (0.32 - 1.89) | 0.75  (0.30 – 1.88) |  | 1.19  (0.48 - 2.95) | 1.19  (0.47 – 3.04) |  | 1.58  (0.62 - 4.07) | 1.73  (0.66 – 4.52) |
| **Intest/ panc** | 1.21  (0.87 - 1.68) | 1.16  (0.82 – 1.65) |  | 1.18  (0.80 - 1.68) | 1.21  (0.81 – 1.83) |  | 0.90  (0.57 - 1.43) | 0.96  (0.60 – 1.55) |
| **Liver** | 1.25  (0.73 - 2.14) | 1.35  (0.76 – 2.42) |  | 0.67  (0.31 - 1.46) | 0.79  (0.35 – 1.77) |  | 0.90  (0.40 – 2.04) | 1.02  (0.42 – 2.50) |
| **Lung** | 1.95  (1.33 - 2.85) | 1.89  (1.27 – 2.79) |  | 2.31  (1.56 - 3.42) | 2.31  (1.54 – 3.47) |  | 1.09  (0.67 - 1.79) | 1.05  (0.63 – 1.76) |
| **Reproductive** | 1.23  (0.25 - 6.08) | 1.06  (0.18 – 6.46) |  | 1.19  (0.17 - 8.15) | 1.57  (0.22 – 11.41) |  | 0.65  (0.04 - 11.35) | 0.92  (0.05 – 16.33) |
| **Skeletal** | 1.13  (0.81 - 1.58) | 1.11  (0.79 – 1.57) |  | 0.99  (0.66 - 1.48) | 0.91  (0.60 – 1.40) |  | 1.20  (0.80 - 1.82) | 1.16  (0.75 – 1.79) |
| **Systemic** | 1.66  (0.58 - 4.70) | 1.42  (0.47 – 4.29) |  | 2.24  (0.73 - 6.90) | 2.41  (0.77 – 7.59) |  | 2.27  (0.61 - 8.50) | 2.24  (0.55 – 9.17) |
| **Urinary** | 3.55  (1.57 - 7.99) | 4.05  (1.63 – 10.04) |  | 4.40  (1.67 - 11.61) | 4.96  (1.69 – 14.53) |  | 2.41  (0.71 - 8.14) | 2.78  (0.72 – 10.66) |
| **Valve** | 1.53  (0.89 - 2.63) | 1.48  (0.84 – 2.59) |  | 1.46  (0.84 - 2.54) | 1.37  (0.77 – 2.43) |  | 0.85  (0.43 - 1.71) | 0.84  (0.41 – 1.72) |

Table 28 | Fully adjusted odds ratios for the development of each fibrotic condition in the three years post index date. Fully adjusted analyses included adjustment for age, sex, smoking status, deprivation, number of fibrotic conditions at baseline and hypertension severity. This sensitivity analysis included body mass index (BMI) as a covariate, where findings differ these are made apparent with red text.

|  | **Persistent ACR elevated/ eGFR norm** | |  | **Persistent ACR elevated/ Persistent eGFR decreased** | |  | **ACR norm / Persistent eGFR decreased** | |
| --- | --- | --- | --- | --- | --- | --- | --- | --- |
| **Fibrotic condition** | **Fully adjusted**  **OR (99% CI)** | **Fully adjusted**  **OR + BMI (99% CI)** |  | **Fully adjusted**  **OR (99% CI)** | **Fully adjusted**  **OR + BMI (99% CI)** |  | **Fully adjusted**  **OR (99% CI)** | **Fully adjusted**  **OR + BMI (99% CI)** |
|  |  |  |  |  |  |  |  |  |
| **Atherosclerosis** | 1.33  (0.63 - 2.78) | 1.43  (0.67 – 3.05) |  | 1.65  (0.81 - 3.38) | 1.71  (0.80 – 3.64) |  | 1.00  (0.42 - 2.41) | 1.15  (0.47 – 2.82) |
| **Biliary** | 0.67  (0.20 - 2.64) | 0.66  (0.20 – 2.23) |  | 0.89  (0.30 - 2.64) | 0.83  (0.27 – 2.57) |  | 0.70  (0.19 - 2.52) | 0.72  (0.20 – 2.65) |
| **Blood vessel** | 0.77  (0.21 - 2.86) | 0.82  (0.22 – 3.10) |  | 0.47  (0.10 - 2.13) | 0.42  (0.08 – 2.31) |  | 1.15  (0.36 - 3.68) | 1.02  (0.28 – 3.64) |
| **Cardiomyopathy** | 1.34  (0.86 - 2.09) | 1.34  (0.85 – 2.14) |  | 1.07  (0.62 - 1.82) | 1.08  (0.61 – 1.89) |  | 1.19  (0.69 - 2.06) | 1.10  (0.60 – 2.00) |
| **Integumentary** | 0.92  (0.33 - 2.55) | 0.89  (0.30 – 2.61) |  | 1.08  (0.39 - 2.98) | 0.98  (0.33 – 2.88) |  | 1.49  (0.39 - 3.99) | 1.60  (0.58 – 4.38) |
| **Intest/ panc** | 0.90  (0.61 - 1.34) | 0.90  (0.60 – 1.36) |  | 0.80  (0.52 - 1.25) | 0.80  (0.50 – 1.26) |  | 0.67  (0.41 - 1.07) | 0.69  (0.42 – 1.13) |
| **Liver** | 1.39  (0.76 - 2.53) | 1.28  (0.66 – 2.47) |  | 0.80  (0.36 - 1.76) | 0.86  (0.37 – 1.97) |  | 0.53  (0.20 - 2.53) | 0.54  (0.19 – 1.54) |
| **Lung** | 2.07  (1.35 - 3.17) | 2.11  (1.37 – 3.28) |  | 2.18  (1.43 - 3.17) | 2.22  (1.43 – 3.43) |  | 1.19  (0.73 - 1.94) | 1.22  (0.74 – 2.02) |
| **Reproductive** | 0.46  (0.03 - 7.59) | 0.52  (0.03 – 8.80) |  | 2.25  (0.35 - 14.55) | 2.97  (0.42 – 20.89) |  | 0.68  (0.04 - 12.88) | 1.01  (0.05 – 20.11) |
| **Skeletal** | 1.18  (0.80 - 1.74) | 1.15  (0.77 – 1.72) |  | 1.00  (0.65 - 1.56) | 0.84  (0.52 – 1.34) |  | 1.33  (0.87 - 2.03) | 1.30  (0.84 – 2.02) |
| **Systemic** | 1.63  (0.49 - 5.46) | 1.25  (0.33 – 4.77) |  | 1.66  (0.45 - 6.06) | 1.47  (0.37 – 5.77) |  | 1.82  (0.43 - 7.67) | 1.97  (0.45 – 8.70) |
| **Urinary** | 2.37  (0.94 - 5.95) | 2.25  (0.83 – 6.09) |  | 2.88  (1.04 - 7.95) | 2.47  (0.83 – 7.36) |  | 1.34  (0.37 - 5.95) | 0.70  (0.13 – 3.80) |
| **Valve** | 1.73  (0.94 - 3.17) | 1.90  (1.02 – 3.54) |  | 1.52  (0.83 - 2.80) | 1.45  (0.77 – 2.75) |  | 1.20  (0.62 - 2.35) | 1.19  (0.59 – 2.39) |

## **Type 2 diabetes prevalence and odds ratios**

Table 29 | Prevalence of fibrotic conditions per exposure group, based upon evidence of microvascular complications.

|  | **Whole cohort %**  **(95% CI)** | **No evidence of microvascular complications % (95% CI)** | **Microvascular complications % (95% CI)** |
| --- | --- | --- | --- |
| **Atherosclerosis** | 4.24  (4.18 – 4.29) | 3.89  (3.84 – 3.95) | 10.60  (10.22 – 10.98) |
| **Biliary** | 3.69  (3.64 – 3.75) | 3.55  (3.50 – 3.61) | 6.27  (5.97 – 6.57) |
| **Blood vessel** | 2.25  (2.21 – 2.29) | 2.15  (2.11 – 2.19) | 4.05  (3.80 – 4.29) |
| **Cardiomyopathy** | 18.13  (18.03 – 18.24) | 17.43  (17.33 – 17.54) | 30.95  (30.38 – 31.53) |
| **Integumentary** | 5.14  (5.08 – 5.20) | 4.88  (4.82 – 4.95) | 9.83  (9.46 – 10.19) |
| **Intest/ panc** | 26.36  (26.24 – 26.48) | 25.59  (25.46 – 25.71) | 40.32  (39.71 – 40.92) |
| **Liver** | 6.44  (6.38 – 6.51) | 6.40  (6.34 – 6.47) | 7.00  (6.69 – 7.32) |
| **Reproductive** | 2.40  (2.34 – 2.46) | 2.45  (2.41 – 2.49) | 1.32  (1.11 – 1.53) |
| **Lung** | 12.08  (11.99 – 12.17) | 11.18  (11.09 – 11.26) | 29.02  (28.46 – 29.58) |
| **Skeletal** | 26.82  (26.70 – 26.94) | 25.81  (25.69 – 25.94) | 45.27  (44.66 – 45.89) |
| **Systemic** | 1.94  (1.90 – 1.98) | 1.73  (1.69 – 1.77) | 5.92  (5.64 – 6.22) |
| **Urinary** | 4.09  (4.03 – 4.14) | 3.42  (3.37 – 3.47) | 16.67  (16.20 – 17.12) |
| **Valve** | 6.75  (6.68 – 6.82) | 6.15  (6.08 – 6.22) | 17.94  (17.46 – 18.41) |

Table 30 | *Crude and fully adjusted odds ratios for the development of each fibrotic condition in the three years post index date. Fully adjusted analyses included adjustment for age, sex, smoking status, deprivation, number of fibrotic conditions at baseline and hypertension severity.*

|  | **Microvascular complications** | | | |
| --- | --- | --- | --- | --- |
|  | **Crude OR (99% CI)** | **Fully adjusted**  **OR (99% CI)** | | **p-value** |
| **Atherosclerosis** | 2.81  (2.54 – 3.11) | | 1.28  (1.15 – 1.42) | <0.001 |
| **Biliary** | 1.99  (1.75 – 2.26) | | 0.95  (0.84 – 1.09) | 0.370 |
| **Blood vessel** | 1.98  (1.67 – 2.36) | | 1.08  (0.90 – 1.29) | 0.265 |
| **Cardiomyopathy** | 1.89  (1.75 – 2.05) | | 1.11  (1.02 – 1.20) | 0.001 |
| **Integumentary** | 2.32  (2.05 – 2.62) | | 1.29  (1.13 – 1.46) | <0.001 |
| **Intest/ panc** | 1.88  (1.77 – 1.99) | | 1.01  (0.95 – 1.07) | 0.787 |
| **Liver** | 1.15  (1.02 – 1.28) | | 0.83  (0.74 – 0.93) | <0.001 |
| **Lung** | 3.42  (3.24 – 3.61) | | 1.41  (1.33 – 1.49) | <0.001 |
| **Reproductive** | 1.47  (1.13 – 1.89) | | 1.15  (0.88 – 1.50) | 0.190 |
| **Skeletal** | 2.17  (2.05 – 2.29) | | 1.14  (1.07 – 1.20) | <0.001 |
| **Systemic** | 3.19  (2.74 – 3.70) | | 1.53  (1.30 – 1.79) | <0.001 |
| **Urinary** | 4.12  (3.68 – 4.62) | | 2.23  (1.98 – 2.52) | <0.001 |
| **Valve** | 3.40  (3.15 – 3.66) | | 1.35  (1.24 – 1.46) | <0.001 |

Table 31| Prevalence of fibrotic conditions per exposure group defined using measures of albumin to creatinine ratio (ACR) and glomerular filtration rate (eGFR). Elevated ACR and eGFR was defined as ACR ≥3 mg/mmol, eGFR <60 mL/min/1.73 m^2^ respectively.

|  | **Whole cohort *** | **ACR norm / eGFR norm** | **ACR elevated/ eGFR norm** | **ACR elevated/ eGFR decreased** | **ACR norm / eGFR decreased** |
| --- | --- | --- | --- | --- | --- |
| **Atherosclerosis** | 4.53  (4.46 – 4.60) | 3.02  (2.95 – 3.10) | 5.80  (5.61 – 5.99) | 10.57  (10.22 – 10.93) | 6.46  (6.22 – 6.71) |
| **Biliary** | 3.84  (3.78 – 3.91) | 3.19  (3.12 – 3.27) | 3.76  (3.61 – 3.92) | 6.19  (5.91 – 6.46) | 5.85  (5.61 – 6.09) |
| **Blood vessel** | 2.31  (2.26 – 2.37) | 1.82  (1.77 – 1.88) | 2.34  (2.22 – 2.47) | 4.09  (3.86 – 4.31) | 3.70  (3.50 – 3.89) |
| **Cardiomyopathy** | 19.26  (19.13 – 19.40) | 16.18  (16.02 – 16.34) | 21.03  (20.70 – 21.36) | 30.71  (30.18 – 31.25) | 25.18  (24.74 – 25.62) |
| **Integumentary** | 5.40  (5.33 – 5.48) | 4.44  (4.35 – 4.53) | 5.38  (5.20 – 5.57) | 9.69  (9.35 – 10.03) | 7.59  (7.33 – 7.86) |
| **Intest/ panc** | 27.54  (27.38 – 27.69) | 24.07  (23.89 – 24.25) | 27.90  (27.53 – 28.26) | 40.05  (39.49 – 40.62) | 36.92  (36.44 – 37.41) |
| **Liver** | 6.50  (6.42 – 6.58) | 6.23  (6.13 – 6.33) | 7.96  (7.74 – 8.18) | 7.09  (6.79 – 7.39 | 5.31  (5.08 – 5.53) |
| **Reproductive** | 2.34  (2.26 – 2.42) | 2.72  (2.61 – 2.83) | 2.38  (2.19 – 2.57) | 1.40  (1.20 – 1.60) | 1.25  (1.10 – 1.40) |
| **Lung** | 12.74  (12.63 – 12.85) | 8.68  (8.56 – 8.80) | 14.61  (14.32 – 14.90) | 29.06  (28.84 – 29.58) | 20.28  (19.87 – 20.68) |
| **Skeletal** | 28.39  (28.24 – 28.54) | 23.83  (23.64 – 24.01) | 29.11  (28.74 – 29.48) | 44.90  (44.33 – 45.47) | 40.40  (39.90 – 40.89) |
| **Systemic** | 2.07  (2.03 – 2.12) | 1.26  (1.21 – 1.31) | 2.43  (2.31 – 2.56) | 5.87  (5.60 – 6.14) | 3.20  (3.02 – 3.37) |
| **Urinary** | 4.53  (4.46 – 4.60) | 2.17  (2.11 – 2.24) | 4.79  (4.62 – 4.96) | 16.46  (16.03 – 16.89) | 8.26  (7.99 – 8.54) |
| **Valve** | 7.48  (7.40 – 7.57) | 4.93  (4.84 – 5.02) | 8.22  (8.00 – 8.45) | 17.67  (17.23 – 18.11) | 1.96  (12.62 – 13.30 |

|  | 1. **ACR elevated/ eGFR norm** | | | 1. **ACR elevated/ eGFR decreased** | | | 1. **ACR norm / eGFR decreased** | | |
| --- | --- | --- | --- | --- | --- | --- | --- | --- | --- |
| **Fibrotic condition** | **Crude OR (99% CI)** | **Fully adjusted**  **OR (99% CI)** | **p-value** | **Crude OR (99% CI)** | **Fully adjusted**  **OR (99% CI)** | **p-value** | **Crude OR (99% CI)** | **Fully adjusted**  **OR (99% CI)** | **p-value** |
| **Atherosclerosis** | 2.00  (1.81 – 2.21) | 1.56  (1.41 – 1.73) | <0.001 | 3.69  (3.32 – 4.10) | 1.63  (1.45 – 1.83) | <0.001 | 2.04  (1.82 – 2.29) | 1.10  (0.97 – 1.25) | 0.041 |
| **Biliary** | 1.19  (1.05 – 1.35) | 0.93  (0.82 –1.05) | 0.136 | 2.16  (1.89 – 2.46) | 0.95  (0.82 – 1.09) | 0.331 | 2.06  (1.82 – 2.33) | 1.08  (0.95 – 1.23) | 0.126 |
| **Blood vessel** | 1.33  (1.12 – 1.57) | 1.13  (0.95 – 1.34) | 0.067 | 2.41  (2.02 – 2.88) | 1.27  (1.05 – 1.54) | 0.001 | 2.32  (1.98 – 2.73) | 1.37  (1.15 – 1.63) | <0.001 |
| **Cardiomyopathy** | 1.47  (1.38 – 1.56) | 1.24  (1.16 – 1.32) | <0.001 | 2.09  (1.94 – 2.26) | 1.26  (1.15 – 1.37) | <0.001 | 1.41  (1.30 – 1.52) | 1.05  (0.97 – 1.15) | 0.111 |
| **Integumentary** | 1.34  (1.18 – 1.51) | 1.11  (0.98 – 1.26) | 0.033 | 2.64  (2.33 – 3.00) | 1.43  (1.25 – 1.65) | <0.001 | 1.73  (1.52 – 1.98) | 1.12  (0.98 – 1.30) | 0.033 |
| **Intest/ panc** | 1.29  (1.23 – 1.36) | 1.09  (1.04 – 1.15) | <0.001 | 2.04  (1.92 – 2.16) | 1.06  (0.99 – 1.13) | 0.025 | 1.72  (1.63 – 1.81) | 1.02  (0.96 – 1.08) | 0.474 |
| **Liver** | 1.26  (1.17 – 1.37) | 1.07  (0.99 – 1.17) | 0.027 | 1.19  (1.06 – 1.32) | 0.90  (0.80 – 1.02) | 0.030 | 0.91  (0.82 – 1.02) | 0.85  (0.76 – 0.96) | <0.001 |
| **Lung** | 1.94  (1.84 – 2.06) | 1.57  (1.48 – 1.66) | <0.001 | 4.82  (4.56 – 5.10) | 1.84  (1.73 – 1.96) | <0.001 | 2.96  (2.79 – 3.13) | 1.27  (1.19 – 1.35) | <0.001 |
| **Reproductive** | 1.41  (1.15 – 1.72) | 1.26  (1.02 – 1.54) | 0.004 | 1.55  (1.20 – 2.00) | 1.22  (0.93 – 1.62) | 0.062 | 1.03  (0.79 – 1.36) | 1.04  (0.77 – 1.38) | 0.758 |
| **Skeletal** | 1.29  (1.23 – 1.35) | 1.10  (1.05 – 1.16) | <0.001 | 2.38  (2.25 – 2.52) | 1.21  (1.14 – 1.28) | <0.001 | 2.07  (1.97 – 2.18) | 1.15  (1.09 – 1.22) | <0.001 |
| **Systemic** | 2.01  (1.71 – 2.37) | 1.49  (1.27 – 1.76) | <0.001 | 4.27  (3.63 – 5.01) | 2.02  (1.69 – 2.42) | <0.001 | 2.25  (1.87 – 2.69) | 1.45  (1.19 – 1.76) | <0.001 |
| **Urinary** | 2.50  (2.20 – 2.84) | 2.11  (1.86 – 2.41) | <0.001 | 6.04  (5.33 – 6.85) | 3.48  (3.03 – 4.00) | <0.001 | 2.59  (2.23 – 3.00) | 1.75  (1.49 – 2.05) | <0.001 |
| **Valve** | 1.81  (1.67 – 1.96) | 1.37  (1.26 – 1.48) | <0.001 | 4.05  (3.74 – 4.39) | 1.44  (1.32 – 1.57) | <0.001 | 2.60  (2.39 – 2.83) | 1.11  (1.02 – 1.22) | 0.002 |

*Table 32| Crude and fully adjusted odds ratios for the development of each fibrotic condition in the three years post index date. Fully adjusted analyses included adjustment for age, sex, smoking status, deprivation, number of fibrotic conditions at baseline and hypertension severity.*

Table 33 | Prevalence of fibrotic conditions per exposure group defined using multiple measures of albumin to creatinine ratio (ACR) and glomerular filtration rate (eGFR). Elevated ACR and eGFR was defined as ACR ≥3 mg/mmol, eGFR <60 mL/min/1.73 m^2^ respectively.

|  | **Whole cohort *** | **ACR norm / eGFR norm** | **Persistent ACR elevated/ eGFR norm** | **Persistent ACR elevated/ Persistent eGFR decreased** | **ACR norm / Persistent eGFR decreased** |
| --- | --- | --- | --- | --- | --- |
| **Atherosclerosis** | 4.81  (4.72 – 4.89) | 3.18  (3.09 – 3.26) | 6.09  (5.85 – 6.32) | 10.60  (10.22 – 10.98) | 6.76  (6.50 – 7.02) |
| **Biliary** | 3.92  (3.85 – 3.99) | 3.15  (3.07 – 3.23) | 3.77  (3.58 – 3.96) | 6.27  (5.97 – 6.57) | 5.92  (5.68 – 6.16) |
| **Blood vessel** | 2.38  (2.33 – 2.44) | 1.84  (1.78 – 1.91) | 2.30  (2.15 – 2.45) | 4.05  (3.80 – 4.29) | 3.77  (3.57 – 3.96) |
| **Cardiomyopathy** | 19.92  (19.77 – 20.07) | 16.58  (16.41 – 16.76) | 21.83  (21.42 – 22.24) | 30.95  (30.38 – 31.53) | 25.26  (24.82 – 25.71) |
| **Integumentary** | 5.59  (5.50 – 5.68) | 4.51  (4.41 – 4.61) | 5.32  (5.10 – 5.54) | 9.83  (9.46 – 10.19) | 7.82  (7.55 – 8.09) |
| **Intest/ panc** | 28.22  (28.05 – 28.39) | 24.32  (24.12 – 24.53) | 28.26  (27.82 – 28.71) | 40.32  (39.71 – 40.92) | 37.35  (36.86 – 37.85) |
| **Liver** | 6.48  (6.39 – 6.58) | 6.20  (6.08 – 6.31) | 8.20  (7.93 – 8.47) | 7.00  (6.69 – 7.32) | 5.59  (5.35 – 5.82) |
| **Reproductive** | 2.22  (2.13 – 2.31) | 2.64  (2.52 – 2.76) | 2.31  (2.07 – 2.55) | 1.32  (1.11 – 1.53) | 1.21  (1.06 – 1.36) |
| **Lung** | 13.28  (13.15 – 13.41) | 8.90  (8.76 – 9.04) | 14.83  (14.48 – 15.18) | 29.02  (28.46 – 29.58) | 20.45  (20.04 – 20.86) |
| **Skeletal** | 29.34  (29.17 – 29.51) | 24.25  (24.04 – 24.46) | 29.80  (29.35 – 30.25) | 45.27  (44.66 – 45.89) | 40.75  (40.25 – 41.25) |
| **Systemic** | 2.19  (2.13 – 2.24) | 1.31  (1.25 – 1.36) | 2.48  (2.33 – 2.64) | 5.93  (5.64 – 6.22) | 3.27  (3.09 – 3.45) |
| **Urinary** | 4.83  (4.75 – 4.92) | 2.20  (2.13 – 2.27) | 4.98  (4.76 – 5.19) | 16.66  (16.20 – 17.12) | 8.40  (8.12 – 8.68) |
| **Valve** | 7.95  (7.85 – 8.06) | 5.10  (4.99 – 5.20) | 8.71  (8.43 – 8.99) | 16.87  (16.40 – 17.33) | 13.11  (12.77 – 13.45) |

|  | 1. **Persistent ACR elevated/ eGFR norm** | | | | 1. **Persistent ACR elevated/ persistent eGFR decreased** | | | | 1. **ACR norm / persistent eGFR decreased** | | |
| --- | --- | --- | --- | --- | --- | --- | --- | --- | --- | --- | --- |
| **Fibrotic condition** | **Crude OR (99% CI)** | | **Fully adjusted**  **OR (99% CI)** | **p-value** | **Crude OR**  **(99% CI)** | **Fully adjusted**  **OR (99% CI)** | | **p-value** | **Crude OR (99% CI)** | **Fully adjusted**  **OR (99% CI)** | **p-value** |
| **Atherosclerosis** | 1.95  (1.73 – 2.19) | 1.51  (1.34 – 1.70) | | <0.001 | 3.34  (2.98 – 3.75) | 1.54  (1.36 – 1.74) | <0.001 | | 2.02  (1.80 – 2.27) | 1.14  (1.00 – 1.29) | <0.001 |
| **Biliary** | 1.30  (1.13 – 1.51) | 1.02  (0.88 – 1.18) | | 0.702 | 2.26  (1.96 – 2.60) | 1.02  (0.87 – 1.19) | 0.762 | | 2.12  (1.87 – 2.40) | 1.12  (0.98 – 1.28) | 0.032 |
| **Blood vessel** | 1.26  (1.03 – 1.54) | 1.08  (0.88 – 1.32) | | 0.339 | 2.31  (1.91 – 2.79) | 1.24  (1.01 – 1.53) | 0.007 | | 2.26  (1.92 – 2.67) | 1.34  (1.11 – 1.60) | <0.001 |
| **Cardiomyopathy** | 1.54  (1.43 – 1.66) | 1.28  (1.19 – 1.38) | | <0.001 | 2.00  (1.84 – 2.18) | 1.20  (1.09 – 1.31) | <0.001 | | 1.37  (1.26 – 1.48) | 1.03  (0.94 – 1.12) | 0.422 |
| **Integumentary** | 1.27  (1.10 – 1.47) | 1.05  (0.91 – 1.22) | | 0.373 | 2.55  (2.23 – 2.92) | 1.40  (1.21 – 1.62) | <0.001 | | 1.73  (1.51 – 1.98) | 1.12  (0.96 – 1.30) | 0.051 |
| **Intest/ panc** | 1.31  (1.24 – 1.39) | 1.11  (1.05 – 1.18) | | <0.001 | 2.00  (1.88 – 2.13) | 1.06  (0.99 – 1.13) | 0.031 | | 1.71  (1.62 – 1.81) | 1.02  (0.96 – 1.09) | 0.328 |
| **Liver** | 1.28  (1.16 – 1.41) | 1.09  (0.98 – 1.20) | | 0.031 | 1.17  (1.04 – 1.32) | 0.90  (0.79 – 1.03) | 0.040 | | 0.96  (0.86 – 1.08) | 0.90  (0.80 – 1.02) | 0.025 |
| **Lung** | 1.92  (1.80 – 2.05) | 1.54  (1.44 – 1.65) | | <0.001 | 4.58  (4.31 – 4.87) | 1.77  (1.65 – 1.89) | <0.001 | | 2.86  (2.69 – 3.03) | 1.24  (1.16 – 1.32) | <0.001 |
| **Reproductive** | 1.34  (1.05 – 1.71) | 1.17  (0.91 – 1.49) | | 0.106 | 1.56  (1.19 – 2.05) | 1.21  (0.90 – 1.63) | 0.101 | | 0.99  (0.75 – 1.31) | 0.97  (0.72 – 1.31) | 0.797 |
| **Skeletal** | 1.28  (1.21 – 1.35) | 1.10  (1.04 – 1.16) | | <0.001 | 2.31  (2.18 – 2.45) | 1.21  (1.13 – 1.29) | <0.001 | | 2.01  (1.91 – 2.12) | 1.14  (1.08 – 1.21) | <0.001 |
| **Systemic** | 2.03  (1.69 – 2.44) | 1.50  (1.24 – 1.81) | | <0.001 | 3.99  (3.35 – 4.75) | 1.91  (1.58 – 2.32) | <0.001 | | 2.17  (1.80 – 2.61) | 1.37  (1.12 – 1.67) | <0.001 |
| **Urinary** | 2.43  (2.09 – 2.81) | 2.02  (1.74 – 2.35) | | <0.001 | 5.57  (4.86 – 6.39) | 3.22  (2.77 – 3.75) | <0.001 | | 2.56  (2.20 – 2.98) | 1.73  (1.47 – 2.04) | <0.001 |
| **Valve** | 1.89  (1.73 – 2.07) | 1.44  (1.31 – 1.58) | | <0.001 | 4.02  (3.69 – 4.38) | 1.48  (1.35 – 1.63) | <0.001 | | 2.50  (2.30 – 2.73) | 1.11  (1.01 – 1.21) | 0.006 |

Table 34 | Crude and fully adjusted odds ratios for the development of each fibrotic condition in the three years post index date. Fully adjusted analyses included adjustment for age, sex, smoking status, deprivation, number of fibrotic conditions at baseline and hypertension severity.

Table 35 |Prevalence of fibrotic conditions of the whole cohort and subdivided based upon control of HbA_1c_.

|  | **Whole cohort* %**  **(95% CI)** | **Controlled glucose (95% CI)** | **Glucose above target (95% CI)** |
| --- | --- | --- | --- |
| **Atherosclerosis** | 4.24  (4.18 – 4.29) | 4.03  (3.96 – 4.10) | 4.68  (4.58 – 4.79) |
| **Biliary** | 3.71  (3.66 – 3.77) | 3.75  (3.68 – 3.81) | 3.66  (3.57 – 3.76) |
| **Blood vessel** | 2.25  (2.21 – 2.29) | 2.25  (2.20 – 2.30) | 2.26  (2.18 – 2.33) |
| **Cardiomyopathy** | 18.28  (18.17 – 18.38) | 17.53  (17.40 – 17.66) | 19.90  (19.70 – 20.10) |
| **Integumentary** | 5.17  (5.11 – 5.23) | 5.19  (5.12 – 5.27) | 5.15  (5.04 – 5.26) |
| **Intest/ panc** | 26.58  (26.46 – 26.70) | 27.13  (26.98 – 27.29) | 25.54  (25.32 – 25.75) |
| **Liver** | 6.44  (6.37 – 6.50) | 5.94  (5.86 – 6.02) | 7.49  (7.36 – 7.62) |
| **Reproductive** | 2.40  (2.34 – 2.46) | 2.29  (2.21 – 2.37) | 2.66  (2.54 – 2.78) |
| **Lung** | 12.11  (12.02 – 12.20) | 12.21  (12.10 – 12.32) | 11.95  (11.79 – 12.11) |
| **Skeletal** | 27.11  (26.98 – 27.23) | 27.95  (27.80 – 28.11) | 25.46  (25.24 – 25.67) |
| **Systemic** | 1.94  (1.90 – 1.98) | 1.90  (1.85 – 1.94) | 2.05  (1.98 – 2.12) |
| **Urinary** | 32.40  (32.27 – 32.53) | 32.39  (32.26 – 32.52) | 32.58  (31.93 – 33.23) |
| **Valve** | 6.81  (6.74 – 6.88) | 7.04  (6.95 – 7.12) | 6.36  (6.24 – 6.48) |

*Table 36| Crude and fully adjusted odds ratios for the development of each fibrotic condition in the three years post index date. Fully adjusted analyses included adjustment for age, sex, smoking status, deprivation, number of fibrotic conditions at baseline and hypertension severity.*

|  | **Glucose above target** | | |
| --- | --- | --- | --- |
|  | **Crude OR**  **(99% CI)** | **Fully adjusted**  **OR (99% CI)** | **p-value** |
| **Atherosclerosis** | 1.28  (1.20 – 1.37) | 1.45  (1.36 – 1.56) | <0.001 |
| **Biliary** | 0.94  (0.86 – 1.02) | 1.01  (0.93 – 1.10) | 0.652 |
| **Blood vessel** | 1.04  (0.93 – 1.15) | 1.16  (1.04 – 1.30) | <0.001 |
| **Cardiomyopathy** | 1.25  (1.20 – 1.31) | 1.32  (1.26 – 1.38) | <0.001 |
| **Integumentary** | 1.03  (0.95 – 1.12) | 1.08  (0.99 – 1.17) | 0.016 |
| **Intest/ panc** | 0.93  (0.90 – 0.96) | 1.01  (0.97 – 1.04) | 0.649 |
| **Liver** | 1.33  (1.26 – 1.40) | 1.20  (1.13 – 1.27) | <0.001 |
| **Lung** | 0.93  (0.90 – 0.97) | 1.19  (1.14 – 1.24) | <0.001 |
| **Reproductive** | 1.21  (1.06 – 1.39) | 1.08  (0.94 – 1.25) | 0.149 |
| **Skeletal** | 0.89  (0.86 – 0.92) | 1.01  (0.97 – 1.04) | 0.671 |
| **Systemic** | 1.06  (0.95 – 1.19) | 1.01  (0.91 – 1.14) | 0.736 |
| **Urinary** | 0.97  (0.88 – 1.06) | 1.06  (0.97 – 1.16) | 0.108 |
| **Valve** | 0.91  (0.86 – 0.96) | 1.07  (1.01 – 1.13) | 0.003 |

Table 37| Prevalence of fibrotic conditions per exposure group based upon prescription of insulin.

|  | **Whole cohort %**  **(95% CI)** | **No insulin prescription %**  **(95% CI)** | **Prescribed insulin % (95% CI)** |
| --- | --- | --- | --- |
| **Atherosclerosis** | 4.24  (4.18 – 4.29) | 3.64  (3.59 – 3.70) | 6.91  (6.74 – 7.07) |
| **Biliary** | 3.69  (3.64 – 3.75) | 3.40  (3.35 – 3.46) | 5.01  (4.87 – 5.15) |
| **Blood vessel** | 2.25  (2.21 – 2.29) | 2.08  (2.04 – 2.13) | 2.98  (2.89 – 3.11) |
| **Cardiomyopathy** | 18.13  (18.03 – 18.24) | 16.43  (16.31 – 16.54) | 25.83  (25.55 – 26.12) |
| **Integumentary** | 5.14  (5.08 – 5.20) | 4.76  (4.70 – 4.83) | 6.84  (6.68 – 7.00) |
| **Intest/ panc** | 26.36  (26.24 – 26.48) | 25.18  (25.05 – 25.31) | 21.91  (31.61 – 32.21) |
| **Liver** | 6.44  (6.38 – 6.51) | 5.67  (5.60 – 5.74) | 9.92  (9.73 – 10.11) |
| **Reproductive** | 2.40  (2.34 – 2.46) | 2.34  (2.27 – 2.41) | 2.64  (2.49 – 2.79) |
| **Lung** | 12.08  (11.99 – 12.17) | 10.98  (10.89 – 11.08) | 17.04  (16.79 – 17.28) |
| **Skeletal** | 26.82  (26.70 – 26.94) | 25.42  (25.29 – 25.55) | 33.14  (32.84 – 33.45) |
| **Systemic** | 1.94  (1.90 – 1.98) | 1.64  (1.60 – 1.68) | 3.32  (3.20 – 3.43) |
| **Urinary** | 4.09  (4.03 – 4.14) | 3.53  (3.47 – 3.58) | 6.61  (6.45 – 6.77) |
| **Valve** | 6.75  (6.68 – 6.82) | 6.26  (6.19 – 6.33) | 8.94  (8.75 – 9.12) |

Table 38| *Crude and fully adjusted odds ratios for the development of each fibrotic condition in the three years post index date. Fully adjusted analyses included adjustment for age, sex, smoking status, deprivation, number of fibrotic conditions at baseline and hypertension severity.*

|  | **Insulin prescription** | | |
| --- | --- | --- | --- |
|  | **Crude OR (99% CI)** | **Fully adjusted**  **OR (99% CI)** | **p-value** |
| **Atherosclerosis** | 2.05  (1.91 – 2.20) | 1.72  (1.60 – 1.85) | <0.001 |
| **Biliary** | 1.37  (1.26 – 1.50) | 1.08  (0.98 – 1.18) | 0.037 |
| **Blood vessel** | 1.42  (1.26 – 1.59) | 1.25  (1.11 – 1.41) | <0.001 |
| **Cardiomyopathy** | 1.60  (1.52 – 1.67) | 1.39  (1.33 – 1.47) | <0.001 |
| **Integumentary** | 1.48  (1.36 – 1.62) | 1.16  (1.06 – 1.27) | <0.001 |
| **Intest/ panc** | 1.28  (1.23 – 1.32) | 1.09  (1.05 – 1.13) | <0.001 |
| **Liver** | 1.59  (1.50 – 1.69) | 1.17  (1.10 – 1.25) | <0.001 |
| **Lung** | 1.53  (1.47 – 1.59) | 1.49  (1.43 – 1.56) | <0.001 |
| **Reproductive** | 1.58  (1.36 – 1.84) | 1.22  (1.04 – 1.42) | 0.001 |
| **Skeletal** | 1.31  (1.26 – 1.36) | 1.19  (1.14 – 1.23) | <0.001 |
| **Systemic** | 1.74  (1.55 – 1.95) | 1.09  (0.97 – 1.23) | 0.057 |
| **Urinary** | 1.50  (1.37 – 1.66) | 1.27  (1.15 – 1.40) | <0.001 |
| **Valve** | 1.42  (1.34 – 1.51) | 1.17  (1.10 – 1.25) | <0.001 |

Table 39| Prevalence of fibrotic conditions respective to prescription of metformin (or lack of).

|  | **Whole cohort %**  **(95% CI)** | **No metformin prescription %**  **(95% CI)** | **Prescribed metformin %**  **(95% CI)** |
| --- | --- | --- | --- |
| **Atherosclerosis** | 4.24  (4.18 – 4.29) | 4.48  (4.41 – 4.55) | 3.68  (3.58 – 3.77) |
| **Biliary** | 3.69  (3.64 – 3.75) | 3.96  (3.90 – 4.03) | 3.09  (3.01 – 3.18) |
| **Blood vessel** | 2.25  (2.21 – 2.29) | 2.37  (2.32 – 2.42) | 1.99  (1.92 – 2.05) |
| **Cardiomyopathy** | 18.13  (18.03 – 18.24) | 18.34  (18.22 – 18.47) | 17.59  (17.41 – 17.78) |
| **Integumentary** | 5.14  (5.08 – 5.20) | 5.46  (5.38 – 5.53) | 4.42  (4.32 – 4.52) |
| **Intest/ panc** | 26.36  (26.24 – 26.48) | 27.32  (27.17 – 27.47) | 24.14  (23.93 – 24.35) |
| **Liver** | 6.44  (6.38 – 6.51) | 6.51  (6.43 – 6.59) | 6.27  (6.15 – 6.39) |
| **Reproductive** | 2.40  (2.34 – 2.46) | 2.36  (2.28 – 2.44) | 2.49  (2.37 – 2.61) |
| **Lung** | 12.08  (11.99 – 12.17) | 12.77  (12.66 – 12.88) | 10.52  (10.37 – 10.67) |
| **Skeletal** | 26.82  (26.70 – 26.94) | 27.85  (27.70 – 27.99) | 24.46  (24.25 – 24.68) |
| **Systemic** | 1.94  (1.90 – 1.98) | 2.11  (2.06 – 215) | 1.57  (1.51 – 1.63) |
| **Urinary** | 4.09  (4.03 – 4.14) | 4.56  (4.49 – 4.63) | 3.04  (2.96 – 3.12) |
| **Valve** | 6.75  (6.68 – 6.82) | 7.19  (7.11 – 7.28) | 5.74  (5.62 – 5.85) |

Table 40 | *Crude and fully adjusted odds ratios for the development of each fibrotic condition in the three years post index date. Fully adjusted analyses included adjustment for age, sex, smoking status, deprivation, number of fibrotic conditions at baseline and hypertension severity.*

|  | **Metformin prescription** | | |
| --- | --- | --- | --- |
|  | **Crude OR (99% CI)** | **Fully adjusted**  **OR (99% CI)** | **p-value** |
| **Atherosclerosis** | 0.87  (0.81 – 0.94) | 1.02  (0.95 – 1.10) | 0.448 |
| **Biliary** | 0.78  (0.72 – 0.85) | 0.91  (0.83 – 0.99) | 0.003 |
| **Blood vessel** | 0.87  (0.78 – 0.98) | 1.00  (0.90 – 1.12) | 0.950 |
| **Cardiomyopathy** | 0.99  (0.94 – 1.03) | 1.06  (1.01 – 1.11) | 0.001 |
| **Integumentary** | 0.84  (0.77 – 0.91) | 0.96  (0.88 – 1.04) | 0.167 |
| **Intest/ panc** | 0.91  (0.88 – 0.94) | 1.02  (0.99 – 1.06) | 0.076 |
| **Liver** | 1.03  (0.97 – 1.09) | 1.05  (1.00 – 1.11) | 0.018 |
| **Lung** | 0.80  (0.77 – 0.83) | 1.00  (0.96 – 1.04) | 0.995 |
| **Reproductive** | 1.01  (0.88 – 1.16) | 1.00  (0.87 – 1.16) | 0.935 |
| **Skeletal** | 0.89  (0.86 – 0.92) | 1.00  (0.97 – 1.03) | 0.991 |
| **Systemic** | 0.83  (0.74 – 0.93) | 0.97  (0.86 – 1.09) | 0.462 |
| **Urinary** | 0.84  (0.77 – 0.92) | 0.95  (0.86 – 1.04) | 0.150 |
| **Valve** | 0.82  (0.77 – 0.87) | 1.02  (0.96 – 1.08) | 0.343 |

## **Type 2 diabetes sensitivity analyses**

Table 41 | Fully adjusted odds ratios for the development of each fibrotic condition in the three years post index date. Fully adjusted analyses included adjustment for age, sex, smoking status, deprivation, number of fibrotic conditions at baseline and hypertension severity. This sensitivity analysis included Charlson comorbidity index (CCI) as a covariate, where findings differ these are made apparent with red text.

| **Microvascular complications** | | |
| --- | --- | --- |
| **Fibrotic condition** | **Fully adjusted**  **OR (99% CI)** | **Fully adjusted**  **OR + CCI (99% CI)** |
| **Atherosclerosis** | 1.28  (1.15 – 1.42) | 1.28  (1.14 – 1.43) |
| **Biliary** | 0.95  (0.84 – 1.09) | 0.96  (0.84 – 1.11) |
| **Blood vessel** | 1.08  (0.90 – 1.29) | 1.08  (0.90 – 1.31) |
| **Cardiomyopathy** | 1.11  (1.02 – 1.20) | 1.14  (1.04 – 1.25) |
| **Integumentary** | 1.29  (1.13 – 1.46) | 1.32  (1.15 – 1.52) |
| **Intest/ panc** | 1.01  (0.95 – 1.07) | 1.01  (0.95 – 1.08) |
| **Liver** | 0.83  (0.74 – 0.93) | 0.87  (0.77 – 0.98) |
| **Lung** | 1.41  (1.33 – 1.49) | 1.37  (1.29 – 1.45) |
| **Reproductive** | 1.15  (0.88 – 1.50) | 1.15  (0.87 – 1.51) |
| **Skeletal** | 1.14  (1.07 – 1.20) | 1.11  (1.05 – 1.19) |
| **Systemic** | 1.53  (1.30 – 1.79) | 1.51  (1.27 – 1.79) |
| **Urinary** | 2.23  (1.98 – 2.52) | 2.22  (1.96 – 2.53) |
| **Valve** | 1.35  (1.24 – 1.46) | 1.38  (1.27 – 1.49) |

|  | **ACR elevated/ eGFR norm** | | **ACR elevated/ eGFR decreased** | | **ACR norm / eGFR decreased** | |
| --- | --- | --- | --- | --- | --- | --- |
| **Fibrotic condition** | **Fully adjusted**  **OR (99% CI)** | **Fully adjusted**  **OR + CCI (99% CI)** | **Fully adjusted**  **OR (99% CI)** | **Fully adjusted**  **OR + CCI (99% CI)** | **Fully adjusted**  **OR (99% CI)** | **Fully adjusted**  **OR + CCI (99% CI)** |
| **Atherosclerosis** | 1.56  (1.41 – 1.73) | 1.51  (1.35 – 1.69) | 1.63  (1.45 – 1.83) | 1.61  (1.43 – 1.82) | 1.10  (0.97 – 1.25) | 1.10  (0.96 – 1.26) |
| **Biliary** | 0.93  (0.82 –1.05) | 0.91  (0.80 – 1.05) | 0.95  (0.82 – 1.09) | 0.94  (0.81 – 1.09) | 1.08  (0.95 – 1.23) | 1.05  (0.91 – 1.21) |
| **Blood vessel** | 1.13  (0.95 – 1.34) | 1.14  (0.94 – 1.37) | 1.27  (1.05 – 1.54) | 1.31  (1.07 – 1.61) | 1.37  (1.15 – 1.63) | 1.43  (1.18 – 1.72) |
| **Cardiomyopathy** | 1.24  (1.16 – 1.32) | 1.20  (1.11 – 1.29) | 1.26  (1.15 – 1.37) | 1.25  (1.14 – 1.37) | 1.05  (0.97 – 1.15) | 1.03  (0.94 – 1.14) |
| **Integumentary** | 1.11  (0.98 – 1.26) | 1.15  (1.00 – 1.32) | 1.43  (1.25 – 1.65) | 1.48  (1.28 – 1.72) | 1.12  (0.98 – 1.30) | 1.16  (0.99 – 1.35) |
| **Intest/ panc** | 1.09  (1.04 – 1.15) | 1.08  (1.02 – 1.14) | 1.06  (0.99 – 1.13) | 1.05  (0.98 – 1.12) | 1.02  (0.96 – 1.08) | 1.00  (0.94 – 1.07) |
| **Liver** | 1.07  (0.99 – 1.17) | 1.08  (0.98 – 1.18) | 0.90  (0.80 – 1.02) | 0.92  (0.81 – 1.04) | 0.85  (0.76 – 0.96) | 0.86  (0.76 – 0.98) |
| **Lung** | 1.57  (1.48 – 1.66) | 1.51  (1.42 – 1.61) | 1.84  (1.73 – 1.96) | 1.76  (1.64 – 1.88) | 1.27  (1.19 – 1.35) | 1.24  (1.16 – 1.33) |
| **Reproductive** | 1.26  (1.02 – 1.54) | 1.17  (0.93 – 1.48) | 1.22  (0.93 – 1.62) | 1.20  (0.90 – 1.61) | 1.04  (0.77 – 1.38) | 1.06  (0.78 – 1.44) |
| **Skeletal** | 1.10  (1.05 – 1.16) | 1.09  (1.03 – 1.15) | 1.21  (1.14 – 1.28) | 1.20  (1.12 – 1.28) | 1.15  (1.09 – 1.22) | 1.14  (1.08 – 1.22) |
| **Systemic** | 1.49  (1.27 – 1.76) | 1.50  (1.25 – 1.80) | 2.02  (1.69 – 2.42) | 2.02  (1.67 – 2.45) | 1.45  (1.19 – 1.76) | 1.49  (1.21 – 1.84) |
| **Urinary** | 2.11  (1.86 – 2.41) | 2.08  (1.80 – 2.39) | 3.48  (3.03 – 4.00) | 3.30  (2.84 – 3.83) | 1.75  (1.49 – 2.05) | 1.69  (1.43 – 2.01) |
| **Valve** | 1.37  (1.26 – 1.48) | 1.36  (1.24 – 1.48) | 1.44  (1.32 – 1.57) | 1.48  (1.35 – 1.62) | 1.11  (1.02 – 1.22) | 1.12  (1.02 – 1.23) |

Table 42| Fully adjusted odds ratios for the development of each fibrotic condition in the three years post index date. Fully adjusted analyses included adjustment for age, sex, smoking status, deprivation, number of fibrotic conditions at baseline and hypertension severity. This sensitivity analysis included Charlson comorbidity index (CCI) as a covariate, where findings differ these are made apparent with red text.

Table 43 | Fully adjusted odds ratios for the development of each fibrotic condition in the three years post index date. Fully adjusted analyses included adjustment for age, sex, smoking status, deprivation, number of fibrotic conditions at baseline and hypertension severity. This sensitivity analysis included Charlson comorbidity index (CCI) as a covariate, where findings differ these are made apparent with red text.

|  | **Persistent ACR elevated/ eGFR norm** | | **Persistent ACR elevated/ Persistent eGFR decreased** | | **ACR norm / Persistent eGFR decreased** | |
| --- | --- | --- | --- | --- | --- | --- |
| **Fibrotic condition** | **Fully adjusted**  **OR (99% CI)** | **Fully adjusted**  **OR + CCI (99% CI)** | **Fully adjusted**  **OR (99% CI)** | **Fully adjusted**  **OR + CCI (99% CI)** | **Fully adjusted**  **OR (99% CI)** | **Fully adjusted**  **OR + CCI (99% CI)** |
|  |  |  |  |  |  |  |
| **Atherosclerosis** | 1.51  (1.34 – 1.70) | 1.49  (1.31 – 1.70) | 1.54  (1.36 – 1.74) | 1.52  (1.33 – 1.74) | 1.14  (1.00 – 1.29) | 1.14  (1.00 – 1.31) |
| **Biliary** | 1.02  (0.88 – 1.18) | 0.99  (0.85 – 1.17) | 1.02  (0.87 – 1.19) | 1.00  (0.85 – 1.18) | 1.12  (0.98 – 1.28) | 1.09  (0.94 – 1.26) |
| **Blood vessel** | 1.08  (0.88 – 1.32) | 1.11  (0.89 – 1.38) | 1.24  (1.01 – 1.53) | 1.25  (1.00 – 1.56) | 1.34  (1.11 – 1.60) | 1.37  (1.12 – 1.67) |
| **Cardiomyopathy** | 1.28  (1.19 – 1.38) | 1.24  (1.13 – 1.35) | 1.20  (1.09 – 1.31) | 1.22  (1.10 – 1.34) | 1.03  (0.94 – 1.12) | 1.01  (0.92 – 1.12) |
| **Integumentary** | 1.05  (0.91 – 1.22) | 1.12  (0.95 – 1.32) | 1.40  (1.21 – 1.62) | 1.48  (1.26 – 1.74) | 1.12  (0.96 – 1.30) | 1.18  (1.01 – 1.39) |
| **Intest/ panc** | 1.11  (1.05 – 1.18) | 1.11  (1.04 – 1.18) | 1.06  (0.99 – 1.13) | 1.05  (0.98 – 1.14) | 1.02  (0.96 – 1.09) | 1.02  (0.95 – 1.09) |
| **Liver** | 1.09  (0.98 – 1.20) | 1.11  (0.99 – 1.23) | 0.90  (0.79 – 1.03) | 0.92  (0.80 – 1.05) | 0.90  (0.80 – 1.02) | 0.89  (0.78 – 1.01) |
| **Lung** | 1.54  (1.44 – 1.65) | 1.49  (1.38 – 1.60) | 1.77  (1.65 – 1.89) | 1.68  (1.56 – 1.80) | 1.24  (1.16 – 1.32) | 1.20  (1.12 – 1.29) |
| **Reproductive** | 1.17  (0.91 – 1.49) | 1.15  (0.88 – 1.51) | 1.21  (0.90 – 1.63) | 1.20  (0.88 – 1.65) | 0.97  (0.72 – 1.31) | 0.98  (0.71 – 1.36) |
| **Skeletal** | 1.10  (1.04 – 1.16) | 1.09  (1.02 – 1.16) | 1.21  (1.13 – 1.29) | 1.17  (1.09 – 1.26) | 1.14  (1.08 – 1.21) | 1.13  (1.06 – 1.21) |
| **Systemic** | 1.50  (1.24 – 1.81) | 1.52  (1.24 – 1.87) | 1.91  (1.58 – 2.32) | 1.92  (1.56 – 2.36) | 1.37  (1.12 – 1.67) | 1.39  (1.12 – 1.73) |
| **Urinary** | 2.02  (1.74 – 2.35) | 1.95  (1.66 – 2.30) | 3.22  (2.77 – 3.75) | 3.09  (2.63 – 3.64) | 1.73  (1.47 – 2.04) | 1.65  (1.39 – 1.97) |
| **Valve** | 1.44  (1.31 – 1.58) | 1.44  (1.30 – 1.60) | 1.48  (1.35 – 1.63) | 1.50  (1.36 – 1.66) | 1.11  (1.01 – 1.21) | 1.09  (0.99 – 1.210 |

Table 44 | Fully adjusted odds ratios for the development of each fibrotic condition in the three years post index date. Fully adjusted analyses included adjustment for age, sex, smoking status, deprivation, number of fibrotic conditions at baseline and hypertension severity. This sensitivity analysis included Charlson comorbidity index (CCI) as a covariate, where findings differ these are made apparent with red text.

|  | **Glucose above target** | |
| --- | --- | --- |
|  | **Fully adjusted**  **OR (99% CI)** | **Fully adjusted**  **OR + CCI (99% CI)** |
| **Atherosclerosis** | 1.45  (1.36 – 1.56) | 1.45  (1.35 – 1.57) |
| **Biliary** | 1.01  (0.93 – 1.10) | 1.02  (0.93 – 1.11) |
| **Blood vessel** | 1.16  (1.04 – 1.30) | 1.15  (1.02 – 1.29) |
| **Cardiomyopathy** | 1.32  (1.26 – 1.38) | 1.31  (1.25 – 1.38) |
| **Integumentary** | 1.08  (0.99 – 1.17) | 1.10  (1.00 – 1.20) |
| **Intest/ panc** | 1.01  (0.97 – 1.04) | 1.02  (0.98 – 1.06) |
| **Liver** | 1.20  (1.13 – 1.27) | 1.19  (1.12 – 1.27) |
| **Lung** | 1.19  (1.14 – 1.24) | 1.18  (1.13 – 1.23) |
| **Reproductive** | 1.08  (0.94 – 1.25) | 1.09  (0.93 – 1.27) |
| **Skeletal** | 1.01  (0.97 – 1.04) | 1.01  (0.97 – 1.04) |
| **Systemic** | 1.01  (0.91 – 1.14) | 1.03  (0.91 – 1.16) |
| **Urinary** | 1.06  (0.97 – 1.16) | 1.05  (0.95 – 1.16) |
| **Valve** | 1.07  (1.01 – 1.13) | 1.08  (1.01 – 1.14) |

Table 45 | Fully adjusted odds ratios for the development of each fibrotic condition in the three years post index date. Fully adjusted analyses included adjustment for age, sex, smoking status, deprivation, number of fibrotic conditions at baseline and hypertension severity. This sensitivity analysis included Charlson comorbidity index (CCI) as a covariate, where findings differ these are made apparent with red text.

|  | **Insulin prescription** | |
| --- | --- | --- |
|  | **Fully adjusted**  **OR (99% CI)** | **Fully adjusted**  **OR + CCI (99% CI)** |
| **Atherosclerosis** | 1.72  (1.60 – 1.85) | 1.71  (1.58 – 1.86) |
| **Biliary** | 1.08  (0.98 – 1.18) | 1.08  (0.99 – 1.19) |
| **Blood vessel** | 1.25  (1.11 – 1.41) | 1.23  (1.08 – 1.40) |
| **Cardiomyopathy** | 1.39  (1.33 – 1.47) | 1.38  (1.30 – 1.45) |
| **Integumentary** | 1.16  (1.06 – 1.27) | 1.19  (1.08 – 1.31) |
| **Intest/ panc** | 1.09  (1.05 – 1.13) | 1.06  (1.02 – 1.11) |
| **Liver** | 1.17  (1.10 – 1.25) | 1.16  (1.08 – 1.24) |
| **Lung** | 1.49  (1.43 – 1.56) | 1.43  (1.37 – 1.50) |
| **Reproductive** | 1.22  (1.04 – 1.42) | 1.16  (0.98 – 1.37) |
| **Skeletal** | 1.19  (1.14 – 1.23) | 1.15  (1.10 – 1.20) |
| **Systemic** | 1.09  (0.97 – 1.23) | 1.09  (0.96 – 1.24) |
| **Urinary** | 1.27  (1.15 – 1.40) | 1.21  (1.09 – 1.34) |
| **Valve** | 1.17  (1.10 – 1.25) | 1.16  (1.09 – 1.24) |

Table 46 | Fully adjusted odds ratios for the development of each fibrotic condition in the three years post index date. Fully adjusted analyses included adjustment for age, sex, smoking status, deprivation, number of fibrotic conditions at baseline and hypertension severity. This sensitivity analysis included Charlson comorbidity index (CCI) as a covariate, where findings differ these are made apparent with red text.

|  | **Metformin prescription** | |
| --- | --- | --- |
|  | **Fully adjusted**  **OR (99% CI)** | **Fully adjusted**  **OR + CCI (99% CI)** |
| **Atherosclerosis** | 1.02  (0.95 – 1.10) | 1.00  (0.92 – 1.08) |
| **Biliary** | 0.91  (0.83 – 0.99) | 0.88  (0.80 – 0.96) |
| **Blood vessel** | 1.00  (0.90 – 1.12) | 0.97  (0.85 – 1.10) |
| **Cardiomyopathy** | 1.06  (1.01 – 1.11) | 1.07  (1.02 – 1.13) |
| **Integumentary** | 0.96  (0.88 – 1.04) | 0.93  (0.84 – 1.02) |
| **Intest/ panc** | 1.02  (0.99 – 1.06) | 1.01  (0.98 – 1.05) |
| **Liver** | 1.05  (1.00 – 1.11) | 1.04  (0.98 – 1.11) |
| **Lung** | 1.00  (0.96 – 1.04) | 1.01  (0.97 – 1.05) |
| **Reproductive** | 1.00  (0.87 – 1.16) | 1.01  (0.86 – 1.18) |
| **Skeletal** | 1.00  (0.97 – 1.03) | 0.99  (0.95 – 1.03) |
| **Systemic** | 0.97  (0.86 – 1.09) | 0.91  (0.80 – 1.04) |
| **Urinary** | 0.95  (0.86 – 1.04) | 0.94  (0.85 – 1.04) |
| **Valve** | 1.02  (0.96 – 1.08) | 1.00  0.94 – 1.07 |

Table 47 | Fully adjusted odds ratios for the development of each fibrotic condition in the three years post index date. Fully adjusted analyses included adjustment for age, sex, smoking status, deprivation, number of fibrotic conditions at baseline and hypertension severity. This sensitivity analysis included prescription of oral corticosteroids (OCS) as a covariate, where findings differ these are made apparent with red text.

| **Microvascular complications** | | |
| --- | --- | --- |
| **Fibrotic condition** | **Fully adjusted**  **OR (99% CI)** | **Fully adjusted**  **OR + OCS (99% CI)** |
| **Atherosclerosis** | 1.28  (1.15 – 1.42) | 1.28  (1.15 – 1.42) |
| **Biliary** | 0.95  (0.84 – 1.09) | 0.95  (0.84 – 1.09) |
| **Blood vessel** | 1.08  (0.90 – 1.29) | 1.08  (0.90 – 1.29) |
| **Cardiomyopathy** | 1.11  (1.02 – 1.20) | 1.11  (1.02 – 1.20) |
| **Integumentary** | 1.29  (1.13 – 1.46) | 1.29  (1.13 – 1.46) |
| **Intest/ panc** | 1.01  (0.95 – 1.07) | 1.01  (0.95 – 1.07) |
| **Liver** | 0.83  (0.74 – 0.93) | 0.83  (0.74 – 0.93) |
| **Lung** | 1.41  (1.33 – 1.49) | 1.41  (1.33 – 1.49) |
| **Reproductive** | 1.15  (0.88 – 1.50) | 1.15  (0.88 – 1.50) |
| **Skeletal** | 1.14  (1.07 – 1.20) | 1.13  (1.07 – 1.20) |
| **Systemic** | 1.53  (1.30 – 1.79) | 1.53  (1.30 – 1.79) |
| **Urinary** | 2.23  (1.98 – 2.52) | 2.23  (1.98 – 2.52) |
| **Valve** | 1.35  (1.24 – 1.46) | 1.35  (1.24 – 1.46) |

|  | **ACR elevated/ eGFR norm** | | **ACR elevated/ eGFR decreased** | | **ACR norm / eGFR decreased** | |
| --- | --- | --- | --- | --- | --- | --- |
| **Fibrotic condition** | **Fully adjusted**  **OR (99% CI)** | **Fully adjusted**  **OR + OCS (99% CI)** | **Fully adjusted**  **OR (99% CI)** | **Fully adjusted**  **OR + OCS (99% CI)** | **Fully adjusted**  **OR (99% CI)** | **Fully adjusted**  **OR + OCS (99% CI)** |
| **Atherosclerosis** | 1.56  (1.41 – 1.73) | 1.56  (1.41 – 1.73) | 1.63  (1.45 – 1.83) | 1.63  (1.45 – 1.83) | 1.10  (0.97 – 1.25) | 1.10  (0.97 – 1.25) |
| **Biliary** | 0.93  (0.82 –1.05) | 0.93  (0.82 – 1.05) | 0.95  (0.82 – 1.09) | 0.95  (0.82 – 1.09) | 1.08  (0.95 – 1.23) | 1.08  (0.95 – 1.23) |
| **Blood vessel** | 1.13  (0.95 – 1.34) | 1.13  (0.95 – 1.34) | 1.27  (1.05 – 1.54) | 1.27  (1.05 – 1.54) | 1.37  (1.15 – 1.63) | 1.37  (1.15 – 1.63) |
| **Cardiomyopathy** | 1.24  (1.16 – 1.32) | 1.24  (1.16 – 1.32) | 1.26  (1.15 – 1.37) | 1.26  (1.15 – 1.37) | 1.05  (0.97 – 1.15) | 1.05  (0.97 – 1.15) |
| **Integumentary** | 1.11  (0.98 – 1.26) | 1.11  (0.98 – 1.26) | 1.43  (1.25 – 1.65) | 1.44  (1.25 – 1.65) | 1.12  (0.98 – 1.30) | 1.12  (0.97 – 1.30) |
| **Intest/ panc** | 1.09  (1.04 – 1.15) | 1.09  (1.04 – 1.15) | 1.06  (0.99 – 1.13) | 1.06  (0.99 – 1.13) | 1.02  (0.96 – 1.08) | 1.02  (0.96 – 1.08) |
| **Liver** | 1.07  (0.99 – 1.17) | 1.07  (0.99 – 1.17) | 0.90  (0.80 – 1.02) | 0.90  (0.80 – 1.02 | 0.85  (0.76 – 0.96) | 0.85  (0.76 – 0.96) |
| **Lung** | 1.57  (1.48 – 1.66) | 1.57  (1.48 – 1.66) | 1.84  (1.73 – 1.96) | 1.84  (1.73 – 1.96) | 1.27  (1.19 – 1.35) | 1.27  (1.19 – 1.35) |
| **Reproductive** | 1.26  (1.02 – 1.54) | 1.26  (1.02 – 1.54) | 1.22  (0.93 – 1.62) | 1.22  (0.93 – 1.62) | 1.04  (0.77 – 1.38) | 1.04  (0.77 – 1.38) |
| **Skeletal** | 1.10  (1.05 – 1.16) | 1.10  (1.05 – 1.16) | 1.21  (1.14 – 1.28) | 1.21  (1.14 – 1.28) | 1.15  (1.09 – 1.22) | 1.15  (1.09 – 1.22) |
| **Systemic** | 1.49  (1.27 – 1.76) | 1.49  (1.27 – 1.76) | 2.02  (1.69 – 2.42) | 2.02  (1.69 – 2.42) | 1.45  (1.19 – 1.76) | 1.45  (1.19 – 1.76) |
| **Urinary** | 2.11  (1.86 – 2.41) | 2.11  (1.86 – 2.41) | 3.48  (3.03 – 4.00) | 3.48  (3.03 – 4.00) | 1.75  (1.49 – 2.05) | 1.75  (1.49 – 2.05) |
| **Valve** | 1.37  (1.26 – 1.48) | 1.37  (1.26 – 1.48) | 1.44  (1.32 – 1.57) | 1.44  (1.32 – 1.57) | 1.11  (1.02 – 1.22) | 1.12  (1.02 – 1.22) |

Table 48| Fully adjusted odds ratios for the development of each fibrotic condition in the three years post index date. Fully adjusted analyses included adjustment for age, sex, smoking status, deprivation, number of fibrotic conditions at baseline and hypertension severity. This sensitivity analysis included prescription of oral corticosteroids (OCS) as a covariate, where findings differ these are made apparent with red text.

|  | **Persistent ACR elevated/ eGFR norm** | | **Persistent ACR elevated/ Persistent eGFR decreased** | | **ACR norm / Persistent eGFR decreased** | |
| --- | --- | --- | --- | --- | --- | --- |
| **Fibrotic condition** | **Fully adjusted**  **OR (99% CI)** | **Fully adjusted**  **OR + OCS (99% CI)** | **Fully adjusted**  **OR (99% CI)** | **Fully adjusted**  **OR + OCS (99% CI)** | **Fully adjusted**  **OR (99% CI)** | **Fully adjusted**  **OR + OCS (99% CI)** |
| **Atherosclerosis** | 1.51  (1.34 – 1.70) | 1.51  (1.34 – 1.70) | 1.54  (1.36 – 1.74) | 1.54  (1.36 – 1.74) | 1.14  (1.00 – 1.29) | 1.14  (1.00 – 1.29) |
| **Biliary** | 1.02  (0.88 – 1.18) | 1.02  (0.88 – 1.19) | 1.02  (0.87 – 1.19) | 1.02  (0.87 – 1.19) | 1.12  (0.98 – 1.28) | 1.12  (0.98 – 1.28) |
| **Blood vessel** | 1.08  (0.88 – 1.32) | 1.08  (0.88 – 1.32) | 1.24  (1.01 – 1.53) | 1.24  (1.01 – 1.53) | 1.34  (1.11 – 1.60) | 1.34  (1.11 – 1.60) |
| **Cardiomyopathy** | 1.28  (1.19 – 1.38) | 1.28  (1.19 – 1.38) | 1.20  (1.09 – 1.31) | 1.20  (1.09 – 1.31) | 1.03  (0.94 – 1.12) | 1.03  (0.94 – 1.12) |
| **Integumentary** | 1.05  (0.91 – 1.22) | 1.05  (0.91 – 1.22) | 1.40  (1.21 – 1.62) | 1.40  (1.21 – 1.63) | 1.12  (0.96 – 1.30) | 1.12  (0.96 – 1.29) |
| **Intest/ panc** | 1.11  (1.05 – 1.18) | 1.11  (1.05 – 1.18) | 1.06  (0.99 – 1.13) | 1.06  (0.99 – 1.13) | 1.02  (0.96 – 1.09) | 1.02  (0.96 – 1.09) |
| **Liver** | 1.09  (0.98 – 1.20) | 1.09  (0.98 – 1.20) | 0.90  (0.79 – 1.03) | 0.90  (0.79 – 1.03) | 0.90  (0.80 – 1.02) | 0.90  (0.80 – 1.02) |
| **Lung** | 1.54  (1.44 – 1.65) | 1.54  (1.44 – 1.65) | 1.77  (1.65 – 1.89) | 1.77  (1.65 – 1.89) | 1.24  (1.16 – 1.32) | 1.24  (1.16 – 1.32) |
| **Reproductive** | 1.17  (0.91 – 1.49) | 1.17  (0.91 – 1.49) | 1.21  (0.90 – 1.63) | 1.21  (0.90 – 1.63) | 0.97  (0.72 – 1.31) | 0.97  (0.72 – 1.31) |
| **Skeletal** | 1.10  (1.04 – 1.16) | 1.10  (1.04 – 1.16) | 1.21  (1.13 – 1.29) | 1.21  (1.13 – 1.29) | 1.14  (1.08 – 1.21) | 1.14  (1.08 – 1.21) |
| **Systemic** | 1.50  (1.24 – 1.81) | 1.50  (1.24 – 1.81) | 1.91  (1.58 – 2.32) | 1.91  (1.58 – 2.32) | 1.37  (1.12 – 1.67) | 1.37  (1.12 – 1.67) |
| **Urinary** | 2.02  (1.74 – 2.35) | 2.02  (1.74 – 2.35) | 3.22  (2.77 – 3.75) | 3.22  (2.77 – 3.75) | 1.73  (1.47 – 2.04) | 1.73  (1.47 – 2.04) |
| **Valve** | 1.44  (1.31 – 1.58) | 1.44  (1.31 – 1.58) | 1.48  (1.35 – 1.63) | 1.48  (1.35 – 1.63) | 1.11  (1.01 – 1.21) | 1.11  (1.01 – 1.21) |

Table 49| Fully adjusted odds ratios for the development of each fibrotic condition in the three years post index date. Fully adjusted analyses included adjustment for age, sex, smoking status, deprivation, number of fibrotic conditions at baseline and hypertension severity. This sensitivity analysis included prescription of oral corticosteroids (OCS) as a covariate, where findings differ these are made apparent with red text.

Table 50| Fully adjusted odds ratios for the development of each fibrotic condition in the three years post index date. Fully adjusted analyses included adjustment for age, sex, smoking status, deprivation, number of fibrotic conditions at baseline and hypertension severity. This sensitivity analysis included prescription of oral corticosteroids (OCS) as a covariate, where findings differ these are made apparent with red text.

|  | **Glucose above target** | |
| --- | --- | --- |
|  | **Fully adjusted**  **OR (99% CI)** | **Fully adjusted**  **OR + OCS (99% CI)** |
| **Atherosclerosis** | 1.45  (1.36 – 1.56) | 1.45  (1.35 – 1.56) |
| **Biliary** | 1.01  (0.93 – 1.10) | 1.01  (0.93 – 1.11) |
| **Blood vessel** | 1.16  (1.04 – 1.30) | 1.16  (1.04 – 1.30) |
| **Cardiomyopathy** | 1.32  (1.26 – 1.38) | 1.32  (1.26 – 1.38) |
| **Integumentary** | 1.08  (0.99 – 1.17) | 1.08  (0.99 – 1.17) |
| **Intest/ panc** | 1.01  (0.97 – 1.04) | 1.01  (0.97 – 1.04) |
| **Liver** | 1.20  (1.13 – 1.27) | 1.20  (1.13 – 1.27) |
| **Lung** | 1.19  (1.14 – 1.24) | 1.19  (1.14 – 1.24) |
| **Reproductive** | 1.08  (0.94 – 1.25) | 1.08  (0.94 – 1.25) |
| **Skeletal** | 1.01  (0.97 – 1.04) | 1.01  (0.97 – 1.04) |
| **Systemic** | 1.01  (0.91 – 1.14) | 1.01  (0.91 – 1.14) |
| **Urinary** | 1.06  (0.97 – 1.16) | 1.06  (0.97 – 1.16) |
| **Valve** | 1.07  (1.01 – 1.13) | 1.07  (1.01 – 1.13) |

Table 51 | Fully adjusted odds ratios for the development of each fibrotic condition in the three years post index date. Fully adjusted analyses included adjustment for age, sex, smoking status, deprivation, number of fibrotic conditions at baseline and hypertension severity. This sensitivity analysis included prescription of oral corticosteroids (OCS) as a covariate, where findings differ these are made apparent with red text.

|  | **Insulin prescription** | |
| --- | --- | --- |
|  | **Fully adjusted**  **OR (99% CI)** | **Fully adjusted**  **OR + OCS (99% CI)** |
| **Atherosclerosis** | 1.72  (1.60 – 1.85) | 1.72  (1.60 – 1.85) |
| **Biliary** | 1.08  (0.98 – 1.18) | 1.08  (0.98 – 1.18) |
| **Blood vessel** | 1.25  (1.11 – 1.41) | 1.25  (1.11 – 1.41) |
| **Cardiomyopathy** | 1.39  (1.33 – 1.47) | 1.39  (1.33 – 1.47) |
| **Integumentary** | 1.16  (1.06 – 1.27) | 1.16  (1.06 – 1.27) |
| **Intest/ panc** | 1.09  (1.05 – 1.13) | 1.09  (1.05 – 1.13) |
| **Liver** | 1.17  (1.10 – 1.25) | 1.17  (1.10 – 1.25) |
| **Lung** | 1.49  (1.43 – 1.56) | 1.49  (1.43 – 1.56) |
| **Reproductive** | 1.22  (1.04 – 1.42) | 1.22  (1.04 – 1.42) |
| **Skeletal** | 1.19  (1.14 – 1.23) | 1.19  (1.14 – 1.23) |
| **Systemic** | 1.09  (0.97 – 1.23) | 1.09  (0.97 – 1.23) |
| **Urinary** | 1.27  (1.15 – 1.40) | 1.27  (1.15 – 1.40) |
| **Valve** | 1.17  (1.10 – 1.25) | 1.17  (1.10 – 1.25) |

Table 52| Fully adjusted odds ratios for the development of each fibrotic condition in the three years post index date. Fully adjusted analyses included adjustment for age, sex, smoking status, deprivation, number of fibrotic conditions at baseline and hypertension severity. This sensitivity analysis included prescription of oral corticosteroids (OCS) as a covariate, where findings differ these are made apparent with red text.

|  | **Metformin prescription** | |
| --- | --- | --- |
|  | **Fully adjusted**  **OR (99% CI)** | **Fully adjusted**  **OR + OCS (99% CI)** |
| **Atherosclerosis** | 1.02  (0.95 – 1.10) | 0.97  (0.90 – 1.04) |
| **Biliary** | 0.91  (0.83 – 0.99) | 0.86  (0.79 – 0.94) |
| **Blood vessel** | 1.00  (0.90 – 1.12) | 0.97  (0.86 – 1.08) |
| **Cardiomyopathy** | 1.06  (1.01 – 1.11) | 1.02  (0.98 – 1.07) |
| **Integumentary** | 0.96  (0.88 – 1.04) | 0.91  (0.83 – 0.99) |
| **Intest/ panc** | 1.02  (0.99 – 1.06) | 0.99  (0.96 – 1.02) |
| **Liver** | 1.05  (1.00 – 1.11) | 1.00  (0.94 – 1.06) |
| **Lung** | 1.00  (0.96 – 1.04) | 0.96  (0.93 – 1.00) |
| **Reproductive** | 1.00  (0.87 – 1.16) | 0.96  (0.83 – 1.11) |
| **Skeletal** | 1.00  (0.97 – 1.03) | 0.98  (0.95 – 1.01) |
| **Systemic** | 0.97  (0.86 – 1.09) | 0.88  (0.78 – 0.98) |
| **Urinary** | 0.95  (0.86 – 1.04) | 0.91  (0.83 – 1.00) |
| **Valve** | 1.02  (0.96 – 1.08) | 0.97  (0.91 – 1.02) |

Table 53| Fully adjusted odds ratios for the development of each fibrotic condition in the three years post index date. Fully adjusted analyses included adjustment for age, sex, smoking status, deprivation, number of fibrotic conditions at baseline and hypertension severity. This sensitivity analysis included diabetes duration as a covariate, where findings differ these are made apparent with red text.

|  | **Microvascular complications** | |
| --- | --- | --- |
|  | **Fully adjusted**  **OR (99% CI)** | **Fully adjusted**  **OR + diabetes duration (99% CI)** |
| **Atherosclerosis** | 1.28  (1.15 – 1.42) | 1.28  (1.15 – 1.42) |
| **Biliary** | 0.95  (0.84 – 1.09) | 0.95  (0.84 – 1.09) |
| **Blood vessel** | 1.08  (0.90 – 1.29) | 1.08  (0.90 – 1.29) |
| **Cardiomyopathy** | 1.11  (1.02 – 1.20) | 1.11  (1.02 – 1.20) |
| **Integumentary** | 1.29  (1.13 – 1.46) | 1.29  (1.13 – 1.46) |
| **Intest/ panc** | 1.01  (0.95 – 1.07) | 1.01  (0.95 – 1.07) |
| **Liver** | 0.83  (0.74 – 0.93) | 0.83  (0.74 – 0.93) |
| **Lung** | 1.41  (1.33 – 1.49) | 1.41  (1.33 – 1.49) |
| **Reproductive** | 1.15  (0.88 – 1.50) | 1.15  (0.88 – 1.50) |
| **Skeletal** | 1.14  (1.07 – 1.20) | 1.13  (1.07 – 1.20) |
| **Systemic** | 1.53  (1.30 – 1.79) | 1.53  (1.30 – 1.79) |
| **Urinary** | 2.23  (1.98 – 2.52) | 2.23  (1.98 – 2.52) |
| **Valve** | 1.35  (1.24 – 1.46) | 1.35  (1.24 – 1.46) |

Table 54 | Fully adjusted odds ratios for the development of each fibrotic condition in the three years post index date. Fully adjusted analyses included adjustment for age, sex, smoking status, deprivation, number of fibrotic conditions at baseline and hypertension severity. This sensitivity analysis included diabetes duration as a covariate, where findings differ these are made apparent with red text.

|  | **ACR elevated/ SCR norm** | | **ACR elevated/ SCR elevated** | | **ACR norm / SCR elevated** | |
| --- | --- | --- | --- | --- | --- | --- |
| **Fibrotic condition** | **Fully adjusted**  **OR (99% CI)** | **Fully adjusted**  **OR + diabetes duration (99% CI)** | **Fully adjusted**  **OR (99% CI)** | **Fully adjusted**  **OR + diabetes duration (99% CI)** | **Fully adjusted**  **OR (99% CI)** | **Fully adjusted**  **OR + diabetes duration (99% CI)** |
| **Atherosclerosis** | 1.56  (1.41 – 1.73) | 1.56  (1.41 – 1.73) | 1.63  (1.45 – 1.83) | 1.63  (1.45 – 1.83) | 1.10  (0.97 – 1.25) | 1.10  (0.97 – 1.25) |
| **Biliary** | 0.93  (0.82 –1.05) | 0.93  (0.82 – 1.05) | 0.95  (0.82 – 1.09) | 0.95  (0.82 – 1.09) | 1.08  (0.95 – 1.23) | 1.08  (0.95 – 1.23) |
| **Blood vessel** | 1.13  (0.95 – 1.34) | 1.13  (0.95 – 1.34) | 1.27  (1.05 – 1.54) | 1.27  (1.05 – 1.54) | 1.37  (1.15 – 1.63) | 1.37  (1.15 – 1.63) |
| **Cardiomyopathy** | 1.24  (1.16 – 1.32) | 1.24  (1.16 – 1.33) | 1.26  (1.15 – 1.37) | 1.26  (1.16 – 1.37) | 1.05  (0.97 – 1.15) | 1.05  (0.97 – 1.15) |
| **Integumentary** | 1.11  (0.98 – 1.26) | 1.11  (0.98 – 1.26) | 1.43  (1.25 – 1.65) | 1.43  (1.25 – 1.65) | 1.12  (0.98 – 1.30) | 1.12  (0.98 – 1.30) |
| **Intest/ panc** | 1.09  (1.04 – 1.15) | 1.09  (1.04 – 1.15) | 1.06  (0.99 – 1.13) | 1.06  (0.99 – 1.13) | 1.02  (0.96 – 1.08) | 1.02  (0.96 – 1.08) |
| **Liver** | 1.07  (0.99 – 1.17) | 1.07  (0.99 – 1.17) | 0.90  (0.80 – 1.02) | 0.91  (0.80 – 1.02) | 0.85  (0.76 – 0.96) | 0.85  (0.76 – 0.96) |
| **Lung** | 1.57  (1.48 – 1.66) | 1.57  (1.48 – 1.67) | 1.84  (1.73 – 1.96) | 1.84  (1.73 – 1.96) | 1.27  (1.19 – 1.35) | 1.27  (1.19 – 1.35) |
| **Reproductive** | 1.26  (1.02 – 1.54) | 1.26  (1.02 – 1.54) | 1.22  (0.93 – 1.62) | 1.22  (0.93 – 1.62) | 1.04  (0.77 – 1.38) | 1.04  (0.77 – 1.38) |
| **Skeletal** | 1.10  (1.05 – 1.16) | 1.10  (1.05 – 1.15) | 1.21  (1.14 – 1.28) | 1.21  (1.14 – 1.28) | 1.15  (1.09 – 1.22) | 1.15  (1.09 – 1.22) |
| **Systemic** | 1.49  (1.27 – 1.76) | 1.49  (1.27 – 1.76) | 2.02  (1.69 – 2.42) | 2.02  (1.69 – 2.41) | 1.45  (1.19 – 1.76) | 1.45  (1.19 – 1.76) |
| **Urinary** | 2.11  (1.86 – 2.41) | 2.11  (1.86 – 2.41) | 3.48  (3.03 – 4.00) | 3.48  (3.03 – 4.00) | 1.75  (1.49 – 2.05) | 1.75  (1.49 – 2.05) |
| **Valve** | 1.37  (1.26 – 1.48) | 1.37  (1.26 – 1.48) | 1.44  (1.32 – 1.57) | 1.44  (1.32 – 1.57) | 1.11  (1.02 – 1.22) | 1.11  (1.02 – 1.22) |

|  | **Persistent ACR elevated/ eGFR norm** | | **Persistent ACR elevated/ Persistent eGFR decreased** | | **ACR norm / Persistent eGFR decreased** | |
| --- | --- | --- | --- | --- | --- | --- |
| **Fibrotic condition** | **Fully adjusted**  **OR (99% CI)** | **Fully adjusted**  **OR + diabetes duration (99% CI)** | **Fully adjusted**  **OR (99% CI)** | **Fully adjusted**  **OR + diabetes duration (99% CI)** | **Fully adjusted**  **OR (99% CI)** | **Fully adjusted**  **OR + diabetes duration (99% CI)** |
| **Atherosclerosis** | 1.51  (1.34 – 1.70) | 1.51  (1.34 – 1.70) | 1.54  (1.36 – 1.74) | 1.54  (1.36 – 1.74) | 1.14  (1.00 – 1.29) | 1.14  (1.00 – 1.29) |
| **Biliary** | 1.02  (0.88 – 1.18) | 1.02  (0.88 – 1.18) | 1.02  (0.87 – 1.19) | 1.02  (0.87 – 1.19) | 1.12  (0.98 – 1.28) | 1.12  (0.98 – 1.28) |
| **Blood vessel** | 1.08  (0.88 – 1.32) | 1.08  (0.88 – 1.32) | 1.24  (1.01 – 1.53) | 1.24  (1.01 – 1.53) | 1.34  (1.11 – 1.60) | 1.34  (1.11 – 1.61) |
| **Cardiomyopathy** | 1.28  (1.19 – 1.38) | 1.28  (1.19 – 1.38) | 1.20  (1.09 – 1.31) | 1.20  (1.09 – 1.31) | 1.03  (0.94 – 1.12) | 1.03  (0.94 – 1.12) |
| **Integumentary** | 1.05  (0.91 – 1.22) | 1.05  (0.91 – 1.22) | 1.40  (1.21 – 1.62) | 1.40  (1.21 – 1.63) | 1.12  (0.96 – 1.30) | 1.12  (0.97 – 1.30) |
| **Intest/ panc** | 1.11  (1.05 – 1.18) | 1.11  (1.05 – 1.18) | 1.06  (0.99 – 1.13) | 1.06  (0.99 – 1.13) | 1.02  (0.96 – 1.09) | 1.02  (0.96 – 1.09) |
| **Liver** | 1.09  (0.98 – 1.20) | 1.09  (0.98 – 1.20) | 0.90  (0.79 – 1.03) | 0.90  (0.79 – 1.03) | 0.90  (0.80 – 1.02) | 0.90  (0.80 – 1.02) |
| **Lung** | 1.54  (1.44 – 1.65) | 1.54  (1.44 – 1.65) | 1.77  (1.65 – 1.89) | 1.77  (1.65 – 1.89) | 1.24  (1.16 – 1.32) | 1.24  (1.16 – 1.32) |
| **Reproductive** | 1.17  (0.91 – 1.49) | 1.17  (0.91 – 1.49) | 1.21  (0.90 – 1.63) | 1.21  (0.90 – 1.63) | 0.97  (0.72 – 1.31) | 0.97  (0.72 – 1.31) |
| **Skeletal** | 1.10  (1.04 – 1.16) | 1.10  (1.04 – 1.16) | 1.21  (1.13 – 1.29) | 1.21  (1.13 – 1.29) | 1.14  (1.08 – 1.21) | 1.14  (1.08 – 1.21) |
| **Systemic** | 1.50  (1.24 – 1.81) | 1.50  (1.24 – 1.81) | 1.91  (1.58 – 2.32) | 1.91  (1.58 – 2.32) | 1.37  (1.12 – 1.67) | 1.37  (1.12 – 1.67) |
| **Urinary** | 2.02  (1.74 – 2.35) | 2.02  (1.74 – 2.35) | 3.22  (2.77 – 3.75) | 3.22  (2.77 – 3.35) | 1.73  (1.47 – 2.04) | 1.73  (1.47 – 2.04) |
| **Valve** | 1.44  (1.31 – 1.58) | 1.44  (1.31 – 1.58) | 1.48  (1.35 – 1.63) | 1.48  (1.35 – 1.63) | 1.11  (1.01 – 1.21) | 1.10  (1.01 – 1.21) |

Table 55| Fully adjusted odds ratios for the development of each fibrotic condition in the three years post index date. Fully adjusted analyses included adjustment for age, sex, smoking status, deprivation, number of fibrotic conditions at baseline and hypertension severity. This sensitivity analysis included diabetes duration as a covariate, where findings differ these are made apparent with red text.

Table 56| Fully adjusted odds ratios for the development of each fibrotic condition in the three years post index date. Fully adjusted analyses included adjustment for age, sex, smoking status, deprivation, number of fibrotic conditions at baseline and hypertension severity. This sensitivity analysis included diabetes duration as a covariate, where findings differ these are made apparent with red text.

|  | **Glucose above target** | |
| --- | --- | --- |
|  | **Fully adjusted**  **OR (99% CI)** | **Fully adjusted**  **OR + diabetes duration (99% CI)** |
| **Atherosclerosis** | 1.45  (1.36 – 1.56) | 1.45  (1.36 – 1.56) |
| **Biliary** | 1.01  (0.93 – 1.10) | 1.01  (0.93 – 1.10) |
| **Blood vessel** | 1.16  (1.04 – 1.30) | 1.16  (1.04 – 1.30) |
| **Cardiomyopathy** | 1.32  (1.26 – 1.38) | 1.32  (1.26 – 1.38) |
| **Integumentary** | 1.08  (0.99 – 1.17) | 1.08  (0.99 – 1.17) |
| **Intest/ panc** | 1.01  (0.97 – 1.04) | 1.01  (0.97 – 1.04) |
| **Liver** | 1.20  (1.13 – 1.27) | 1.20  (1.13 – 1.27) |
| **Lung** | 1.19  (1.14 – 1.24) | 1.19  (1.15 – 1.24) |
| **Reproductive** | 1.08  (0.94 – 1.25) | 1.08  (0.94 – 1.25) |
| **Skeletal** | 1.01  (0.97 – 1.04) | 1.01  (0.97 – 1.04) |
| **Systemic** | 1.01  (0.91 – 1.14) | 1.01  (0.90 – 1.14) |
| **Urinary** | 1.06  (0.97 – 1.16) | 1.06  (0.97 – 1.16) |
| **Valve** | 1.07  (1.01 – 1.13) | 1.07  (1.01 – 1.13) |

Table 57| Fully adjusted odds ratios for the development of each fibrotic condition in the three years post index date. Fully adjusted analyses included adjustment for age, sex, smoking status, deprivation, number of fibrotic conditions at baseline and hypertension severity. This sensitivity analysis included diabetes duration as a covariate, where findings differ these are made apparent with red text.

|  | **Insulin prescription** | |
| --- | --- | --- |
|  | **Fully adjusted**  **OR (99% CI)** | **Fully adjusted**  **OR + diabetes duration (99% CI)** |
| **Atherosclerosis** | 1.72  (1.60 – 1.85) | 1.72  (1.60 – 1.85) |
| **Biliary** | 1.08  (0.98 – 1.18) | 1.07  (0.98 – 1.18) |
| **Blood vessel** | 1.25  (1.11 – 1.41) | 1.25  (1.11 – 1.41) |
| **Cardiomyopathy** | 1.39  (1.33 – 1.47) | 1.39  (1.33 – 1.47) |
| **Integumentary** | 1.16  (1.06 – 1.27) | 1.16  (1.06 – 1.27) |
| **Intest/ panc** | 1.09  (1.05 – 1.13) | 1.09  (1.05 – 1.13) |
| **Liver** | 1.17  (1.10 – 1.25) | 1.17  (1.10 – 1.25) |
| **Lung** | 1.49  (1.43 – 1.56) | 1.50  (1.43 – 1.56) |
| **Reproductive** | 1.22  (1.04 – 1.42) | 1.22  (1.04 – 1.42) |
| **Skeletal** | 1.19  (1.14 – 1.23) | 1.19  (1.14 – 1.23) |
| **Systemic** | 1.09  (0.97 – 1.23) | 1.09  (0.97 – 1.23) |
| **Urinary** | 1.27  (1.15 – 1.40) | 1.27  (1.15 – 1.40) |
| **Valve** | 1.17  (1.10 – 1.25) | 1.17  (1.10 – 1.25) |

Table 58| | Fully adjusted odds ratios for the development of each fibrotic condition in the three years post index date. Fully adjusted analyses included adjustment for age, sex, smoking status, deprivation, number of fibrotic conditions at baseline and hypertension severity. This sensitivity analysis included diabetes duration as a covariate, where findings differ these are made apparent with red text.

|  | **Metformin prescription** | |
| --- | --- | --- |
|  | **Fully adjusted**  **OR (99% CI)** | **Fully adjusted**  **OR + diabetes duration (99% CI)** |
| **Atherosclerosis** | 1.02  (0.95 – 1.10) | 1.03  (0.95 – 1.11) |
| **Biliary** | 0.91  (0.83 – 0.99) | 0.91  (0.84 – 0.99) |
| **Blood vessel** | 1.00  (0.90 – 1.12) | 1.00  (0.90 – 1.12) |
| **Cardiomyopathy** | 1.06  (1.01 – 1.11) | 1.06  (1.01 – 1.11) |
| **Integumentary** | 0.96  (0.88 – 1.04) | 0.96  (0.88 – 1.04) |
| **Intest/ panc** | 1.02  (0.99 – 1.06) | 1.02  (0.99 – 1.06) |
| **Liver** | 1.05  (1.00 – 1.11) | 1.06  (1.00 – 1.12) |
| **Lung** | 1.00  (0.96 – 1.04) | 1.00  (0.96 – 1.04) |
| **Reproductive** | 1.00  (0.87 – 1.16) | 1.00  (0.87 – 1.16) |
| **Skeletal** | 1.00  (0.97 – 1.03) | 1.00  (0.97 – 1.03) |
| **Systemic** | 0.97  (0.86 – 1.09) | 0.97  (0.86 – 1.09) |
| **Urinary** | 0.95  (0.86 – 1.04) | 0.95  (0.86 – 1.04) |
| **Valve** | 1.02  (0.96 – 1.08) | 1.02  (0.97 – 1.08) |

Table 59| Fully adjusted odds ratios for the development of each fibrotic condition in the three years post index date. Fully adjusted analyses included adjustment for age, sex, smoking status, deprivation, number of fibrotic conditions at baseline and hypertension severity. This sensitivity analysis included body mass index (BMI) as a covariate, where findings differ these are made apparent with red text.

| **Microvascular complications** | | |
| --- | --- | --- |
| **Fibrotic condition** | **Fully adjusted**  **OR (99% CI)** | **Fully adjusted**  **OR + BMI (99% CI)** |
| **Atherosclerosis** | 1.28  (1.15 – 1.42) | 1.28  (1.15 – 1.43) |
| **Biliary** | 0.95  (0.84 – 1.09) | 0.93  (0.81 – 1.08) |
| **Blood vessel** | 1.08  (0.90 – 1.29) | 1.06  (0.87 – 1.28) |
| **Cardiomyopathy** | 1.11  (1.02 – 1.20) | 1.11  (1.02 – 1.21) |
| **Integumentary** | 1.29  (1.13 – 1.46) | 1.30  (1.14 – 1.49) |
| **Intest/ panc** | 1.01  (0.95 – 1.07) | 1.01  (0.94 – 1.07) |
| **Liver** | 0.83  (0.74 – 0.93) | 0.87  (0.76 – 0.98) |
| **Lung** | 1.41  (1.33 – 1.49) | 1.42  (1.34 – 1.51) |
| **Reproductive** | 1.15  (0.88 – 1.50) | 1.16  (0.87 – 1.54) |
| **Skeletal** | 1.14  (1.07 – 1.20) | 1.14  (1.07 – 1.21) |
| **Systemic** | 1.53  (1.30 – 1.79) | 1.55  (1.31 – 1.83) |
| **Urinary** | 2.23  (1.98 – 2.52) | 2.22  (1.96 – 2.52) |
| **Valve** | 1.35  (1.24 – 1.46) | 1.35  (1.24 – 1.46) |

Table 60| Fully adjusted odds ratios for the development of each fibrotic condition in the three years post index date. Fully adjusted analyses included adjustment for age, sex, smoking status, deprivation, number of fibrotic conditions at baseline and hypertension severity. This sensitivity analysis included body mass index (BMI) as a covariate, where findings differ these are made apparent with red text.

|  | **ACR elevated/ eGFR norm** | | **ACR elevated/ eGFR decreased** | | **ACR norm / eGFR decreased** | |
| --- | --- | --- | --- | --- | --- | --- |
| **Fibrotic condition** | **Fully adjusted**  **OR (99% CI)** | **Fully adjusted**  **OR + BMI (99% CI)** | **Fully adjusted**  **OR (99% CI)** | **Fully adjusted**  **OR + BMI (99% CI)** | **Fully adjusted**  **OR (99% CI)** | **Fully adjusted**  **OR + BMI (99% CI)** |
| **Atherosclerosis** | 1.56  (1.41 – 1.73) | 1.57  (1.42 – 1.75) | 1.63  (1.45 – 1.83) | 1.61  (1.43 – 1.82) | 1.10  (0.97 – 1.25) | 1.10  (0.97 – 1.25) |
| **Biliary** | 0.93  (0.82 –1.05) | 0.93  (0.81 – 1.06) | 0.95  (0.82 – 1.09) | 0.96  (0.82 – 1.11) | 1.08  (0.95 – 1.23) | 1.09  (0.95 – 1.24) |
| **Blood vessel** | 1.13  (0.95 – 1.34) | 1.15  (0.95 – 1.55) | 1.27  (1.05 – 1.54) | 1.26  (1.03 – 1.55) | 1.37  (1.15 – 1.63) | 1.39  (1.16 – 1.68) |
| **Cardiomyopathy** | 1.24  (1.16 – 1.32) | 1.22  (1.14 – 1.31) | 1.26  (1.15 – 1.37) | 1.26  (1.15 – 1.37) | 1.05  (0.97 – 1.15) | 1.06  (0.97 – 1.16) |
| **Integumentary** | 1.11  (0.98 – 1.26) | 1.07  (0.93 – 1.22) | 1.43  (1.25 – 1.65) | 1.41  (1.22 – 1.62) | 1.12  (0.98 – 1.30) | 1.08  (0.93 – 1.25) |
| **Intest/ panc** | 1.09  (1.04 – 1.15) | 1.09  (1.03 – 1.14) | 1.06  (0.99 – 1.13) | 1.04  (0.98 – 1.12) | 1.02  (0.96 – 1.08) | 1.00  (0.94 – 1.07) |
| **Liver** | 1.07  (0.99 – 1.17) | 1.08  (0.99 – 1.18) | 0.90  (0.80 – 1.02) | 0.93  (0.82 – 1.06) | 0.85  (0.76 – 0.96) | 0.85  (0.75 – 0.97) |
| **Lung** | 1.57  (1.48 – 1.66) | 1.56  (1.46 – 1.65) | 1.84  (1.73 – 1.96) | 1.84  (1.72 – 1.96) | 1.27  (1.19 – 1.35) | 1.25  (1.17 – 1.33) |
| **Reproductive** | 1.26  (1.02 – 1.54) | 1.29  (1.03 – 1.61) | 1.22  (0.93 – 1.62) | 1.28  (0.95 – 1.73) | 1.04  (0.77 – 1.38) | 1.08  (0.79 – 1.47) |
| **Skeletal** | 1.10  (1.05 – 1.16) | 1.09  (1.03 – 1.15) | 1.21  (1.14 – 1.28) | 1.21  (1.13 – 1.29) | 1.15  (1.09 – 1.22) | 1.13  (1.07 – 1.20) |
| **Systemic** | 1.49  (1.27 – 1.76) | 1.48  (1.24 – 1.77) | 2.02  (1.69 – 2.42) | 1.98  (1.64 – 2.39) | 1.45  (1.19 – 1.76) | 1.38  (1.12 – 1.69) |
| **Urinary** | 2.11  (1.86 – 2.41) | 2.13  (1.85 – 2.44) | 3.48  (3.03 – 4.00) | 3.50  (3.02 – 4.05) | 1.75  (1.49 – 2.05) | 1.77  (1.50 – 2.08) |
| **Valve** | 1.37  (1.26 – 1.48) | 1.37  (1.25 – 1.49) | 1.44  (1.32 – 1.57) | 1.43  (1.30 – 1.56) | 1.11  (1.02 – 1.22) | 1.09  (0.99 – 1.19) |

Table 61| Fully adjusted odds ratios for the development of each fibrotic condition in the three years post index date. Fully adjusted analyses included adjustment for age, sex, smoking status, deprivation, number of fibrotic conditions at baseline and hypertension severity. This sensitivity analysis included body mass index (BMI) as a covariate, where findings differ these are made apparent with red text.

|  | **Persistent ACR elevated/ eGFR norm** | | **Persistent ACR elevated/ Persistent eGFR decreased** | | **ACR norm / Persistent eGFR decreased** | |
| --- | --- | --- | --- | --- | --- | --- |
| **Fibrotic condition** | **Fully adjusted**  **OR (99% CI)** | **Fully adjusted**  **OR + BMI (99% CI)** | **Fully adjusted**  **OR (99% CI)** | **Fully adjusted**  **OR + BMI (99% CI)** | **Fully adjusted**  **OR (99% CI)** | **Fully adjusted**  **OR + BMI (99% CI)** |
| **Atherosclerosis** | 1.51  (1.34 – 1.70) | 1.52  (1.35 – 1.72) | 1.54  (1.36 – 1.74) | 1.54  (1.35 – 1.75) | 1.14  (1.00 – 1.29) | 1.14  (1.00 – 1.30) |
| **Biliary** | 1.02  (0.88 – 1.18) | 1.04  (0.89 – 1.19) | 1.02  (0.87 – 1.19) | 1.01  (0.86 – 1.19) | 1.12  (0.98 – 1.28) | 1.15  (0.99 – 1.32) |
| **Blood vessel** | 1.08  (0.88 – 1.32) | 1.09  (0.88 – 1.36) | 1.24  (1.01 – 1.53) | 1.23  (0.98 – 1.53) | 1.34  (1.11 – 1.60) | 1.34  (1.10 – 1.63) |
| **Cardiomyopathy** | 1.28  (1.19 – 1.38) | 1.29  (1.19 – 1.40) | 1.20  (1.09 – 1.31) | 1.21  (1.10 – 1.33) | 1.03  (0.94 – 1.12) | 1.03  (0.94 – 1.13) |
| **Integumentary** | 1.05  (0.91 – 1.22) | 1.03  (0.88 – 1.21) | 1.40  (1.21 – 1.62) | 1.40  (1.20 – 1.64) | 1.12  (0.96 – 1.30) | 1.09  (0.93 – 1.27) |
| **Intest/ panc** | 1.11  (1.05 – 1.18) | 1.12  (1.05 – 1.19) | 1.06  (0.99 – 1.13) | 1.05  (0.98 – 1.13) | 1.02  (0.96 – 1.09) | 1.02  (0.95 – 1.19) |
| **Liver** | 1.09  (0.98 – 1.20) | 1.06  (0.96 – 1.18) | 0.90  (0.79 – 1.03) | 0.94  (0.82 – 1.07) | 0.90  (0.80 – 1.02) | 0.88  (0.77 – 1.00) |
| **Lung** | 1.54  (1.44 – 1.65) | 1.53  (1.42 – 1.64) | 1.77  (1.65 – 1.89) | 1.77  (1.42 – 1.64) | 1.24  (1.16 – 1.32) | 1.21  (1.13 – 1.30) |
| **Reproductive** | 1.17  (0.91 – 1.49) | 1.21  (0.93 – 1.58) | 1.21  (0.90 – 1.63) | 1.25  (0.93 – 1.58) | 0.97  (0.72 – 1.31) | 1.04  (0.76 – 1.43) |
| **Skeletal** | 1.10  (1.04 – 1.16) | 1.09  (1.03 – 1.16) | 1.21  (1.13 – 1.29) | 1.20  (1.12 – 1.29) | 1.14  (1.08 – 1.21) | 1.13  (1.06 – 1.19) |
| **Systemic** | 1.50  (1.24 – 1.81) | 1.48  (1.21 – 1.81) | 1.91  (1.58 – 2.32) | 1.91  (1.56 – 2.34) | 1.37  (1.12 – 1.67) | 1.33  (1.07 – 1.64) |
| **Urinary** | 2.02  (1.74 – 2.35) | 2.04  (1.74 – 2.38) | 3.22  (2.77 – 3.75) | 3.17  (2.71 – 3.72) | 1.73  (1.47 – 2.04) | 1.73  (1.46 – 2.05) |
| **Valve** | 1.44  (1.31 – 1.58) | 1.43  (1.30 – 1.58) | 1.48  (1.35 – 1.63) | 1.47  (1.34 – 1.63) | 1.11  (1.01 – 1.21) | 1.09  (0.99 – 1.20) |

Table 62| Fully adjusted odds ratios for the development of each fibrotic condition in the three years post index date. Fully adjusted analyses included adjustment for age, sex, smoking status, deprivation, number of fibrotic conditions at baseline and hypertension severity. This sensitivity analysis included body mass index (BMI) as a covariate, where findings differ these are made apparent with red text.

|  | **Glucose above target** | |
| --- | --- | --- |
|  | **Fully adjusted**  **OR (99% CI)** | **Fully adjusted**  **OR + BMI (99% CI)** |
| **Atherosclerosis** | 1.45  (1.36 – 1.56) | 1.45  (1.34 – 1.56) |
| **Biliary** | 1.01  (0.93 – 1.10) | 1.00  (0.92 – 1.10) |
| **Blood vessel** | 1.16  (1.04 – 1.30) | 1.15  (1.02 – 1.29) |
| **Cardiomyopathy** | 1.32  (1.26 – 1.38) | 1.30  (1.24 – 1.37) |
| **Integumentary** | 1.08  (0.99 – 1.17) | 1.09  (1.00 – 1.19) |
| **Intest/ panc** | 1.01  (0.97 – 1.04) | 1.01  (0.97 – 1.04) |
| **Liver** | 1.20  (1.13 – 1.27) | 1.18  (1.11 – 1.26) |
| **Lung** | 1.19  (1.14 – 1.24) | 1.20  (1.15 – 1.25) |
| **Reproductive** | 1.08  (0.94 – 1.25) | 1.10  (0.94 – 1.28) |
| **Skeletal** | 1.01  (0.97 – 1.04) | 1.01  (0.98 – 1.05) |
| **Systemic** | 1.01  (0.91 – 1.14) | 1.03  (0.91 – 1.16) |
| **Urinary** | 1.06  (0.97 – 1.16) | 1.06  (0.96 – 1.17) |
| **Valve** | 1.07  (1.01 – 1.13) | 1.07  (1.01 – 1.14) |

Table 63| Fully adjusted odds ratios for the development of each fibrotic condition in the three years post index date. Fully adjusted analyses included adjustment for age, sex, smoking status, deprivation, number of fibrotic conditions at baseline and hypertension severity. This sensitivity analysis included body mass index (BMI) as a covariate, where findings differ these are made apparent with red text.

|  | **Insulin prescription** | |
| --- | --- | --- |
|  | **Fully adjusted**  **OR (99% CI)** | **Fully adjusted**  **OR + BMI (99% CI)** |
| **Atherosclerosis** | 1.72  (1.60 – 1.85) | 1.68  (1.55 – 1.82) |
| **Biliary** | 1.08  (0.98 – 1.18) | 1.07  (0.97 – 1.18) |
| **Blood vessel** | 1.25  (1.11 – 1.41) | 1.26  (1.10 – 1.43) |
| **Cardiomyopathy** | 1.39  (1.33 – 1.47) | 1.38  (1.31 – 1.46) |
| **Integumentary** | 1.16  (1.06 – 1.27) | 1.15  (1.04 – 1.27) |
| **Intest/ panc** | 1.09  (1.05 – 1.13) | 1.09  (1.04 – 1.13) |
| **Liver** | 1.17  (1.10 – 1.25) | 1.19  (1.11 – 1.28) |
| **Lung** | 1.49  (1.43 – 1.56) | 1.50  (1.44 – 1.57) |
| **Reproductive** | 1.22  (1.04 – 1.42) | 1.26  (1.06 – 1.49) |
| **Skeletal** | 1.19  (1.14 – 1.23) | 1.20  (1.15 – 1.24) |
| **Systemic** | 1.09  (0.97 – 1.23) | 1.12  (0.98 – 1.27) |
| **Urinary** | 1.27  (1.15 – 1.40) | 1.27  (1.14 – 1.41) |
| **Valve** | 1.17  (1.10 – 1.25) | 1.19  (1.11 – 1.27) |

Table 64| Fully adjusted odds ratios for the development of each fibrotic condition in the three years post index date. Fully adjusted analyses included adjustment for age, sex, smoking status, deprivation, number of fibrotic conditions at baseline and hypertension severity. This sensitivity analysis included body mass index (BMI) as a covariate, where findings differ these are made apparent with red text.

|  | **Metformin prescription** | |
| --- | --- | --- |
|  | **Fully adjusted**  **OR (99% CI)** | **Fully adjusted**  **OR + BMI (99% CI)** |
| **Atherosclerosis** | 1.02  (0.95 – 1.10) | 1.04  (0.96 – 1.12) |
| **Biliary** | 0.91  (0.83 – 0.99) | 0.90  (0.82 – 0.98) |
| **Blood vessel** | 1.00  (0.90 – 1.12) | 0.95  (0.84 – 1.07) |
| **Cardiomyopathy** | 1.06  (1.01 – 1.11) | 1.07  (1.02 – 1.12) |
| **Integumentary** | 0.96  (0.88 – 1.04) | 0.93  (0.85 – 1.02) |
| **Intest/ panc** | 1.02  (0.99 – 1.06) | 1.03  (0.99 – 1.07) |
| **Liver** | 1.05  (1.00 – 1.11) | 1.06  (1.00 – 1.13) |
| **Lung** | 1.00  (0.96 – 1.04) | 1.01  (0.97 – 1.05) |
| **Reproductive** | 1.00  (0.87 – 1.16) | 1.04  (0.90 – 1.22) |
| **Skeletal** | 1.00  (0.97 – 1.03) | 0.99  (0.95 – 1.02) |
| **Systemic** | 0.97  (0.86 – 1.09) | 0.94  (0.82 – 1.06) |
| **Urinary** | 0.95  (0.86 – 1.04) | 0.94  (0.85 – 1.04) |
| **Valve** | 1.02  (0.96 – 1.08) | 1.02  (0.96 – 1.08) |
