# Supplementary material for: Association between hyperglycaemia, diabetes complications and development of fibrotic conditions among people living with type 1 and type 2 diabetes in England: a retrospective cohort study using UK Clinical Resource Datalink Aurum and Hospital Episode Statistics
Source: BMJ Open. 2025 Oct 27;15(10):e103426. doi: 10.1136/bmjopen-2025-103426 (PMC12570948; doi:10.1136/bmjopen-2025-103426)
Supplement: Supplementary file 2 [file bmjopen-2025-103426.reviewer_comments.pdf]

PEER REVIEW HISTORY

BMJ Open publishes all reviews undertaken for accepted manuscripts. Reviewers are asked to complete a checklist review form and are provided with free text boxes to elaborate on their assessment. These free text comments are reproduced below.

ARTICLE DETAILS

Title (Provisional)

Association between hyperglycaemia, diabetes complications and development of fibrotic conditions among people living with type 1 and type 2 diabetes in England: a retrospective cohort study using UK Clinical Resource Datalink Aurum and Hospital Episode Statistics

Authors

Massen, Georgie May; Cook, Sarah; Moss, Samuel T; Chambers, Rachel; Jenkins, Gisli; Allen, Richard J; Wain, Louise V; Stewart, Iain; Oliver, Nick; Morganstein, Daniel L; Quint, Jennifer K; Consortium, DEMISTIFI

VERSION 1 - REVIEW

**Reviewer**	**1**
**Name**	**Abou-Samra, Abdul Badi**
**Affiliation**	**Hamad Medical Corporation, Qatar Metabolic Institute**
**Date**	**30-Jun-2025**
**COI**	**None**

General:

This is an interesting study using a large dataset showing association of T1D, T2D and their complication with fibrotic conditions. The study uses a comprehensive statistical approach with sensitivity analyses to show association.

The design is retrospective; association does not imply causation, despite the sensitivity analyses there is always residual confounding that is not considered. For example, unmeasured lifestyle factors influence both the diseases, the complication and the fibrotic conditions. BMI data were not included because of missing data. Prescriptions of insulin and metformin does not inform about adherence. The limitations can be expanded to include for example: "Although we adjusted for smoking, deprivation, and comorbidities, unmeasured factors (e.g., physical activity, dietary patterns, or medication adherence) may residually confound observed associations."

The immortal time bias in this retrospective cohort that is not addressed. Sicker patients who died before 2018 are not included in the analysis and thus underestimates the association with fibrotic conditions; and the healthier patients are more likely to survive to 2018 and overestimate the protective effects of Metformin. Perhaps a propensity score matching could resolve such bias; or at least this bias should be acknowledged in the limitation such as “Because our cohort required survival and continuous enrollment until 2018, immortal time bias may have led to underestimation of associations in high-risk patients who died earlier”

The discussion overstates clinical implication and suggest expansion of screening without cost-effect and feasibility. Some mechanistic claims (e.g., telomere length in fibrosis) lack direct citations.

Minor:

Page 7:

• Line 1: The sentence starting with “people had ……” is missing something.

• Line 1: is the date 01/01/2014 correct or is it 01/01/2015?

• Line 17: reference to supplementary figure 2 is not correct. This figure does not have eGFR or ACR on it.

Pages 17 through 20 do not read well as part of the results as they refer frequently to the supplementary figures and tables and would confuse the reader. It is better to summarize.

Page 23, line 17: been performed by the result of the test…. □ been performed but the result of the test

The texts and labels of the figures are made with small size fonts; difficult to read; this reviewer could not see well the texts on the figures. Perhaps the quality provided through the pdf is low and definition is lost when enlarged. I suggest to use larger font that can be read easily.

Some references are incomplete (e.g., "Cellular and molecular mechanisms of fibrosis - Wynn - 2008" lacks full journal details).

**Reviewer**	**2**
**Name**	**Hartopo, Anggoro**
**Affiliation**	**Universitas Gadjah Mada, Cardiology and Vascular Medicine**
**Date**	**21-Sep-2025**
**COI**	**None**

The authors have performed an extensive populational study using the large database of patients in U.K. The study has important message and confirm previous notion that DM, both type 1 and type 2, increased risk of chronic disease (reflected by fibrotic condition). The early occurrence of complication is also associated with the increased risk (higher RRR) to develop chronic condition (organ fibrosis).

There are several suggestion to improve the manuscript:

1. Introduction

- Please explicitly define the aim/purpose of this study

2. Method

- Please define the Research Methodology of this study

- Please define the inclusion and exclusion criteria of subjects

3. The magnitude of risk to develop fibrotic condition is higher in Type 1 DM as compared to type 2 DM. Is this data also statistically analyze ?

4. Discussion

- The first paragraph : the highlight of important finding of the study and whether the hypothesis proven or unproven.

- Add the discussion about the difference RRR between type 1 DM and type 2 DM to predict the fibrotic condition and potential patomechanism.

VERSION 1 - AUTHOR RESPONSE

Reviewer: 1 Prof. Abdul Badi Abou-Samra, Hamad Medical Corporation, Weill Cornell Medicine

This is an interesting study using a large dataset showing association of T1D, T2D and their complication with fibrotic conditions. The study uses a comprehensive statistical approach with sensitivity analyses to show association.

The design is retrospective; association does not imply causation, despite the sensitivity analyses there is always residual confounding that is not considered. For example, unmeasured lifestyle factors influence both the diseases, the complication and the fibrotic conditions. BMI data were not included because of missing data. Prescriptions of insulin and metformin does not inform about adherence. The limitations can be expanded to include for example: "Although we adjusted for smoking, deprivation, and comorbidities, unmeasured factors (e.g., physical

activity, dietary patterns, or medication adherence) may residually confound observed associations."

Thank you for your review, we really appreciate your thorough interrogation of our study, with respect to the above comment we have added the following to our limitations section. 'With regards to the main covariates used in analysis, we believe these were well posed as in the fully adjusted models, the effect sizes were smaller however, residual confounding (such as physical activity, dietary patterns and medication adherence) may explain some associations.'

The immortal time bias in this retrospective cohort that is not addressed. Sicker patients who died before 2018 are not included in the analysis and thus underestimates the association with fibrotic conditions; and the healthier patients are more likely to survive to 2018 and overestimate the protective effects of Metformin. Perhaps a propensity score matching could resolve such bias; or at least this bias should be acknowledged in the limitation such as "Because our cohort required survival and continuous enrollment until 2018, immortal time bias may have led to underestimation of associations in high-risk patients who died earlier"

Thank you for highlighting this important issue, we have added the following: 'It is important to acknowledge that when conducting logistic regression analysis, only people with a minimum of three years of follow-up (therefore must be alive and continuously enrolled at their GP practice) were included in this analysis. As a result, immortal time bias may have lead to an underestimation of associations as high-risk patient may have died within this time and therefore would not contribute data.'

The discussion overstates clinical implication and suggest expansion of screening without cost-effect and feasibility. Some mechanistic claims (e.g., telomere length in fibrosis) lack direct citations.

Thank you, we have tried to ensure that the discussion does not focus on direct clinical implications and instead focuses more on the further research which is required prior to integration of knowledge in clinical practice.

We have added the following clarification regarding screening, the end of the section now reads 'Many important questions must be first explored, for instance which screening processes should be employed, the cost effectiveness as well as the feasibility of altering current screening protocols, the regularity of screening and the establishment of required baseline characteristics to qualify for screening (e.g. age, presence of diabetes complications). This would be a significant undertaking requiring the expertise of multiple disciplines.'

Thank you for this comment, we have added the following references:

38 Cheng F, Carroll L, Joglekar MV, *et al.* Diabetes, metabolic disease, and telomere length. *Lancet Diabetes Endocrinol*. 2021;9:117–26. doi: 10.1016/S2213-8587(20)30365-X

39 Wang J, Dong X, Cao L, *et al.* Association between telomere length and diabetes mellitus: A meta-analysis. *J Int Med Res*. 2016;44:1156–73. doi: 10.1177/0300060516667132

41 Zhao J, Miao K, Wang H, *et al.* Association between Telomere Length and Type 2 Diabetes Mellitus: A Meta-Analysis. *PLOS ONE*. 2013;8:e79993. doi: 10.1371/journal.pone.0079993

Page 7:

• Line 1: The sentence starting with "people had ……" is missing something.

Thank you, you are correct. We have added the following: 'More study specific criteria required that people had to be enrolled at their GP before the 01/01/2015 and remain enrolled until at least 31/12/2018, the patient's data had to be eligible for linkage to HES APC and IMD.'

• Line 1: is the date 01/01/2014 correct or is it 01/01/2015?

Thank you for spotting this, you are correct and we have amended the sentence.

• Line 17: reference to supplementary figure 2 is not correct. This figure does not have eGFR or ACR on it.

Thank you, I have now updated this figure to include measurements of single eGFR and ACR measurements.

Pages 17 through 20 do not read well as part of the results as they refer frequently to the supplementary figures and tables and would confuse the reader. It is better to summarize.

Thank you, we have tried to be as succinct as possible whilst directly referring to the many supplementary tables and figures (as required by the journal). We have further refined these pages (please see the submitted tracked changes document). We believe it is important to highlight the results of the specific sensitivity analyses.

Page 23, line 17: been performed by the result of the test.... ◊ been performed but the result of the test

Thank you we have corrected this.

The texts and labels of the figures are made with small size fonts; difficult to read; this reviewer could not see well the texts on the figures. Perhaps the quality provided through the pdf is low and definition is lost when enlarged. I suggest to use larger font that can be read easily.

Thank you I have remade figures one and two to better improve the quality

Some references are incomplete (e.g., "Cellular and molecular mechanisms of fibrosis - Wynn - 2008" lacks full journal details).

Thank you, I believe I have corrected all instances of this.

Reviewer: 2 Dr. Anggoro Hartopo, Universitas Gadjah Mada

The authors have performed an extensive populational study using the large database of patients in U.K. The study has important message and confirm previous notion that DM, both type 1 and type 2, increased risk of chronic disease (reflected by fibrotic condition). The early occurrence of complication is also associated with the increased risk (higher RRR) to develop chronic condition (organ fibrosis).

Thank you for your thorough and thoughtful review, we are very appreciative.

There are several suggestion to improve the manuscript:

1. Introduction

- Please explicitly define the aim/purpose of this study

Thank you, we have added the following 'By conducting this retrospective cohort study we aimed to explore whether diabetes complications and severity are associated with fibrotic conditions.'

2. Method

- Please define the Research Methodology of this study

Thank you, we have amended the first sentence of the results to read 'To conduct this retrospective cohort study, we used the December 2023 build of the Clinical Practice Research Datalink Aurum database.'

- Please define the inclusion and exclusion criteria of subjects

Thank you, we have reformatted and rewritten the following paragraph as a result of your comment 'Broad inclusion criteria required that people had to be at least 18 years of age, have a recorded sex (the number of indeterminate cases was low and therefore excluded) and people had to have at least one record of T1DM or T2DM in their primary care records between the 01/01/2015 and 31/12/2015 (baseline period). More study specific criteria required that people had to be enrolled at their GP before the 01/01/2015 and remain enrolled until at least 31/12/2018, the patient's data had to be eligible for linkage to HES APC and IMD. Two discrete populations (T1DM and T2DM respectively) were used throughout this study and at no point combined **(Supplementary figure 1)**. The index date was the 31/12/2015 **(Supplementary figure 2)**.'

3. The magnitude of risk to develop fibrotic condition is higher in Type 1 DM as compared to type 2 DM. Is this data also statistically analyze ?

We did not look to understand how the relative risk of single and multiple fibrotic conditions differed between people with type one and type two diabetes and their respective complications. This was not an aim of the study, and we believe that this is a separate research question.

4. Discussion

- The first paragraph : the highlight of important finding of the study and whether the hypothesis proven or unproven.

Thank you for highlighting that we have not done this, we have added the following: 'This study supports the hypothesis (specifically in people with T2DM) that more significant hyperglycaemia, attenuated response to hypoglycaemic therapies and greater propensity to complications are associated with fibrosis'

- Add the discussion about the difference RRR between type 1 DM and type 2 DM to predict the fibrotic condition and potential patomechanism.

Thank you, following from the response above, we have not added this to the discussion. However, we have detailed throughout the discussion the possible reasons for the observed associations between diabetes and fibrotic conditions.

VERSION 2 - REVIEW

**Reviewer**	**1**
**Name**	**Abou-Samra, Abdul Badi**
**Affiliation**	**Hamad Medical Corporation, Qatar Metabolic Institute**
**Date**	**04-Oct-2025**
**COI**	

Labels and texts on Figures 2 and 3 are still very tiny and low resolution. The authors say they have made them much better. However, I see in the proof the same old figures. I do not find a separate figures files. Perhaps the authors submitted to the editor such files. Please make sure that the figures are eligible before publication.
